# An interactive, online decision aid assessing patient goals and preferences for treatment of aortic stenosis to support physician-led shared decision-making: Early feasibility pilot study

**DOI:** 10.1371/journal.pone.0302378

**Published:** 2024-05-21

**Authors:** Megan Coylewright, Diana Otero, Brian R. Lindman, Melissa M. Levack, Aaron Horne, Long H. Ngo, Melissa Beaudry, Hannah V. Col, Nananda F. Col

**Affiliations:** 1 Department of Cardiovascular Medicine, University of Tennessee Health Science Center College of Medicine-Chattanooga, Chattanooga, Tennessee, United States of America; 2 Department of Cardiovascular Medicine, Columbia University Medical Center, New York, NY, United States of America; 3 Structural Heart and Valve Center, Vanderbilt University Medical Center, Nashville, Tennessee, United States of America; 4 Department of Cardiac Surgery, Vanderbilt University Medical Center, Nashville, Tennessee, United States of America; 5 Department of Medicine, Summit Health, Berkeley Heights, NJ, United States of America; 6 Harvard Medical School, Boston, Massachusetts, United States of America; 7 Central Vermont Medical Center, Berlin, Vermont, United States of America; 8 Shared Decision Making Resources, Georgetown, ME and Beth Israel Deaconess Medical Center, Boston, Massachusetts, United States of America; 9 Shared Decision Making Resources, Georgetown, ME and University of New England, Biddeford, Maine, United States of America; Public Library of Science, UNITED KINGDOM

## Abstract

**Background:**

Guidelines recommend shared decision making when choosing treatment for severe aortic stenosis but implementation has lagged. We assessed the feasibility and impact of a novel decision aid for severe aortic stenosis at point-of-care.

**Methods:**

This prospective multi-site pilot cohort study included adults with severe aortic stenosis and their clinicians. Patients were referred by their heart team when scheduled to discuss treatment options.

Outcomes included shared decision-making processes, communication quality, decision-making confidence, decisional conflict, knowledge, stage of decision making, decision quality, and perceptions of the tool. Patients were assessed at baseline (T0), after using the intervention (T1), and after the clinical encounter (T2); clinicians were assessed at T2.

Before the encounter, patients reviewed the intervention, Aortic Valve Improved Treatment Approaches (AVITA), an interactive, online decision aid. AVITA presents options, frames decisions, clarifies patient goals and values, and generates a summary to use with clinicians during the encounter.

**Results:**

30 patients (9 women [30.0%]; mean [SD] age 70.4 years [11.0]) and 14 clinicians (4 women [28.6%], 7 cardiothoracic surgeons [50%]) comprised 28 clinical encounters Most patients [85.7%] and clinicians [84.6%] endorsed AVITA. Patients reported AVITA easy to use [89.3%] and helped them choose treatment [95.5%]. Clinicians reported the AVITA summary helped them understand their patients’ values [80.8%] and make values-aligned recommendations [61.5%]. Patient knowledge significantly improved at T1 and T2 (p = 0.004). Decisional conflict, decision-making stage, and decision quality improved at T2 (p = 0.0001, 0.0005, and 0.083, respectively). Most patients [60%] changed treatment preference between T0 and T2. Initial treatment preferences were associated with low knowledge, high decisional conflict, and poor decision quality; final preferences were associated with high knowledge, low conflict, and high quality.

**Conclusions:**

AVITA was endorsed by patients and clinicians, easy to use, improved shared decision-making quality and helped patients and clinicians arrive at a treatment that reflected patients’ values.

**Trial registration:**

Trial ID: NCT04755426, Clinicaltrials.gov/ct2/show/NCT04755426.

## Introduction

The expanding indications for transcatheter aortic valve replacement (TAVR) procedures, now available to patients of all surgical risk levels [1] and for repeat procedures, has complicated decision making for severe aortic stenosis (sAS). Surgical aortic valve replacement (SAVR) and TAVR have comparable survival rates but different impacts on quality of life; medical options alone have high associated mortality [1]. In younger patients with long life expectancy, mechanical valves may be recommended as they typically last a lifetime, though they require lifelong anticoagulation and can only be implanted surgically. Nonetheless, most patients undergoing SAVR receive bioprosthetic valves, which are less durable than mechanical valves. TAVR is increasingly offered to patients as the less invasive procedure when bioprosthetic valves are used, and this can include those whose remaining life expectancy may exceed the durability of the prosthetic valve [2]. Decisions about treating sAS are preference-sensitive and should reflect how patients value the consequences of treatment, including recovery, long-term disease trajectories, and need for repeat procedures.

The introduction of the heart team approach for choosing sAS treatment [3] adds further complexities to incorporating patient preferences into treatment decisions. Heart teams typically include valve specialists (interventional cardiologists, surgeons, and potentially other clinicians) who collaboratively select the best treatment to offer the patient at a valve meeting separate from the initial patient consultation.

Clinical guidelines on valvular heart disease give shared decision-making (SDM) their highest recommendation [4], but best practices and implementation strategies are not clearly defined. SDM is an interactive process where clinicians share their expertise about the condition and treatments, while patients share their goals and values regarding treatment, arriving at a shared decision that reflects informed patient preferences [5]. Patient decision aids (DAs) are evidence-based tools designed to facilitate SDM. Currently there are few DAs for patients with sAS and SDM is infrequently implemented [6].

This study is part of a larger mixed-methods study to develop a DA tool to support SDM for patients with sAS in real-world settings and used at point-of-care. We initially identified and prioritized patient goals, values, and preferences for sAS treatments [7]. We used those findings to develop a SDM tool intended to help patients with sAS clarify their values, arrive at an informed treatment preference, and communicate their values and preferences to their clinicians, modeled on previously validated tools [8,9]. The DA, AVITA (Aortic Valve Improved Treatment Approaches), targets patients and their heart team clinicians. AVITA includes theory-based design elements (including image theory) to catalyze patient engagement and improve decision-making. Patients interact with AVITA prior to their heart team encounter; AVITA transmits a summary of the patient’s values and preferences to heart team clinicians before clinic deliberations. The pilot testing described here is consistent with international standards for developing Das [10], which recommend testing among patients and clinicians in real-world settings prior to utilization or further testing.

The aim of this pilot study was to evaluate the acceptability of AVITA to patients and their heart team clinicians, the feasibility for its integration in a range of representative clinical settings, and its preliminary impact on key SDM processes and treatment decisions in patients and clinicians engaged in real-time decision-making for sAS. Findings will guide its broader implementation and evaluation in clinical settings.

## Materials and methods

### Design, setting, and participants

We conducted a pilot multi-center prospective nonrandomized cohort study among patients with sAS who were actively choosing treatment and their heart team clinicians. All participants were allocated to receive AVITA; patients were assessed before and immediately after using the tool and again following the clinical encounter/s with their heart team clinician to discuss treatment options. Patients had clinical encounters with different heart team clinicians (interventional cardiologists and cardiac surgeons, as mandated by Medicare), whether seen separately or during the same appointment. We prioritized capturing the second appointment when more information would be available to both the patient and physician. Heart team clinicians were assessed after the clinical encounter with a participating patient. A pre-post design was selected because it is more sensitive and valid than a randomized controlled trial when testing an intervention that prompts a change in the frame of reference that participants use to assess their attitudes [11–14]. Neither patients nor their clinicians were blinded.

English-speaking adults (≥ 18 years) in the US with internet access and an email address were eligible if they had sAS, faced a treatment decision, and had an upcoming appointment with a heart team clinician to discuss treatment options. Physicians, surgeons, or Advanced Practice Providers (APPs) of participating patients were eligible if they were engaged in decision-making for sAS.

Patient participants were referred to the study between March 12 and July 11, 2022 from five clinical sites purposefully selected to reflect geographic (TN (2), KY, WA, VT) and practice setting diversity as well as the presence of a site champion. One site (VT) provided secondary care with a general cardiology nurse practitioner engaging patients earlier in the SDM process, and four provided tertiary care, with a heart team comprised of interventional cardiologists and cardiac surgeons engaging with patients in final treatment decisions. The secondary care site was selected to assess the feasibility of patient engagement at an earlier stage.

Potentially eligible patients were identified by the site cardiovascular teams, which were comprised of clinicians treating patients with valvular heart disease and their valve coordinators. Eligible patients had sAS and were deemed by the heart team to have an active choice regarding treatment options (i.e., TAVR or SAVR). Patients who responded to the study invitation were further screened for eligibility online.

Each site was encouraged to determine the most effective local solution to patient recruitment for the study. Heart team valve coordinators were engaged in conceptualization of study design and study implementation. It was determined that patients would be mailed or emailed information about the study; given business cards with study information during preceding clinic visits before meeting with heart team physicians; or contacted by phone.

All patient and clinician participants provided written informed consent (online); the study was overseen by Western Institutional Review Board (WIRB^®^) now known as WIRB-Copernicus Group® (WCG) IRB. The study followed the Transparent Reporting of Evaluations with Nonrandomized Designs (TREND) Standards.

#### Intervention

AVITA is an online, interactive, patient-centered DA designed for patients and their clinicians. AVITA is informed by International Patient Decision Aid Standards (IPDAS) criteria [15], relevant theory (S2.1 Table in S1 File) [16,17], and extensive formative work [7]. Its design was informed by image theory [18], a descriptive theory that has been validated in a variety of settings [19] and endorsed for SDM [20]. Image theory describes decision-making as a 2-step process that is guided by the decision-makers’ beliefs and values. The first and most critical step is screening-out options that seem incompatible with the decision-maker’s values and goals, a process that focuses on negative attributes of the options. The second step involves choosing the best option from any remaining items by examining their pros and cons. To minimize premature elimination of viable options in step one, AVITA clarifies patient goals and values before discussing options. AVITA also guides patients through other key elements of SDM (e.g., assessing their preferred decision-making role, describing available treatment options, and framing the trajectory of decisions) but does not make a treatment recommendation. It is designed to help patients clarify their own values with regard to the treatment decision and to encourage and facilitate patient-clinician communication through a patient-specific summary of the patient’s goals, values, and preferences that is emailed to the patient and shared with the patient’s clinician/s. Patients interact with AVITA online (at a location of their choosing) once, before the clinical encounter.

Patients accessed the online tool via a link. AVITA guides patients through a series of tailored questions and feedback about aortic stenosis (S8-S12 Figs in S2 File). Using findings previously generated from structured focus groups (using the Nominal Group Technique) with patients with sAS who had undergone decision-making, a comprehensive list of goals and preferences for decision-making were offered to the patient for reflection (S10 Fig in S2 File). Patients choose which of the goals and preferences were most important to them (and could add others), with this information used in the final patient summary that was later shared with the heart team prior to the clinical encounter. This summary was emailed to the clinician and typically printed by the valve coordinator and placed outside the patient’s room along with their vital signs.

Designated heart team clinicians did not interact directly with AVITA—they simply reviewed their patient’s summary (printed or online) either before or during the encounter. The summary was framed to help patients share their values and informed preferences and to help clinicians incorporate their patient’s values into treatment decisions at point-of-care (S12 Fig in S2 File; screenshot of a sample patient summary report). AVITA was designed to require 10–20 minutes for patients to complete online and 2–5 minutes for clinicians to review the summary. Patients did not need to complete AVITA all at once; they could log back in later to complete the tool. Participants received a modest stipend after completing the intervention and the follow-up survey.

### Outcomes

Patients completed assessment surveys at 3 time points: just prior to receiving the AVITA intervention (T0), immediately following using AVITA (T1), and after the clinical encounter (T2). Clinicians completed a single survey at T2 for each participating patient. All outcomes were prespecified. All surveys were online. Data were entered directly by participants.

#### Patient-reported outcomes

SDM is a multidimensional process involving patients and clinicians that includes fostering choice awareness, discussing pros and cons of all treatment options, discussing patient values and preferences, and making decisions [21]. No single measure captures all dimensions of SDM [22]. To obtain a holistic picture of the effect of AVITA on SDM, our primary outcome measure, we evaluated SDM using several scales at different time points and from different perspectives (patients vs clinicians). The SDM Process scale [23], adapted to assess 2 options (SAVR and TAVR), was assessed at T2. This scale elicited patient perspectives on whether clinicians offered choices, discussed treatment pros and cons, and asked if the patient wanted TAVR or SAVR. The original 4-item scale addressed decisions involving only one option, with a maximum score of 4. Our adapted 6-item scale addresses two options, with a maximum score of 6, and can be normalized, allowing for comparison to the original scale.

Patient perspectives on clinician communication was assessed using the General Communication subscale [24] (0–10, 10 indicates best communication) and Patient Experience Measures from the Consumer Assessment of Healthcare Providers and Systems (CAHPS) Surgical Care Survey [25]. Communication items were coded as clear (yes, definitely) and unclear (somewhat or no). Decisional conflict was assessed at T0 and T2 using the 4-item Decisional Conflict Scale [26] (range 0–4, <4 indicates conflict). Stage of Decision-Making [27] was assessed at T0 and T2, using a 5-point scale, from *not thinking about choices* (0) to *decided* (5). Preferred involvement in decision-making was assessed at T1 (the first question in AVITA) and T2, using the Control Preferences Scale [28]. Responses ranged from patient-dominated to clinician-dominated. Five true/false questions assessed patient knowledge about sAS and its treatment. Items were adapted from a previously used knowledge test [29]. Each correct answer was scored as 1 point, an incorrect response as 0; scores ranged from 0–5; higher scores indicate greater knowledge (S13 Fig in S2 File). Two separate questions, drawn from a previously published survey [30], assessed self-perceived knowledge about sAS and treatment (“*how would you rate your knowledge of the following*: (1) *Your aortic stenosis*. (2) *Your options for treating aortic stenosis*”). Response options ranged from “*Not knowledgeable at all*” to “*very knowledgeable*.” Patient preference for treatment was assessed at T0, T1, and T2. At T0 and T1, patients were asked which treatment they preferred, whereas at T2 they were asked “*which treatment have you chosen for your aortic stenosis*”. We sought to capture the patient’s chosen treatment following the heart team encounter, rather than which treatment was eventually performed. Response options were TAVR (Transcatheter aortic valve replacement), SAVR (Surgical aortic valve replacement), no valve replacement (medications and/or comfort care), and Unsure. Decision quality, assessed at T0 and T2, asked whether the patient’s treatment plan reflects what’s important to them (responses used a 5-point Likert scale, strongly disagree to strongly agree).

Just after viewing AVITA (T1), patients evaluated AVITA’s usability, trustworthiness, perceived helpfulness in various areas (values clarification, stimulating engagement, understanding that there are treatment choices, understanding reasons for having or not having TAVR, and being able to talk with their clinician about what matters most to them), and if they would recommend it to others, using a 5-point Likert scale (strongly agree to strongly disagree). The impact of AVITA on decision-making confidence was assessed at T1 using the Decision Self-efficacy scale [31,32], asking if AVITA changed how confident they felt about the various domains assessed. Responses were coded as confident (yes, a lot, or a little) or not.

### Clinician-reported outcomes

After each encounter (T2), clinicians were asked if and how they received the AVITA summary, how they would like to receive it, if they reviewed it, how many minutes they spent reviewing it, and if they felt the visit was more (or less) efficient because of the AVITA tool (response options were more efficient, no change, less efficient). Clinicians were asked what recommendation they were leaning towards for the patient at that time, and, during the visit with a participating patient, what impact AVITA had on their communication with the patient, their knowledge of what’s important to their patient, their ability to engage the patient in decision-making, and their ability to make a valve recommendation based on what’s important to the patient recommendation based on what matters most to their patient (response options were *worsened*, *no impact*, *improved*). If “*worsened*” was selected, a free-text question elicited more information about why. After being shown a definition of SDM, clinicians were asked if they practiced SDM with their patient during that encounter, with response options being ‘yes’ or ‘no’. Those responding ‘yes’ were asked “Why did you practice shared decision-making with [name]?” and shown a drop-down list of 9 items, drawn from the literature and feedback from our clinician advisers, and ‘other’ (a free-text field). Clinicians who responded ‘no’ would be shown a question asking “What were the challenges that you encountered practicing shared decision-making with [name]?” and shown a drop-down list of 9 common challenges to SDM. Clinicians were also asked if they would use the AVITA summary in future encounters.

### Assessing feasibility

Feasibility is not well-defined in the SDM literature [33]. In this study, we determined feasibility to be evidenced by three features. This included acceptability of the DA by patients and clinicians; ability to incorporate the DA into real-world clinical workflows; and improved SDM and/or decisional quality using formal measures. In prior research of cardiovascular DAs, feasibility of DAs has been limited by barriers to engaging patients prior to a clinical encounter when SDM is planned, and clinician reluctance to use a DA during the encounter. We thus assess the feasibility of the AVITA DA using measures that capture the ability to engage patients before the encounter and to engage their clinicians at the time SDM was planned to occur.

The ability to engage patients was assessed by their ability to access the tool on the internet, complete the tool activities, and endorse the content of the tool. Valve coordinators (who typically engaged patients prior to the clinical encounter) did not document the number of patients invited to participate (to reduce study burden). Lacking the number of patients receiving a study invitation, we could not calculate the proportion of patients who responded to the study invitation. Instead, we estimated the overall penetrance of the tool in clinics by dividing the number of patients using AVITA by clinic-reported TAVR volumes.

The feasibility of engaging diverse heart team clinicians to use the AVITA-generated summary during a clinical encounter *at the time SDM occurs* was assessed by surveys completed by both patients and clinicians just after the clinic encounter (T2), reporting on if and how AVITA was used in heart team clinics. This was reliant on two contributions: the valve coordinator making the AVITA summary available to the clinician at the time of the visit, and the clinician incorporating the AVITA summary into the visit. These T2 surveys also assessed the efficacy of the intervention from both patient and clinician perspectives, using measures of SDM and decisional quality.

### Sample size

The target pilot sample of 25 real-world patient-clinician pairs (n = 50 total) was predetermined. It was not based on power analysis but reflected pragmatic constraints (costs and time), previous beta-testing [8,9], and studies suggesting that 12–50 is generally sufficient to assess survey tools [34,35], cognizant that we sought to frugally but efficiently evaluate the stand-alone DA on multiple outcomes prior to pursuing broader implementation and integration into clinical settings.

### Statistical analysis

The impact of AVITA was assessed using pre-post comparisons for those completing all or part of AVITA. For variables assessed at three time points, we calculated changes in means over time, using linear mixed-effect models where the within-subject correlations due to longitudinal measurements for each subject at time points T0, T1, T2 were modeled using autoregressive lag-1 variance-covariance structure, and restricted maximum likelihood was used to obtain estimated mean differences among time points. We assessed assumptions for normality and used nonparametric models (Wilcoxon rank-sum test) when the distribution of the parameter of interest deviated from the normal distribution (e.g., analyses of knowledge and self-assessed knowledge). Agreement between ordinal variables was estimated using kappa statistic. We used paired t-tests to test for the differences when the data distribution was normally or near normally distributed (e.g., analyses of stage of decision-making, decisional conflict, and decision quality where the parameter of interest is the change between T0 and T2). Correlations (e.g., between actual and perceived knowledge) were calculated using the Spearman correlation coefficient. Analyses were done with SAS V9.4 statistical software (SAS Institute Inc, Cary, NC, US).

## Results

### Individual site dissemination strategies

Potentially eligible patients were contacted via a post office mailing, an email or telephone invitation, or handed a referral card in the cardiology office; all contact materials were IRB approved. One site had a pre-existing protocol of sending a post office mailing to patients prior to their heart team visit, and study information was added to this packet. One site proactively reviewed incoming patients for heart team visits and contacted patients with an email address listed in the electronic medical record (EMR). The remaining three sites either telephoned the patients prior to the visit or discussed the study at the patient’s first encounter with a heart team member, either a general cardiology clinician or an interventional cardiologist, and implemented the use of the DA prior to the upcoming heart team visit.

Once patients completed AVITA, each site individually selected the best way that heart team clinicians could receive the summary page generated by AVITA for use at the time of the clinical encounter in which SDM was to take place. Nearly all sites tasked the valve coordinator or medical assistant with receiving an email of the AVITA summary page, printing it, and delivering it to the clinician just-in-time for the clinical encounter in which SDM was occurring.

### Patient and clinician characteristics

**Fig 1:**
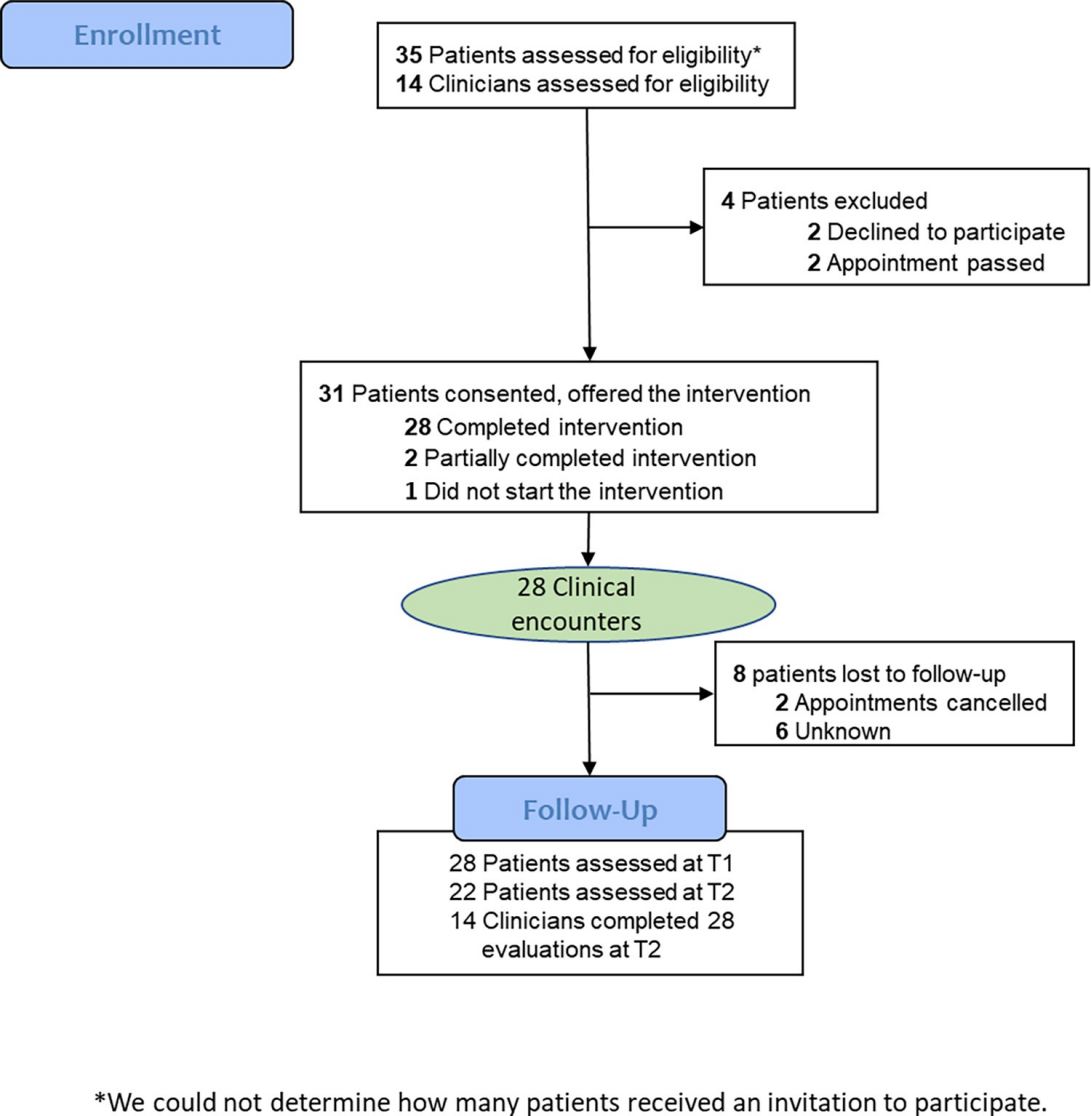
CONSORT diagram. * We did not require recording of the number of people who received an invitation to participate via either post office mail, email, telephone or hand-delivered information.

31 patients and 14 clinicians consented to participate, with a total of 28 clinical encounters (Fig 1). One patient did not start AVITA, 29 completed AVITA (1 of whom partially completed the evaluation), and 1 partially completed AVITA (spending 11 minutes). Patients’ mean [SD] age was 70.4 years [11.0] and 9 [30.0%] were female (Table 1; S1 Table in S1 File), reflecting the epidemiology of sAS [36]. Five patients who did not complete the full intake screening had a similar age (mean 70.6, range 27–91) to those who completed screening.

**Table 1:** Participant characteristics at baseline.

Patient Participants (n = 30)*	No. (%)
Mean patient age, years (median, range)	70.4 (SD 11.0; median 68; 37–90)
**Gender**, n (%)	
Male	21 (70.0)
Female	9 (30.0)
**Race** n (%)	
White/Caucasian	26 (86.7)
Black/African American, Hispanic	4 (13.3)
**Years since AS diagnosis**, average (SD, range)	5.25, median 1.9 (SD 10.16; range 1–56)
**Education**, n (%)	
Less than high school	3 (10.0)
High school or GED	11 (36.7)
Some college	7 (23.3)
2-year college or technical school	3 (10.0)
College graduate	5 (16.7)
Graduate school or professional degree	1 (3.3)
**Inadequate Health literacy** ^†^	18 (60.0)
**Preferred decision-making role**	
Make the final decision myself	4 (13.3)
Make the final decision myself after seriously considering HCP’s opinions	8 (26.7)
Share responsibility with HCP	14 (46.7)
Have HCP make the final decision after considering my opinion	4 (13.3)
Leave all decisions to clinician	0 (0)
**Clinician Participants (n = 14**)	**No. clinicians (%)**
**Type of Clinician**	
Interventional cardiologist	6 (42.9%)
Cardiothoracic surgeon	7 (50.0%)
Nurse Practitioner	1 (7.1)
**Years in Practice** (average, SD)	11.9 (SD 9.15); range 1–30
**Affiliated with a structural heart team**	13 (92.86%)
**Gender**, n (%)	
Male	8 (57.1%)
Female	4 (28.6%)
Prefer not to answer	2 (14.3%)

Abbreviations: AS, aortic stenosis; GED, general educational development test, a high school equivalency diploma; HCP, health care practitioner; No, number; SD, standard deviation.

* Only 28 patient participants completed the intervention and T1 evaluation.

^**†**^ % needing help reading hospital materials (sometimes, often or always).

Fourteen different clinicians participated, with an average of 12 years in practice (11.9 [9.15]) and of which one-quarter were women (4 [28.6%]). Cardiovascular clinicians from five institutions included 7 (50%) cardiothoracic surgeons, 6 (42.9%) interventionalist cardiologists, and 1 (7.1%) nurse practitioner.

### Acceptability to patients

The participants who completed AVITA spent a median of 18 minutes on the activity (range 7 minutes to 17.3 hours; range 7–48 minutes after excluding two outliers who presumably took extended breaks; one outlier was logged in for 4.2 hours, another for 17.3 hours). After completing AVITA (T1), patients answered a survey; not all patients completed all questions. Most patients found the tool easy to use (25/28 [89.3%]), trustworthy (27/28 [96.4%]), and recommended it to others (24/28 [85.7%]; Table 2). Patients reported that AVITA made them want to be more involved in decisions (24/27 [88.9%]) and improved their confidence in expressing their concerns (27/27 [100%]) and choosing from treatment options (27/27 [100%]) (S1 and S2 Figs in S1 File). After the clinical encounter in which the DA was used (T2), nearly all patients (21/22 [95.5%]) reported that AVITA helped them talk to their clinician about their goals and preferences and select treatment. In total, patients completed the AVITA DA along with its survey evaluation in a median of 30 minutes (mode 33; average 89.8). The time between T0 (before viewing the DA) and T2 (the clinical encounter) averaged 9.35 days (range 1 to 24.9, median 7.5).

**Table 2:** Selected patient and clinician-reported outcomes^a^.

	After AVITA T1	After encounter T2
**Patient participants**		
**Immediate evaluation of AVITA tool**		
Easy to use	25/28 (89.29)	
Patient recommends tool to others	24/28 (85.71)	
Trustworthy	27/28 (96.43)	
Summary report reflects what matters to the patient	26/27 (96.30)	
Helps patients identify their treatment goals and priorities	24/27 (88.89)	
Helps patients talk to their cardiologist about what matters to them	25/27 (92.59)	
Wanting to be more involved in decisions about treatment	24/27 (88.89)	
Understands they have treatment choices	26/27 (96.30)	
**Improved Confidence in Decision-making** ^b^ ^,^ ^c^		
Understand the information enough to be able to make a choice	25/27 (92.59)	
Ask questions without feeling embarrassed	26/27 (96.30)	
Express your concerns about each choice	27/27 (100.00)	
Ask for advice	27/27 (100.00)	
Figure out the treatment choice that best suits you	27/27 (100.00)	
Handle unwanted pressure from others in making your choice	25/27 (92.59)	
Let the clinic team know what’s best for you	27/27 (100.0)	
**SDM Processes** ^d^		
Clinician explained there were treatment choices		18/22 (81.82)
…explained pros of TAVR^e^		11/22 (50.0)
…explained pros of SAVR^e^		8/22 (36.36)
…explained cons of TAVR^f^		6/22 (27.27)
…explained cons of SAVR^f^		5/22 (22.73)
…asked if patient wanted SAVR or TAVR		14/22 (63.64)
Total score (range 0–6)		3.29 (0–5.5) ^h^
**Quality of Communication** ^g^		
Told patients they have > one option		16/22 (72.73)
Asked which treatment patient preferred		14/22 (63.64)
Spent enough time		18/22 (81.82)
Presented risks and benefits of treatments		17/22 (77.27)
Encouraged questions		19/22 (86.36)
Easy to understand		22/22 (100.00)
Shows courtesy and respect		21/22 (95.45)
Listens carefully		21/22 (95.45)
**Overall Communication** (range 0–10)		9.41 [1.18]
**Patient evaluation of AVITA**		
Helped patient talk to clinician about their goals and preferences		21/22 [95.45]
Helped patient choose treatment for aortic stenosis		21/22 [95.45]
**Clinician-reported outcomes**		
Improved knowledge of what’s important to patient		21/26 (80.77)
Improved ability to engage patient in decision-making		17/26 (65.38)
Improved communication with patient		21/26 (80.77)
improved ability to make a recommendation based on what’s important to the patient		16/26 (61.54)
Influenced clinician recommendation		16/28 (57.14)
Improved efficiency of the encounter		12/25 (48.00)
..No change in efficiency		12/25 (48.00)
..Less efficient		1/25 (4.00)
Clinician would use AVITA in future encounters		22/26 (84.62)

AVITA, Aortic Valve Improved Treatment Approaches; SAVR, Surgical aortic valve replacement; TAVR, Transcatheter aortic valve replacement.

^**a**^ Complete table shown in Table S1; longitudinal findings in S2 Table in S1 File.

^b^ The Decision Self-Efficacy scale was used to assess confidence.

^c^ Percent who reported that AVITA changed their confidence a lot or a little.

^d^ % reporting ‘discussed a lot’.

^e^ “explained reasons to have [TAVR/SAVR]”.

^f^ “explained reasons to not want [TAVR/SAVR]”.

^g^ % responding *“Yes*, *definitely”*.

^h^ The original 4-item SDM Process scale addressed only one treatment option. Our adapted 6-item scale addressed 2 options. Normalized total score: 54.83; score adjusted to a four-point scale: 2.19.

### Acceptability to clinicians

In most encounters, clinicians reported that AVITA helped them communicate with their patient (21/26 [80.8%]), understand what was important to their patient (21/26 [80.8%]), engage patients in decision-making (17/26 [65.4%]) and make a recommendation based on patient values (16/26 [61.5%]). Two clinicians reported that AVITA worsened decision-making (involving two separate patients), noting that the patient wanted a treatment that the clinician thought was inappropriate. Both patients changed their preference to align with their clinician’s recommendation; both reported trusting their clinician’s judgement and gave clinicians excellent evaluations following the discussion supported by the DA.

Clinicians reported spending a median of 5 [4.97] minutes reviewing the AVITA summary that was shared with them. In nearly half of all encounters, heart team physicians reported the AVITA DA made their visit more efficient. Specifically, in 12 of 25 encounters [48%], clinicians reported more efficient encounters, 12/25 (48%) reported no impact, and 1/25 (4%) reported less efficiency. In 22/26 [84.6%] encounters, clinicians reported they would use AVITA in future encounters. The three clinicians who did not recommend AVITA after their first use subsequently recommended it. Clinicians reported that the summary influenced their treatment recommendation in most (16/28 [57.1%]) encounters (14 “a little”, 2 “a lot”) (S3 Fig in S1 File). No adverse events were noted.

### Logistics

Most clinicians reported receiving the summary report via email (16/27 [59.3%]) and/or printed by staff (15/27 [55.6%]), with 1/27 (3.7%) reporting that the patient handed it to them (this clinician reported also receiving it via email). Clinicians evenly preferred receiving it via email (15/28 [39.5%]) or printed (15/28 [53.6%]); only 3/28 [10.7%] preferred the patient deliver it. (Clinicians could select more than one response). None reported that routine use of the tool would be too challenging to coordinate.

#### Feasibility of use by patients

29 of the 35 patients who were assessed for eligibility (29/35 [82.9%)] completed the AVITA DA.

We were limited in understanding the number of patients who were eligible to receive the DA in our study, due to co-design of the study protocol with valve coordinators who were concerned about the burden of recording all patients invited to participate in the study. There are, however, surrogate outcomes to estimate the total number of patients seen for decision-making regarding the treatment of sAS at each site during the study period. Based on general volumes of TAVR at the participating sites, approximately 140 patients were likely evaluated during the four-month recruitment period, and an estimated 60% of patients had access and comfort for internet use of the tool (84 potential patients). This translates to one in five (28/140 [20%]) of all potentially eligible patients, and one in three (28/84 [33.3%]) potentially eligible internet-accessible patients who completed all steps of the implementation of AVITA.

#### Feasibility of use by clinicians

Once patients had completed the AVITA tool, the uptake and review of the AVITA patient summary by clinicians was high (26/28 [92.9%]), with most (25/28 [89.3%]) using the AVITA summary during the clinical encounter. One reported not receiving the summary, one received it but did not review it, one reviewed it after the encounter.

### Differing perspectives on SDM: Clinicians and patients

All clinicians self-reported practicing SDM at every encounter (28/28 [100%]). Clinicians’ most frequently reported reason for practicing SDM was to help patients understand the reasons behind the clinician’s recommendation (21 endorsements) (S6 Fig in S1 File). Because all clinicians self-reported practicing SDM, we were unable to assess why they did not do so.

**Fig 2:**
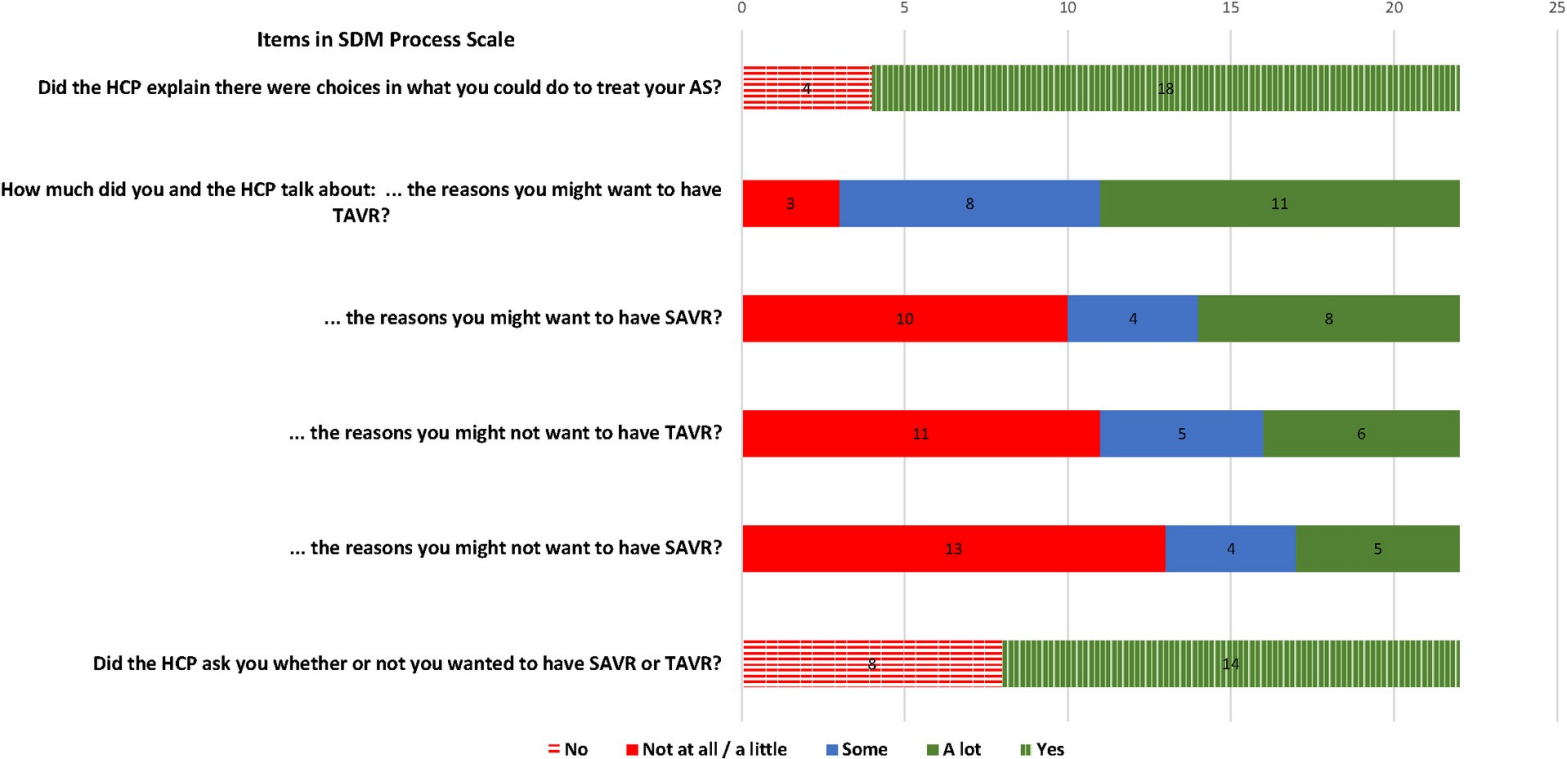
HCP: Healthcare professional; SAVR: Surgical aortic valve replacement; SDM: Shared decision-making; TAVR: Transcatheter aortic valve replacement. Questions were derived from the SDM Process Scale, where the benefits of having TAVR or SAVR were assessed by asking “*the reasons you might want to have [TAVR/SAVR]*” and the risks or cons were assessed by asking “*the reasons you might not want to have [TAVR/SAVR]*”.

In contrast, patients reported that key processes of SDM were often omitted, with none of the 22 patient-evaluated encounters meeting all criteria for SDM. The most omitted SDM processes were discussing the cons of TAVR or SAVR, omitted in 11/22 [50%] and 13/22 [59.1%], respectively, and asking if the patient wanted SAVR or TAVR (omitted in 8/22 [36%]) (Fig 2). Shortfalls in SDM-related communication were also observed in the CAHPS Survey. In 8/22 [36.4%], clinicians did not clearly ask patients which treatment patients thought was best, and in 6/22 [27.3%] clinicians did not share that there was more than one treatment option (S4 Fig in S1 File). In contrast, patients gave excellent ratings to their clinician’s overall communication (mean 9.41 [1.18] on a 0–10 scale) and items relating to clarity, courtesy, respect, and listening (Table 2, S4 and S5 Figs in S1 File).

### Knowledge and decisional quality

**Fig 3:**
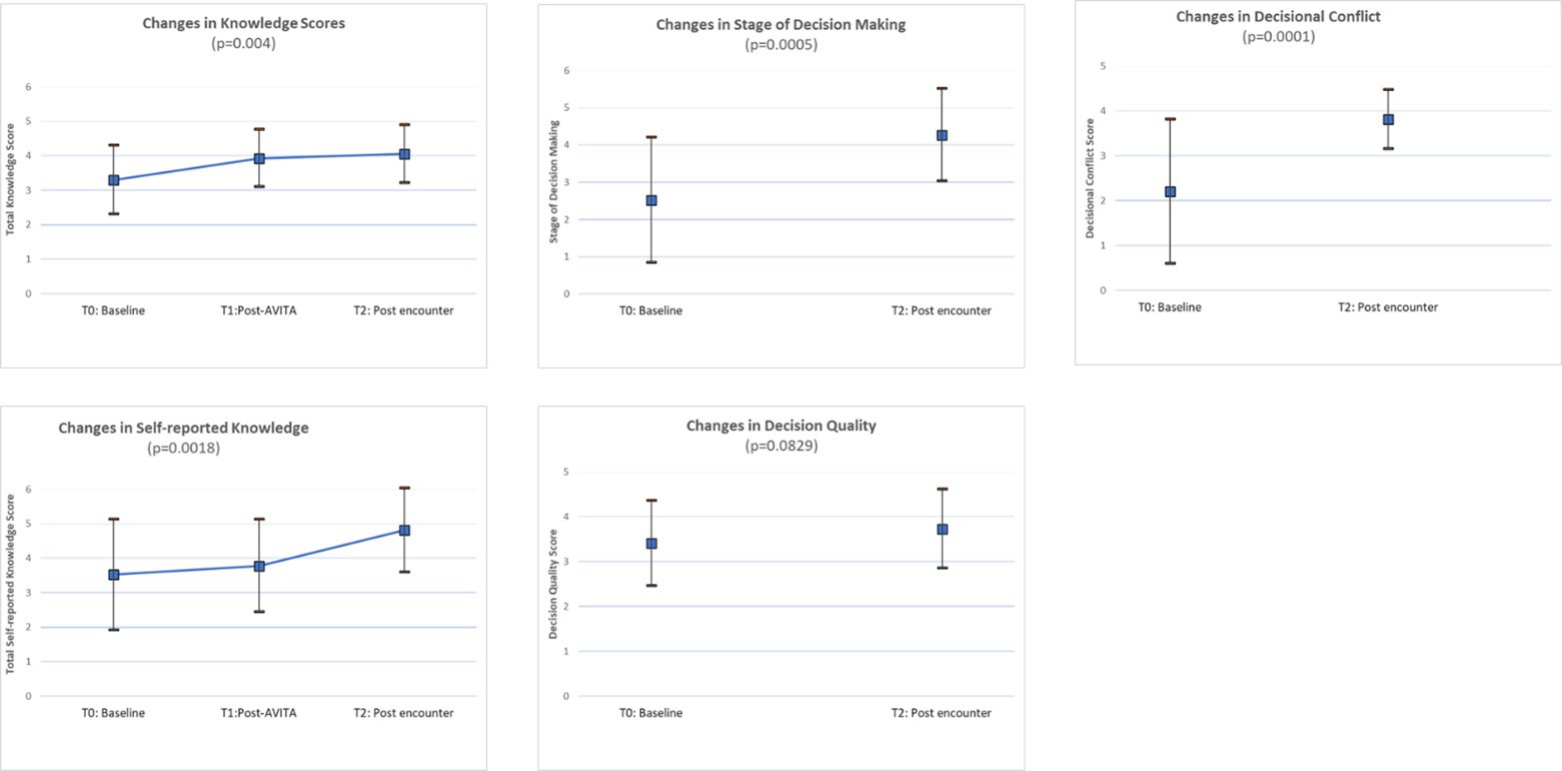
For all included outcomes, higher scores are desirable; higher decisional conflict scores reflect less decisional conflict (desirable). S2 Table in S1 File reports these longitudinal findings. Self-reported knowledge scores similarly improved from 3.52 [1.60] at T0 to 3.78 [1.34] at T1 and 4.82 (1.22) at T2 (p = 0.0018) [linear mixed-effects model]. Actual and perceived knowledge were uncorrelated at T0 (R = -0.017, p = 0.932) but strongly correlated at T2 (R = 0.507; p = 0.016). Patients’ stage of decision-making significantly improved, with more patients moving closer to deciding (mean 2.52 [1.68] at T0 vs 4.27 [1.24] at T2, p = 0.0005) [paired t-test] (Fig 3). Decisional conflict significantly improved from 2.21 [1.61] at T0 to 3.82 [0.66] at T2 (p = 0.0001) [paired t-test], corresponding to fewer people (2/22 [9.09%]) experiencing decisional conflict at T2 compared to T0 (19/29 [65.52%]. Decisional quality improved from 3.41 [0.95] at T0 to 3.73 [0.88] at T2 (p = 0.083) [paired t-test]. At T0, all patients reported that they wanted to participate at least partially in decision-making; no patient wanted to leave treatment decisions to their clinician. Clinicians accurately judged their patient’s role preference in 11/28 [39.3%] encounters, overestimated their patient’s reliance on clinician’s judgement in 11/28 [39.3%] encounters, and underestimated it in 5/28 [17.9%] (S8 Fig in S2 File). Agreement between patient-reported role preferences and clinician judgement of patients’ preferred role was low (Kappa = 0.217 [95% CI -0.078–0.512]).

Mean patient knowledge scores (range 0–5) improved from 3.31 [1.0] at T0 to 3.93 [0.83] at T1, and 4.05 [0.84] at T2 (p = 0.004) [linear mixed-effects model], where 1 point corresponds to one additional correct response (Fig 3, S2 Table in S1 File). Patient knowledge increased stepwise over time, with greater knowledge after AVITA alone, and further gains following the clinical encounter (linear slope estimate from linear mixed-effects model = 0.36, p = 0.004).

### Changes in preferred treatment

Among the 25 patients who responded at all 3 time points, most (15/25 [60%]) changed their treatment preference at least once, 5/25 [20%] changed their preference twice. Among those who initially reported having decided which treatment they wanted and being unlikely to change their mind, 4/8 [50%] changed their treatment preference, compared to 4/7 [57.1%] of those who were considering their options and 7/10 [70%] of those who hadn’t begun to think about their options. Patients’ final treatment preference was concordant with their clinician’s recommendation, with only 2 discordant (Kappa = 0.811). Nearly all patients (21/22 [95.5%]) reported trusting their clinician’s judgment.

## Discussion

This prospective, multi-center pilot cohort study found that heart teams’ use of the AVITA DA in clinical encounters with patients with sAS was feasible and improved all assessed dimensions of SDM. AVITA empowered patients to express their concerns and choose their treatment with confidence. In addition, use of AVITA at point-of-care helped clinicians engage patients in SDM and make a recommendation based on their patients’ goals and values, improving patient knowledge, stage of decision-making, decisional conflict, and decision quality.

Some previous patient preference studies and DA evaluations have relied on volunteers without the disease in question or recruited broadly from social media without confirming disease condition [37]. Given our intent to evaluate a DA’s ability to help patients prepare for the decision-making encounter and to be used by heart team clinicians to support a SDM discussion during the encounter, we selected patients who not only had sAS, but who were actively making a decision about how to treat sAS *at that encounter*. This can be a challenging encounter to capture and has limited the validity of assessing patient preferences in prior studies [38].

Feasibility of implementation of the AVITA DA is a complex and nuanced concept that encompasses patient, clinician, and diverse heart teams’ actions. Patients need to acquire sufficient knowledge to help them construct and clarify informed goals, values and preferences prior to the visit, and clinicians need to access, review, and utilize these goals and preferences at the time of SDM to arrive at a shared decision. Feasibility was thus determined by the ability of diverse heart teams to introduce the tool to patients; patients to complete the tool online; the valve coordinator to print the patient’s summary and make it available to the clinician at the time of SDM; and the clinician to review and use the summary report with the patient. Feasibility of use was further supported by patient and clinician surveys that endorsed the tool.

While many clinicians often express an abstract interest in DAs that are interactive and electronic, online and EMR-based tools have proven difficult to design, test, revise, and implement compared to simple paper-based tools that can be distributed the day of the visit. The uptake of one in five eligible patients completing the interactive, online DA, including their clinician using their informed patient values in the clinical encounter to choose treatment- has not been previously described in the literature on SDM in sAS. In addition, because learning curves are documented in the use of DAs by heart teams [29], the short duration of the current study, with less than five uses of the DA by each clinician, is unlikely to represent the extent of use over time as DAs become more integrated into workflows of heart teams.

The frequent changes in patients’ treatment choice suggest opportunities to improve decision-making. Initial treatment preferences were often uninformed (based on low knowledge), low quality (incongruent with their values), and stressful (associated with decisional conflict). After exposure to AVITA, knowledge significantly improved and treatment preferences began shifting, with final treatment preferences being more informed, higher-quality, and less stressful. That patients’ self-perceived knowledge was uncorrelated with actual knowledge at baseline, yet strongly correlated at T2, suggests that patients had difficulty assessing their own pre-intervention knowledge. Stated another way, an uninformed patient may be unable to accurately assess their certainty about their decision or the alignment between their values with their treatment choice. Exposure to the DA and their clinician changed patients’ understanding of the decision, affecting their treatment preference as well as their assessments of their perceived knowledge and other SDM constructs. Another study [39] found patient preference was the most frequent physician-reported reason for recommending medical management for sAS, yet those patients reported to have received insufficient education about treatment. Our study and others [40] demonstrate that DAs can help patients construct informed preferences, potentially leading to more appropriate and equitable care [41,42].

The uniqueness of each individual’s goals and values profile underscore the importance of assessing patient values [43]. Without direct elicitation, clinicians must rely on their assumptions about those values and risk making a “preference misdiagnosis [44].” Those assumptions are also influenced by implicit biases related to age, race, and gender [45,46]. A recent analysis of facilitators for SDM among African American patients highlight patients’ desire for more medical information and the benefit of “facilitating a level-playing field interaction [47].” Relying on patients’ initial treatment preferences without exploring their values and reasoning can perpetuate disparities in care. In our study, clinicians often *underestimated* patients’ desire to be involved in decisions, and *overestimated* the extent to which patients wanted their physician to make the decision. While other studies have also found that clinician judgments of patients’ desired role were inaccurate [48], our findings from participating clinicians are striking because patients *explicitly* shared their preferred role with their clinician via the summary.

Despite participating in a SDM study with use of a DA, clinicians still did not include all essential elements of SDM. Patients reported that half of visits did not include discussions of the risks of both TAVR and SAVR; over a third of patients were not asked what treatment they wanted; and over a quarter of visits did not mention there was more than one treatment option. Notably, all clinicians self-reported practicing SDM in every assessed visit.

Discrepancies between clinicians’ and patients’ perceptions about whether SDM occurred are informative. Misconceptions as to what constitutes SDM are widespread [49,50]. Clinicians may feel that they already involved patients in SDM by providing some patient education and may not appreciate how SDM differs from patient education and informed consent. Similarly, patients may have conflated courtesy with SDM, giving clinicians excellent communication ratings despite substantial omissions in areas that involved clinicians’ relinquishing some decision-making control to patients (e.g., awareness of options). Patients considering invasive cardiac therapies may place a higher value on kind words over informed engagement in decision-making, given the need to establish a feeling of trust with the clinician who will perform the surgery or procedure; general ratings of physician communication without specific SDM metrics may be inaccurately high.

Clinicians’ dominant reason for practicing SDM was to persuade patients to accept clinician-recommended treatment: this may point to clinician discomfort with clinical equipoise across options, or their reliance on guidelines to make recommendations without appreciating that outcomes should be valued according to what matters to the patient. Implementing SDM will require clinicians to understand what SDM entails and how patient preferences may contradict guidelines at times.

The improvements observed in patient knowledge and decisional conflict after using AVITA appear larger than those observed in another heart valve study [51], where a third of patients experienced decisional conflict after the intervention. In comparison, 65% of our participants had decisional conflict at baseline, but only 9% at T2. Because improvements at T2 reflect the combined impact of AVITA and clinicians, and AVITA targeted patients and clinicians, it is difficult to disentangle the independent effect of AVITA alone. However, outcomes that were assessed before and after the encounter (e.g., knowledge) demonstrate a stepwise improvement that began with exposure to AVITA.

Enthusiasm for promoting SDM through guidelines and policy initiatives has exceeded real-world implementation of DAs for valvular heart disease [6]. Currently, patients with sAS are recommended to consult with a heart team comprised of a cardiothoracic surgeon and an interventional cardiologist prior to treatment. Medicare patients interested in TAVR are mandated to see these two physicians to encourage a “heart team” approach via the National Coverage Determination for TAVR [52]. Physicians performing TAVR must participate in a national registry [53] that monitors whether SDM occurred (no similar requirements exist for patients referred to a cardiac surgeon for SAVR). To promulgate SDM, guidelines and policies should be accompanied by infrastructure [54], including educational programs for patients and clinicians, administrative support and guidance, and SDM tools for all patients. We and others found that DAs are helpful but not sufficient to increase SDM, even as patient knowledge, satisfaction, mental well-being [50] and other SDM measures improved [55]. Clinician skillsets and attitudes in promoting a SDM process are also essential.

Understanding the impact of SDM tools on the efficiency of clinical encounters is essential for acceptance among clinicians [56]. A frequently cited barrier to SDM is that it will take too much time [57]. A Cochrane review [41] found that DAs had a variable impact on consultation length (average 2.6 min longer). Design elements in AVITA targeting clinical efficiency—simplifying time-consuming tasks such as eliciting patient values—may explain its ability to improve efficiency in the visit as judged by physicians in 48% of AVITA-assisted clinical encounters. Improving the logistics of sharing the tool with patients and sharing the summary report with clinicians are future focus points.

### Strengths and limitations

Study strengths include testing the DA during clinical encounters in diverse real-world clinical settings and evaluating SDM broadly and from both patient and clinician perspectives. Limitations include a small sample size, which limits generalizability. However, we included representative patients and clinicians from multiple sites. We had no control group, but pre-post comparisons yielded significant and consistent findings and are appropriate when testing interventions that change patients’ frame of reference [11]. Use of an online intervention limits access to some but facilitates dissemination and updating. Self-report is subject to biases. Our recruitment methods were subject to selection bias: participants who consented may have more favorable SDM attitudes and skills. We could not assess the total number of patients invited to participate to determine precisely how frequently patients would engage with a tool such as AVITA when offered, but we estimate that it is approximately 33% of patients with internet access in this elderly population.

## Conclusions

An interactive, individualized patient-centered DA, AVITA, tested in real-world clinical encounters by heart teams, led to informed patient preferences for treatment and aligned clinicians’ recommendations with patient preferences, improved the quality of decisions, and made the clinical encounter more efficient overall. Yet even with use of the tool, gaps in SDM skillsets remained, suggesting clinician education is needed about what constitutes SDM. AVITA is now being integrated into web-based platforms. Further research on its implementation should provide further insights into the utility of this new DA, identify facilitators and barriers for real-world implementation of SDM, and generate data on patient values and preferences in the treatment of sAS across diverse populations.

## Supporting information

S1 File Tables and figures. S1 Table. Complete Data Table. S2 Table. Longitudinal Findings. S1 Fig. Patient evaluation after viewing AVITA. S2 Fig. Impact of AVITA on patient confidence in decision-making (n = 27). S3 Fig. Clinician Evaluation of AVITA after Patient Encounter. S4 Fig. Patient reported quality of care (at T2) (n = 22). S6 Fig. Clinician-reported reasons for practicing shared decision-making. S7 Fig. Contrasting clinician perspectives on patient’s preferred role in decision-making.
(DOCX)

S2 File Description of the AVITA intervention. S3 Table. Theory-based Features of AVITA. S8 Fig. Conceptual map of AVITA. S9 Fig. Screen shots from AVITA. S10 Fig. AVITA Screenshot assessing patient goals. S11 Fig. Screenshot: Framing the decision according to patient age. S12 Fig. Screenshot of a Sample Patient Summary Report. S13 Fig. Knowledge assessment questions.
(DOCX)

S3 File (DOCX)
