# Supplementary material for: An interactive, online decision aid assessing patient goals and preferences for treatment of aortic stenosis to support physician-led shared decision-making: Early feasibility pilot study
Source: PLoS One. 2024 May 21;19(5):e0302378. doi: 10.1371/journal.pone.0302378 (PMC11108138; doi:10.1371/journal.pone.0302378)
Supplement: S1 File — S1 Table. Complete Data Table. S2 Table. Longitudinal Findings. S1 Fig. Patient evaluation after viewing AVITA. S2 Fig. Impact of AVITA on patient confidence in decision-making (n = 27). S3 Fig. Clinician Evaluation of AVITA after Patient Encounter. S4 Fig. Patient reported quality of care (at T2) (n = 22). S6 Fig. Clinician-reported reasons for practicing shared decision-making. S7 Fig. Contrasting clinician perspectives on patient’s preferred role in decision-making. (DOCX) [file pone.0302378.s001.docx]

**Online Supplement 1**

**Table of Contents**

Supplement 1. Tables and Figures 2

**Table S1*.* Complete Data Table** 2

**Table S2. Longitudinal Findings** 5

**Fig S1. Patient evaluation after viewing AVITA** 6

**Fig S2. Impact of AVITA on patient confidence in decision-making (n=27)** 7

**Fig S3. Clinician Evaluation of AVITA after Patient Encounter** 8

**Fig S4. Patient reported quality of care (at T2)** (n=22) 9

**Fig S5. Patient-reported quality of clinician communication** 10

**Fig S6. Clinician-reported reasons for practicing shared decision-making** 11

**Fig S7. Contrasting clinician perspectives on patient's preferred role in decision-making** 12

# Supplement 1. Tables and Figures

### **Table S1*.* Complete Data Table**

|  | **After AVITA** | **After Clinic** |
| --- | --- | --- |
|  | **T1** | **T2** |
| **Patient participants** |  |  |
| **Patient evaluation after AVITA (% who agree, strongly or somewhat)** |  |  |
| ***Usability and content:*** |  |  |
| **It was easy to use.** | 25/28 (89.29) |  |
| **I would recommend it to others with aortic stenosis.** | 24/28 (85.71) |  |
| **I trusted the information provided.** | 27/28 (96.43) |  |
| **It contained the right amount of information.** | 22/27 (81.48) |  |
| **The personal summary reflects what matters to me.** | 26/27 (96.30) |  |
| **To what extent were your personal goals and values included in the AVITA tool? (% all or nearly all)** | 25/28 (89.28) |  |
| ***Preliminary impact:*** |  |  |
| **AVITA helped me identify my own goals and priorities regarding valve replacement.** | 24/27 (88.89) |  |
| **AVITA will help me talk to my cardiologist about what matters most to me.** | 25/27 (92.59) |  |
| **AVITA made me want to be more involved in decisions about treating my valve.** | 24/27 (88.89) |  |
| **AVITA helped me understand there are choices for treating my aortic stenosis** | 26/27 (96.30) |  |
| **AVITA helped me understand the reasons I might want to have TAVR.** | 21/27 (77.78) |  |
|  |  |  |
| **Confidence in decision making (DSE) (% yes, a lot or a little)** |  |  |
| ***Thinking about how you now manage your AS, did AVITA change how confident you feel that you can… get facts about med choices avail to you*** | 19/27 (70.37) |  |
| **Get the facts about the benefits of each choice.** | 24/27 (88.89) |  |
| **Get the facts about the risks and side effects of each choice.** | 23/27 (85.19) |  |
| **Understand the information enough to be able to make a choice** | 25/27 (92.59) |  |
| **Ask questions without feeling embarrassed** | 26/27 (96.30) |  |
| **Express your concerns about each choice** | 27/27 (100.00) |  |
| **Ask for advice** | 27/27 (100.00) |  |
| **Figure out the treatment choice that best suits you.** | 27/27 (100.00) |  |
| **Handle unwanted pressure from others in making your choice** | 25/27 (92.59) |  |
| **Let the clinic team know what's best for you** | 27/27 (100.0) |  |
| **Delay your decision if you feel you need more time** | 26/27 (96.30) |  |
| **Patient evaluations after the clinical encounter (T2)** |  |  |
| **SDM Processes (% reporting “a lot”)** |  |  |
| **Did the HCP explain/help you understand there were choices in what you could do to treat your AS?** |  | 18/22 (81.82) |
| **How much did you and the HCP talk about:** |  |  |
| **the reasons you might want to have TAVR?** |  | 11/22 (50.0) |
| **the reasons you might want to have SAVR?** |  | 8/22 (36.36) |
| **the reasons you might not want to have TAVR?** |  | 6/22 (27.27) |
| **the reasons you might not want to have SAVR?** |  | 5/22 (22.73) |
| **Did the HCP ask whether you wanted SAVR or TAVR?** |  | 14/22 (63.64) |
| **Total SDM Process score (range 0-6)** |  | 3.29 (0-5.5) |
| **SDM-Communication [CAHPS] *(% “Yes, definitely”)*** |  |  |
| ***“During your recent visit with a valve specialist, did your clinician:”*** |  |  |
| **Tell you there was more than one way to treat your condition** |  | 16/22 (72.73) |
| **Ask which treatment you thought was best** |  | 14/22 (63.64) |
| **Spend enough time with you** |  | 18/22 (81.82) |
| **Present risks and benefits of treatments** |  | 17/22 (77.27) |
| **Encourage questions** |  | 19/22 (83.36) |
| **Quality of SDM Communication (CAHPS) *(% “Yes, definitely”)*** |  |  |
| **Easy to understand** |  | 22/22 (100.00) |
| **Shows courtesy and respect** |  | 21/22 (95.45) |
| **Listens carefully** |  | 21/22 (95.45) |
| **Provides information** |  | 17/22 (77.27) |
| **General Communication Subscale (range 0-10) n=22** |  |  |
| ***How good was the clinician at:*** |  |  |
| **Using words you understand** |  | 9.55 (1.01) |
| **Looking you in the eye** |  | 9.68 (0.89) |
| **Answering all questions** |  | 9.45 (1.34) |
| **Listening to what you have to say** |  | 9.32 (1.52) |
| **Caring about you as a person** |  | 9.50 (1.37) |
| **Giving full attention** |  | 9.55 (1.37) |
| **Overall, how would you rate this doctor’s communication with you?** |  | 9.41 (1.18) |
| **Did the AVITA summary help you talk to your provider about your goals and preferences? (% yes)** |  | 21/22 [95.45] |
| **Did the AVITA questionnaire help you choose treatment for aortic stenosis? (“yes a lot” or “yes a little”)** |  | 21/22 [95.45] |
| **Did the AVITA questionnaire help you choose treatment for aortic stenosis? (“yes a lot”)** |  | 13/22 [59.09] |
| **Do you trust this clinician's judgment about your medical care?** |  | 21/21 [100.00] |
| **Clinician-reported evaluations (T2)** |  |  |
| **AVITA improved their [clinicians’] knowledge of what’s important to patient** |  | 21/26 (80.77) |
| **AVITA improved their ability to engage patient in decision making** |  | 17/26 (65.38) |
| **AVITA improved their communication with patient** |  | 21/26 (80.77) |
| **AVITA improved their ability to make a recommendation based on what’s important to the patient** |  | 16/26 (61.54) |
| **AVITA influenced their recommendations (Yes, a lot or a little)** |  | 16/28 (57.14) |
| **AVITA improved efficiency of the encounter** |  | 12/25 (48.00) |
|  |  |  |
| **Would use AVITA in future encounters^[[1]](#footnote-1)^** |  |  |
| **Yes, if part of our routine** |  | 20/26 (76.92) |
| **Yes, if the patient brings it to us** |  | 5/26 (19.23) |
| **No, it was not helpful** |  | 4/26 (15.38) |
| **No, I think routine use would be too challenging to coordinate [0]** |  | 0/26 (0) |
| **Clinician treatment recommendation** |  |  |
| **TAVR** |  | 15/22 (68.18) |
| **SAVR** |  | 5/22 (22.73) |
| **Med** |  | 2/22 (9.09) |

**Abbreviations:** AVITA, Aortic Valve Improved Treatment Approaches; CAHPS: Consumer Assessment of Healthcare Providers and Systems (CAHPS) Surgical Care Survey; DSE: Decision Self Efficacy scale; SAVR, Surgical aortic valve replacement; TAVR, Transcatheter aortic valve replacement

### **Table S2. Longitudinal Findings**

| **Variable** | **T0** | **T1** | **T2** | **p-value** |
| --- | --- | --- | --- | --- |
| **Stage of Decision-Making (Mean (SD))** | 2.52 (1.68) |  | 4.27 (1.24) | 0.0005 |
| **Having decisional conflict (SURE)** | 19/29  (65.52%) |  | 2/22  (9.09%) |  |
| **SURE score (mean (SD))** | 2.21 (1.61) |  | 3.82 (0.66) | 0.0001 |
| **Knowledge (Mean (SD)) range 0-5** | 3.31 (1.0) | 3.93 (0.83) | 4.05 (0.84) | 0.004 |
| **Self-reported knowledge (Mean (SD)) range 0-6** | 3.52 (1.60) | 3.78 (1.34) | 4.82 (1.22) | 0.0018 |
| **Decision Quality (Mean (SD)) range 0-4** | 3.41 (0.95) |  | 3.73 (0.88) | 0.0829 |
| ***My treatment plan reflects what’s important to me when I think about the pros and cons of treatment*** | 14/23  (60.87) |  | 20/23  (86.96) |  |
|  |  |  |  |  |
| **Patient treatment preference** |  |  |  |  |
| **TAVR** | 15/30 (50.00) | 16/28 (57.15) | 15/22 (68.18) |  |
| **SAVR** | 5/30 (16.67) | 4/28 (14.29) | 4/22 (18.18) |  |
| **Medical only** | 0/30 (0) | 1/28 (3.57) | 2/22 (9.09) |  |
| **Unsure** | 10/30 (33.33) | 7/28 (25.00) | 1/22 (4.54) |  |

### **Fig S1. Patient evaluation after viewing AVITA**

### **Fig S2. Impact of AVITA on patient confidence in decision-making (n=27)**

### **Fig S3. Clinician Evaluation of AVITA after Patient Encounter**

### **Fig S4. Patient reported quality of care (at T2)** (n=22)

Courteous, easy to understand

Sharing decision-making

Questions are drawn from the Patient Experience Measures from the Consumer Assessment of Healthcare Providers and Systems (CAHPS) Surgical Care Survey.

**Fig S5. Patient-reported quality of clinician communication** (N=22)

### **Fig S6. Clinician-reported reasons for practicing shared decision-making**

###

### **Fig S7. Contrasting clinician perspectives on patient's preferred role in decision-making**

(28 patient-clinician pairs)

HCP=health care provider, or clinician

1. Clinicians could select more than one option corresponding to yes. [↑](#footnote-ref-1)
