# Supplementary material for: An interactive, online decision aid assessing patient goals and preferences for treatment of aortic stenosis to support physician-led shared decision-making: Early feasibility pilot study
Source: PLoS One. 2024 May 21;19(5):e0302378. doi: 10.1371/journal.pone.0302378 (PMC11108138; doi:10.1371/journal.pone.0302378)
Supplement: S2 File — S3 Table. Theory-based Features of AVITA. S8 Fig. Conceptual map of AVITA. S9 Fig. Screen shots from AVITA. S10 Fig. AVITA Screenshot assessing patient goals. S11 Fig. Screenshot: Framing the decision according to patient age. S12 Fig. Screenshot of a Sample Patient Summary Report. S13 Fig. Knowledge assessment questions. (DOCX) [file pone.0302378.s002.docx]

**Supplement 2. Description of the AVITA Intervention**

[**Description of the AVITA Intervention** 1](#_Toc163567772)

[**Table S3. Theory-based Features of AVITA** 3](#_Toc163567773)

[**Fig S8. Conceptual map of AVITA** 4](#_Toc163567774)

[**Fig S9. Screen shots from AVITA** 5](#_Toc163567775)

[**Fig S10. AVITA Screenshot assessing patient goals** 6](#_Toc163567776)

[**Fig S11. Screenshot: Framing the decision according to patient age** 7](#_Toc163567777)

[**Fig S12. Screenshot of a Sample Patient Summary Report** 8](#_Toc163567778)

[**Fig S13. Knowledge assessment questions** 9](#_Toc163567779)

### **Description of the AVITA Intervention**

The Aortic Valve Improved Treatment Approaches (AVITA) tool is an interactive, online shared decision making (SDM) tool for people with severe aortic stenosis (sAS) and their clinicians. AVITA guides patients through a series of structured activities. The tool clarifies patient treatment goals and values, frames treatment decisions, presents treatment options, and generates a summary report that is populated with patient-reported goals and values that patients can share with their clinicians before or during the encounter. AVITA is designed for patients to use before they meet with their cardiovascular clinician/s to discuss treatment options for sAS. The summary report is designed to be used during the clinician encounter to improve patient-clinician communication.

AVITA was developed with diverse patients with sAS who had previous experience making decisions about sAS, the results of this design process are previously published.^10^ Values clarification modules assess and prioritize patients’ treatment goals and values. Separate values clarification modules assess three core preference areas: 1) Treatment goals (“What do you hope for or expect will happen after treating your AS?*”*), 2) Process goals (“What do you need to know or feel to help you choose your treatment?*”*), and 3) Treatment features (“What features or qualities of treatment are most important to you?*”*). Each values clarification module is built upon prioritized lists of goals and attributes that were derived by patients with sAS, using previously published methods.^10^ Within each of the three core preference areas, participants were asked to identify their three most important items from a list of 7-10 broad domains and then to rank order the items that they selected. For example, within the treatment goals module, participants are presented with a list of broad treatment goals (e.g., *To be independent or active*) and then asked to select the top three. Next, they are presented with a list of specific goals that conceptually map to the broad goals they selected (e.g., to be physically active), asked to choose and then rank the 3 most important items, and then allowed to add any additional items. The next 2 values clarification modules are similarly structured. The module addressing process goals includes items such as “to have a good medical team”, “To have trust in the doctor doing the procedure”, “To feel confident that I made the right decision”, and “Knowing what caused my aortic stenosis”.

Other modules assess users’ preferred decision-making role, frame the decision, address issues related to age, explore the sequential nature of decision-making for sAS, such as deciding on the type of valve (mechanical or bioprosthetic), if surgical aortic valve replacement is chosen, and willingness to accept long-term anticoagulation (Figure 2). Patient preferences for treatment (procedure, valve type) are assessed, along with their reasons for their preference. The tool generates a summary of the user’s preferences that is shown to the participant. Users then review the summary page and options for sharing the summary with their clinician. The patient’s summary is emailed to the patient and their designated clinician (or representative). The content in the summary page is framed differently for patients and clinicians, acknowledging their differing roles to facilitate SDM. The tool was created using customized Qualtrics© software.

*Identification of items included in values clarification exercises:* Cognitive mapping informed the design of the values clarification modules, using previously published methods.^42^ In brief, structured focus groups using nominal group technique (NGT) identified and prioritized patient-reported treatment goals and preferences for treatment features. Open card sorting coupled with hierarchical cluster analysis and multidimensional scaling^43-46^ were used to organize those preference items into meaningful clusters, creating a cognitive map based on how frequently items were sorted into the same category. These preference items and clusters were used to create the values clarification modules, which followed the design of a previously validated preference assessment tool.^47^ The tool was pre-tested with a small group of patients with sAS (n=7). The main change resulting from beta-testing was dividing the previously-identified large set of treatment goals into 2 smaller sets—one set that was limited to ‘process goals’ (items that did not necessarily relate specifically to the treatment options), versus the remaining items.

*General development process*: All patient-facing content was co-written and iteratively revised by people with AS and reviewed by experienced clinicians for scientific accuracy. Usability testing among patients and clinicians further guided the content, design, and tailoring algorithms, resulting in shortening of passages, removing inessential or repetitive elements, introducing skip patterns, tiering information and offering less important information as optional drill-downs, and emailing information of interest to the user upon completion. Programming bugs were identified and fixed during this testing.

*Rationale for specific design elements*: The introductory section of the tool was designed to set the emotional tone, build trust, and introduce expectations about SDM and patient-provider communication. It assesses the patient’s role preference using the Control Preferences Scale,^18^ giving tailored feedback intended to encourage a shared decision-making role. It provides tailored feedback explaining why their preferences matter (“Clinicians are experts in medicine, but you’re the only expert on you.” “Answering the following questions will help you tell your healthcare providers what matters to you. If you don't tell your healthcare providers, how will they know?”). Different graphic representations are shown to aid comprehension (Figure 2, Screen Shots from AVITA).

**Theory-based elements of AVITA**

AVITA has several unique theory-based design elements^48^ intended to catalyze patient engagement and increase receptivity to considering all options.^49^ To minimize premature elimination of options based on incompatibility with one’s values, the design of the SDM tool was informed by Image theory,^50^ a descriptive theory of how people actually make consumer decisions. Image theory has been validated in a variety of settings^9^ and has been endorsed for SDM.^8^ Image theory differs from normative decision theory, where decision-makers are modeled as considering all available options, carefully weighing each option’s benefits and harms, and betting on the option with the highest expected utility or pay-off. In contrast, image theory conceptualizes decisions as being guided by the decision-makers’ beliefs and values,^50^ choosing the course of action that helps decision-makers achieve their goals. Image theory models decision-making as a 2-step process. The first step screens-out options that are (superficially) incompatible with the decision-maker’s values and goals, with the decision-maker focusing on the negative attributes of their options. The second step involves choosing the best option from the remaining items (if any), examining their specific pros and cons. If there are no remaining options, decision-makers search for alternative options. Exploring patient goals and values before delving into details of specific treatment options is an approach to minimize premature elimination of viable options based on their perceived incompatibility with their goals and values.

### **Table S3. Theory-based Features of AVITA**

| **Theory-based recommendation^48^** | **Feature in Tool** |
| --- | --- |
| Optimize representation | All preference items and content were derived from and organized by experienced patients |
| Include all potentially appropriate options and their attributes | Relevant attributes of all options are shown to patients. |
| Suspend selection of an initially favored options (pre-selection) | Focus on goals elicitation and values--clarification prior to drill-down on options |
| Remind decision-maker of the array of values | Includes values clarification activities that require attention to the complete array of values (broad and narrow)--choosing, ranking, rating |
| Facilitate weighting of attributes | Force prioritization of most important goals and attributes (choose and rank) |

### **Fig S8. Conceptual map of AVITA**


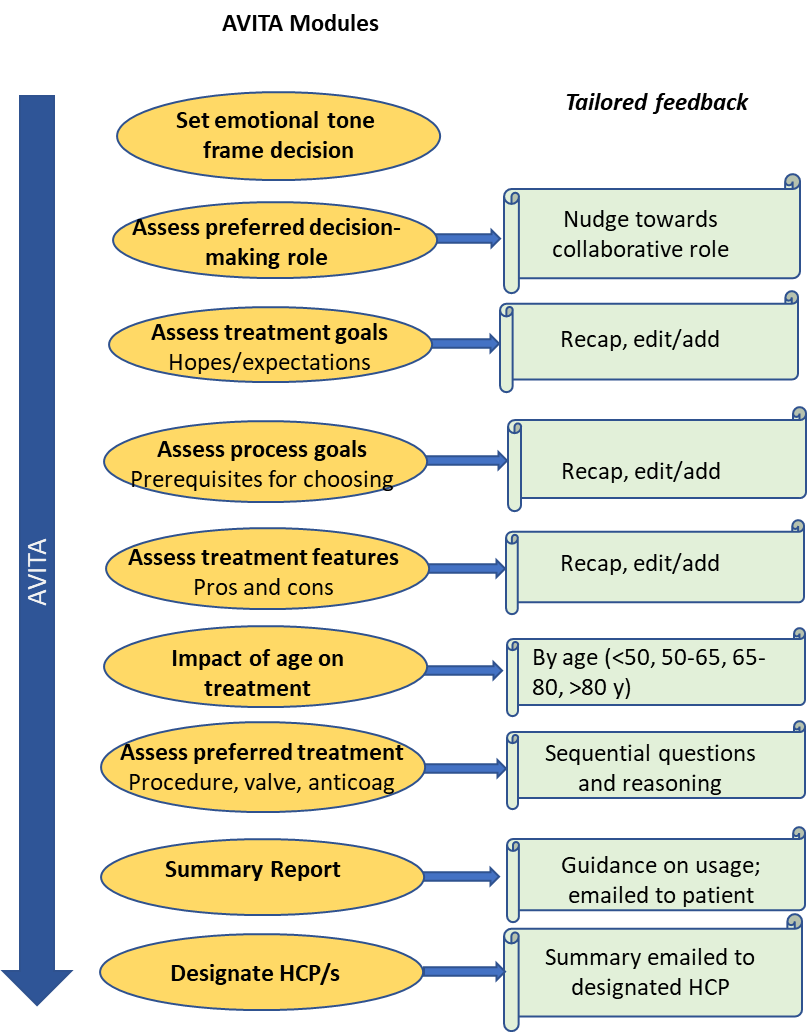


### **Fig S9. Screen shots from AVITA**

#### **Framing of the Treatment Decision in AVITA**


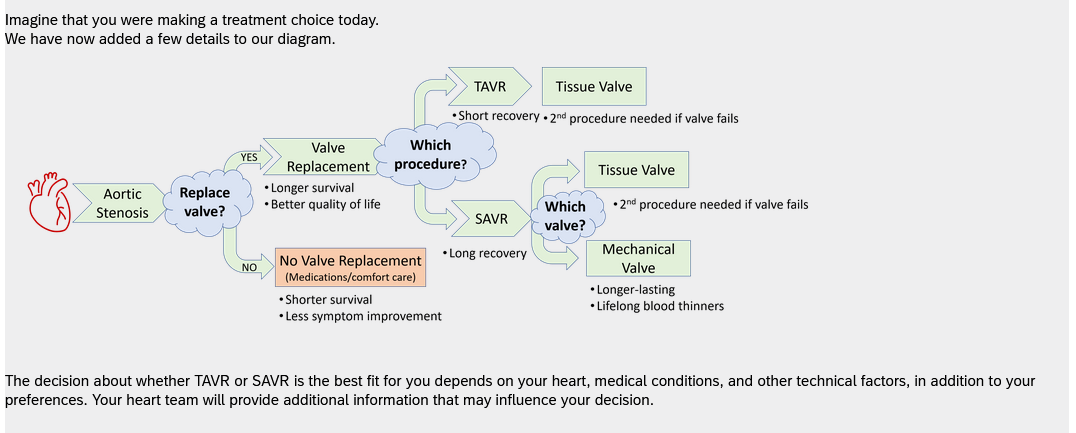


### **Fig S10. AVITA Screenshot assessing patient goals
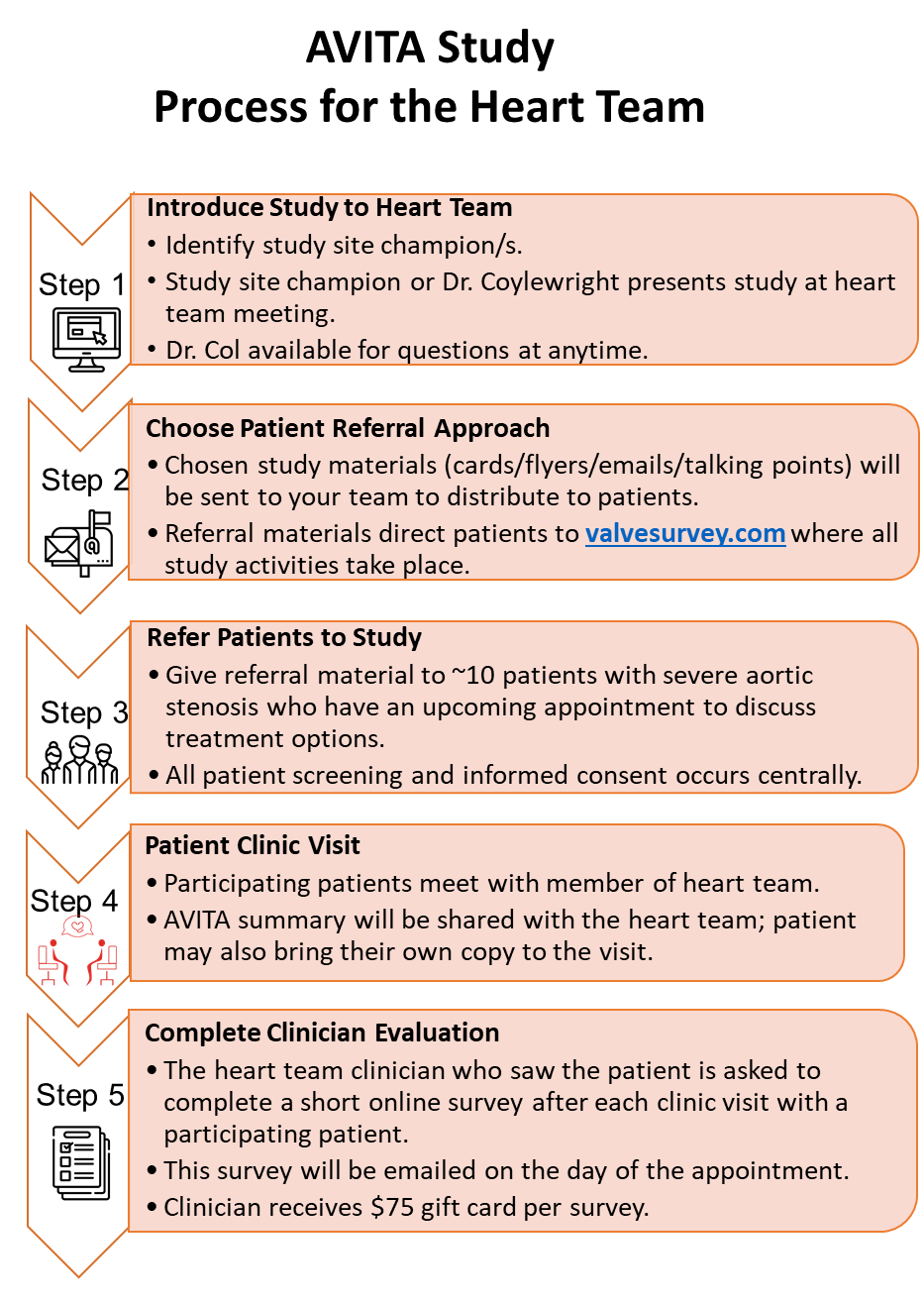
**

####
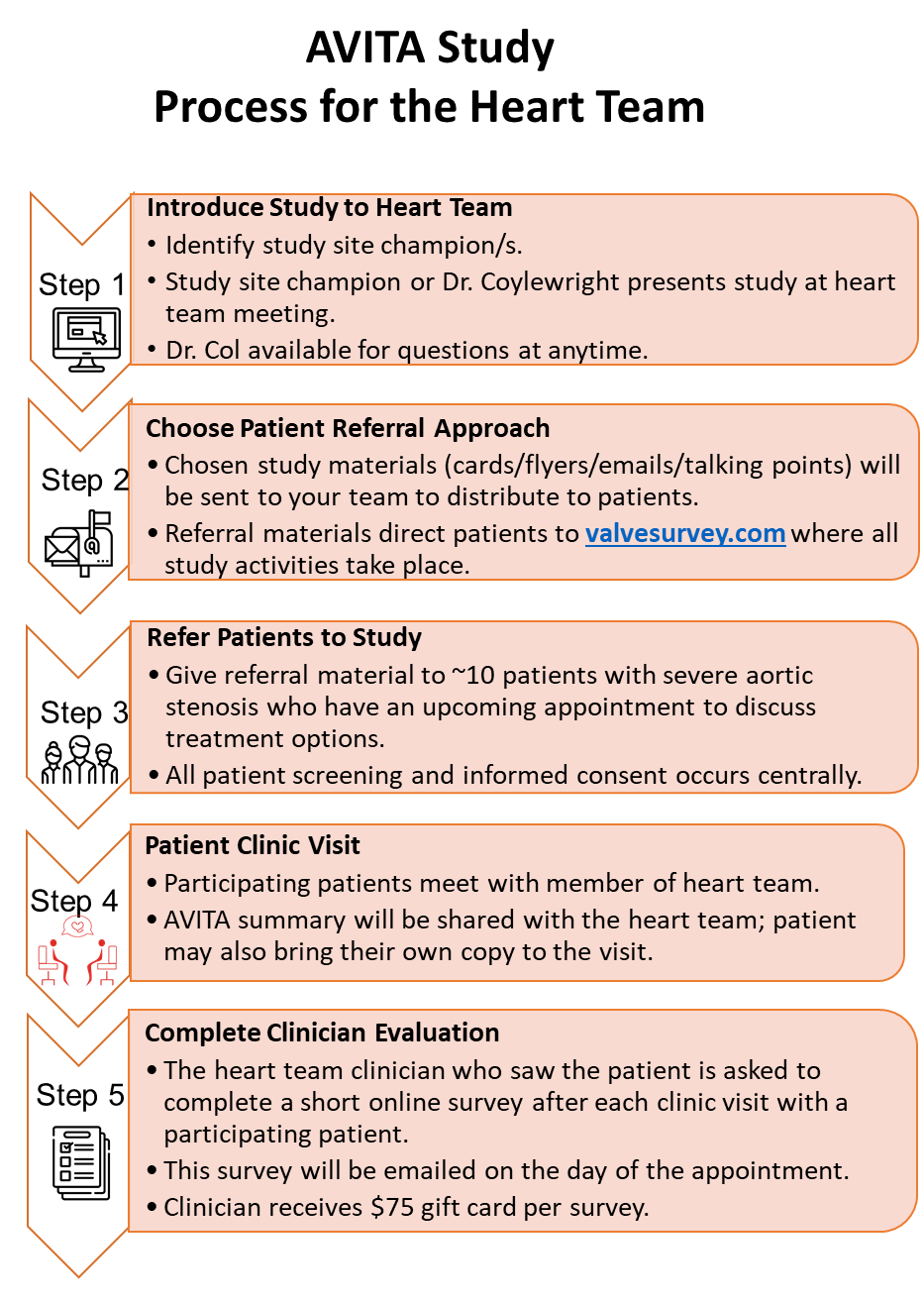


**
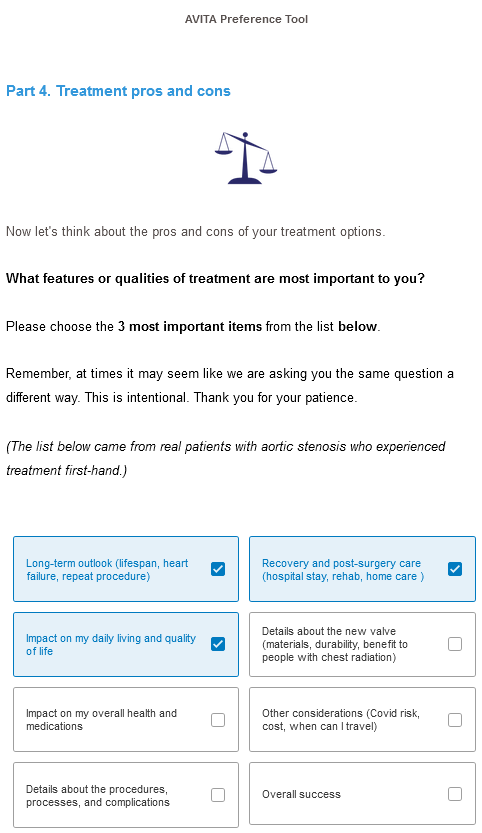
**

### **Fig S11. Screenshot: Framing the decision according to patient age**

**
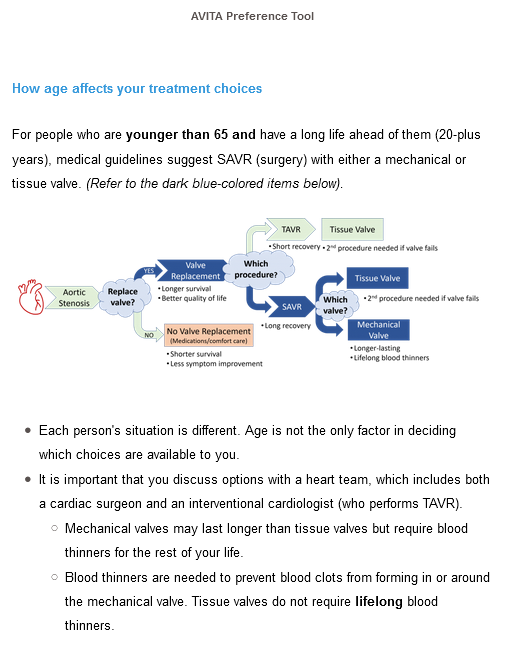
**

### **Fig S12. Screenshot of a Sample Patient Summary Report**


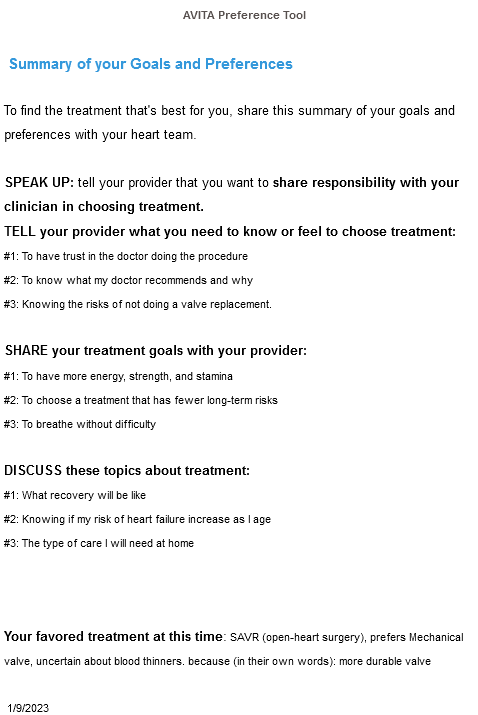


### **Fig S13. Knowledge assessment questions**

The knowledge assessment items below were adapted from the knowledge assessment items used in a previously published study (Coylewright M, O’Neill E, Sherman A, et al. The Learning Curve for Shared Decision-making in Symptomatic Aortic Stenosis. JAMA Cardiol. 2020;5(4):442–448. doi:10.1001/jamacardio.2019.5719)

**The statements below are about treating aortic stenosis. Some are true and some are false. We don't expect you to know all the answers. Please give your best guess when answering them.**

|  | **True** | **False** |
| --- | --- | --- |
| One choice is to take medicines and not have a valve replacement. |  |  |
| People are more likely to feel better with a valve replacement compared to medicines alone. |  |  |
| Medicines alone (without valve replacement) will help me live longer. |  |  |
| People who get a mechanical valve need to take blood thinners for the rest of their life. |  |  |
| Mechanical valves are just as likely to need replacement as tissue valves. |  |  |
