# Supplementary material for: An interactive, online decision aid assessing patient goals and preferences for treatment of aortic stenosis to support physician-led shared decision-making: Early feasibility pilot study
Source: PLoS One. 2024 May 21;19(5):e0302378. doi: 10.1371/journal.pone.0302378 (PMC11108138; doi:10.1371/journal.pone.0302378)
Supplement: S3 File — (DOCX) [file pone.0302378.s003.docx]

Contents

[Proposal 3](#_Toc93418850)

[Clinical Protocol Summary: 4](#_Toc93418851)

[Inclusion Criteria for Patients in the Pilot Study: 4](#_Toc93418852)

[Inclusion Criteria for HCPs in the Pilot Study 4](#_Toc93418853)

[Figure 1: Study Design and Sample 7](#_Toc93418854)

[Figure 2. Patient Recruitment Overview 10](#_Toc93418855)

[A. Specific Aims and Study Methodology 13](#_Toc93418856)

[B. Risks and Benefits 16](#_Toc93418857)

[Inclusion Criteria for Patients in the Pilot Study: 16](#_Toc93418858)

[Sources of Materials 18](#_Toc93418859)

[Data and safety monitoring plan 23](#_Toc93418860)

[Appendix A. Cognitive interviews 24](#_Toc93418861)

[Appendix B. In-person NGT Meeting Protocol 27](#_Toc93418862)

[Appendix C. Online NGT Forum 30](#_Toc93418863)

[Appendix D. Online Card Sort 57](#_Toc93418864)

[Appendix E: Screen Shots of Prototype Preference Assessment Tool 60](#_Toc93418865)

[Appendix F. Usability Testing Guide for the Preference Assessment Tool 61](#_Toc93418866)

[Appendix G: Patient Outreach and Reminder Emails (Phases 1-4) 65](#_Toc93418867)

[Email #1: Response to emails inquiring about participation 65](#_Toc93418868)

[Email #2: General Outreach 66](#_Toc93418869)

[Email #3: Targeted Outreach for Specific Activities 67](#_Toc93418870)

[Email #4: Follow-up Email about in-person NGT Group Meeting 68](#_Toc93418871)

[Email #5: Follow-up email for Online NGT Forums 69](#_Toc93418872)

[Email #6: - Pilot Study: Response to emails from patients inquiring about participation 70](#_Toc93418873)

[Email #7: Pilot Study: Patient –Invitation for T2 survey (after HCP appointment) 71](#_Toc93418874)

[Email #8: Pilot Study: Patient – Survey reminder for T0 or T2 survey 72](#_Toc93418875)

[Email #9: Pilot Study: Patient – Survey reminder (alternative) 73](#_Toc93418876)

[Email #10: Pilot Study: HCP - Survey Reminder 1 74](#_Toc93418877)

[Email #11: Pilot Study: HCP - Survey Reminder 2 75](#_Toc93418878)

[Email #12: Pilot Study: HCP - Patient/HCP Missing / incomplete data follow-up 76](#_Toc93418879)

[Appendix H. Study Outreadh—Ads, flyers, talking points 77](#_Toc93418880)

[Appendix H1: Study Flyer 77](#_Toc93418881)

[Appendix H2: Wallet-size Card 1 78](#_Toc93418882)

[Appendix H3: Wallet-size Card 2 (for untreated patients) 79](#_Toc93418883)

[Appendix H4: Pilot Study Wallet-size Card (for patients facing a decision about treatment) 80](#_Toc93418884)

[Appendix H5: Pilot Study Flyer (for patients facing a decision about treatment) 81](#_Toc93418885)

[Appendix H6: Pilot Study Talking Points (for potential patient participants) 82](#_Toc93418886)

[Appendix I: Intake Survey (Screening/baseline/consent) (Phases 1-4). 83](#_Toc93418887)

[Appendix J. Informed Consent Forms (ICF) 111](#_Toc93418888)

[Appendix J1: Cognitive Interview ICF 111](#_Toc93418889)

[Appendix J2: In-person Small group meeting ICF 114](#_Toc93418890)

[Appendix J3: Online NGT ICF 117](#_Toc93418891)

[Appendix J4: Card Sort ICF 120](#_Toc93418892)

[Appendix J5: Usability Testing ICF 122](#_Toc93418893)

[Appendix J6: Pilot Study: Patient ICF (Phase 5) 125](#_Toc93418894)

[Appendix J7: Pilot Study HCP ICF (Phase 5) 128](#_Toc93418895)

[Appendix K: Pilot Study AVITA Preference Assessment Tool (Phase 5) 132](#_Toc93418896)

[Appendix L: Pilot Study Evaluation Surveys (Phase 5) 184](#_Toc93418897)

[Appendix L1: Pilot Study T0 Intake Survey 184](#_Toc93418898)

[Appendix L2: Pilot Study T1 Post-AVITA Evaluation Survey 210](#_Toc93418899)

[Appendix L3: Pilot Study T2 Patient Survey 215](#_Toc93418900)

[Appendix L4: Pilot Study T2 HCP Survey 236](#_Toc93418901)

[Appendix M. Study Website—ValveSurvey.com (Screen shots of all pages) 253](#_Toc93418902)

[References Cited 267](#_Toc93418903)

Proposal**: Understanding Patient Goals and Preferences to Facilitate Shared Decision Making for Symptomatic Aortic Stenosis**

**Submitted** by Shared Decision Making Resources

**PI:** Nananda Col, MD, MPP, MPH, FACP; **Co-PI:** Megan Coylewright, MD

**Objectives**: **Our overall goal** is to support shared decision making (SDM) for patients with symptomatic aortic stenosis (AS). **Our objectives** are to: A) identify patient treatment goals and preferences for the attributes of treatments (henceforth referred to as “preferences”); B) develop a SDM tool to help patients with AS clarify their preferences when considering treatment options and facilitate communication of their preferences to their health care providers (HCPs), C) compare HCP perceptions of their patients’ goals and preferences to patients’ actual preferences; and D) evaluate the tool in real-world clinical settings.

**Basis/Rationale** Symptomatic AS is underrecognized and undertreated, with a one-year mortality among untreated patients at prohibitive risk for surgery approaching 50%.^[[1]](#endnote-2)^ Transcatheter aortic valve replacement (TAVR), initially approved in 2011 for patients with high to prohibitive surgical risk, provides a less invasive alternative to surgical AVR (SAVR). It was approved for intermediate risk patients in 2016, with subsequent FDA review for low risk patients^[[2]](#endnote-3),^^[[3]](#endnote-4)^ following the results of two recent randomized trials demonstrating noninferiority and superiority over SAVR.^[[4]](#endnote-5)^

The Center for Medicare and Medicaid Services (CMS) recently revised their National Coverage Determination (NCD) for TAVR (released June 21, 2019), with an amended requirement from a two-surgeon requirement for decision making to a heart team comprised of an interventional cardiologist and a cardiac surgeon. While the NCD identifies clinical expert consensus pathways’ insistence on SDM,^[[5]](#endnote-6)^ CMS stopped short of mandating SDM. They wrote that they “support patient shared decision making in AVR, but there is not a fully developed tool at this time.” Several are available, but not yet tested for efficacy (see sharedcardiology.org/tools). Recently published guidelines on the care of patients with symptomatic AS highlight the importance of “incorporation of patient preferences and values into patients’ treatment decisions” and urge clinicians not to “deviat(e) from a shared decision-making process with patients.”^6^

SDM is shown to improve decisions and outcomes,^[[6]](#endnote-7)^ yet in real-world practice patient preferences rarely guide treatment plans.^^[[7]](#endnote-8)^^ Although patient goals, values, and preferences are key elements in SDM, physicians routinely neglect to solicit this essential information.^[[8]](#endnote-9)^ Patients typically do not have pre-formed ideas about what is important to them when confronted with a new or complex situation.^^[[9]](#endnote-10)^^ Physicians make assumptions about what matters to patients, but those assumptions are often incorrect.^^[[10]](#endnote-11)^,^[[11]](#endnote-12)^,^[[12]](#endnote-13)^,^[[13]](#endnote-14)^^ Values clarification methods are often included in decision aids to help patients determine what matters to them,^4^ but typically rely on preference items selected by the developer or physicians,^7^ which may not be relevant to patients.^8^  These items often mirror the outcomes reported in clinical trials or guidelines.^^[[14]](#endnote-15)^^ Asking patients to evaluate the outcomes that are not selected by or relevant to patients lacks external validity.^^[[15]](#endnote-16)^,^[[16]](#endnote-17)^,^[[17]](#endnote-18)^^ The preference attributes that are important to patients with AS are not known.

Decision aids for patients with AS include tools used primarily outside of the clinical encounter and focus on the decision between TAVR and SAVR for intermediate or high-risk patients. Examples are the American College of Cardiology’s CardioSmart Aortic Stenosis decision aid^[[18]](#endnote-19)^ and the PCORI-funded internet-based tool, ValveAdvice.org.^[[19]](#endnote-20)^ A third tool, the paper-based *Aortic Stenosis Choice*, is designed for use during the clinic visit for prohibitive risk patients choosing between TAVR and medical management.^[[20]](#endnote-21)^ While providing valuable information about an individual’s risk and treatment options, further work is needed as none of these tools assess patient-identified preferences, include a values clarification exercise, or help patients share their preferences with their providers.

We developed and validated a novel approach to identify patient goals and preferences for treatment.^[[21]](#endnote-22)^ We incorporated those patient-derived goals and preferences into an effective preference assessment tool that helps patients understand their own treatment goals and preferences and generates an accurate summary that can be shared with clinicians. We validated that tool, demonstrating its ability to help patients clarify, summarize, and share their treatment goals and preferences with their clinician.^^[[22]](#endnote-23)^,^^[[23]](#endnote-24)^ All preference items included in these tools were generated by people living with the condition. We have honed these methods and successfully applied them to high-stakes decisions about multiple sclerosis and chronic pain. We now propose to apply these methods to decisions about AS.^21,22,23^

Clinical Protocol Summary: We propose a 5-phase mixed methods study involving nominal group technique (NGT), with patients driving the identification and categorization of preferences regarding treatment options for symptomatic AS (Figure 1). Participants include adults with a history of symptomatic AS and HCPs who guide decisions about managing AS, including interventional cardiologists, cardiac surgeons and advanced practice providers (APPs), including nurse practitioners and physician assistants.

A Stakeholder Panel will guide the project, made up of patients, HCPs and researchers, seeking input from industry, administrators, advocacy groups, family members and payers.

Inclusion criteria: The initial 4 phases of the study (generating patient goals and preferences through NGTs) will target diverse adult groups with a history of symptomatic AS who have previously made a decision regarding treatment choices. This will include patients who have undergone SAVR, TAVR, or who elected medical therapy with or without balloon valvuloplasty. The subsequent phases will include patients with AS who are currently facing a decision regarding treatment. We will recruit patients through referring clinicians located at centers selected to ensure that a) patients have a diagnosis of symptomatic AS and to b) ensure a diverse patient population with regards to gender, race, age, education, and geography. Anticipated sites include Dartmouth-Hitchcock Medical Center in NH (rural, white, low health literacy population); MedStar Washington Hospital Center (urban, population in DC 47% African American, 9% Latino); and Vanderbilt University in Nashville, TN (urban, population in Nashville 27% African American, 10% Latino), among others.

The Phase 5 **pilot study** will include both adult patients with sAS who face a treatment decision and their heart team HCP(s).

### Inclusion Criteria for Patients in the Pilot Study:

- Able to read English
- Over the age of 18
- Having a diagnosis of sAS
- Currently face a decision about treatment of sAS
- Have an upcoming appointment with a cardiology heart team HCP
- Access to the Internet and a valid email address
- Live in and receive care in the US (assessed by Zip Code).

Inclusion Criteria for HCPs in the Pilot Study*:*

- Cardiologist, cardiac surgeon, cardiology Advanced Practice Provider (APP) who manage patients with sAS.

Patient participants will be referred by their heart team HCP or potentially through patient

advocacy groups (Heart Valve Voice, American Heart Association). Referrals will target diversity with respect to representation of women, people of color, and those with low health literacy.

*Phase 1*: ***Identify and prioritize patient a) goals and b) preferences for treatment.*** The nominal group technique (NGT)^^[[24]](#endnote-25)^^ will be used to identify and prioritize patient-centered outcomes related to treatment goals and treatment features. The NGT technique consists of asking a question to the participant group and generating a list of response items. Both in-person and online NGT groups (6-8 NGT groups total), with 5-10 patients each, will be conducted to maximize the representativeness of the sample. Planning for approximately 2- 4 NGT’s per question (up to 8 total), including a minimum of 2 in-person NGTs (Lebanon, NH and Washington, DC) and the remaining conducted either in-person or online, depending on which approach better meets recruitment needs. As each NGT group consisting of 5 to 10 patients, we will aim for 56 patients (minimum of 30) in Phase 1. A separate parallel process will be done for each NGT question including a) goals and b) preferences for treatment attributes to limit patient burden for each portion of the study.

The specific wording of the NGT questions will be drafted by the PIs and research team, reviewed by stakeholders (including patient advisers) and then potentially reviewed by 1-4 patient participants during a brief modified cognitive interview. An example of the types of questions that may be asked include: “*What do you specifically hope to achieve (or avoid), now and in the future, when you choose ways to manage your aortic stenosis*?”; “*What are some of the things that run through your mind when you are deciding about which treatment option to manage your aortic valve*”. These questions were adapted from the NGT questions posed to patients with multiple sclerosis during previous application of these methods for assessing goals and treatment preferences, respectively. The protocol for the cognitive interview and NGT are described in Appendices A, B and C. We expect to be able to elicit distinct responses for treatment goals versus treatment attributes. If this is not possible, we will collapse these 2 questions into one question.

*Phase 2:* ***Card Sort****. Categorize patient a) goals and b) preferences for treatment through cognitive mapping.* Following identification and prioritization of patient-generated goals and preferences during phase 1, 25-60 patients will participate in online “card sorting” activities to organize these prioritized lists into clusters (domains). Appendix D outlines the procedures that will be used. Multidimensional Scaling (MDS) and Hierarchical Cluster Analysis (HCA) statistics will be used to analyze the data, using SPSS or SAS software.^23^ Separate cognitive maps will be developed for a) treatment goals and b) treatment attribute preferences, assuming that we are able to distinguish between treatment goals and preferences for the attributes of treatment. If not, a single cognitive map will be developed.

*Phase 3:* *Based on patient-defined goals and preferences for the treatment of AS, develop the preference assessment tool.* The patient-identified goals and preferences will serve as the core of the preference tool. We will draw from a validated SDM template^22^ to summarize and communicate patient preferences to their HCPs. An early prototype will be developed, and subsequent usability testing with patients with a history of symptomatic AS will refine the tool (n= up to 12). The outcome of this process will be a preference assessment tool to allow patients who are exploring the choice of treatment for AS for the first time to easily explore, assess and rank goals and preferences for treatment, and thus communicate them with their HCP. The prototype that will be used is available at: <https://tinyurl.com/WhatMattersMS> (screenshots are presented in Appendix E).

*Phase 4:* **Usability testing**: patients with a history of symptomatic AS will refine the tool (n= up to 12). The prototype that will be used is available at: <https://tinyurl.com/WhatMattersMS> The protocol used for usability testing of the tool is shown in Appendix F.

*Phase 5:* ***Pilot Study****:* We will pilot test the AVITA preference assessment tool with approximately 25 adult patients with sAS [range 20-40] and their heart team HCP(s). After completing online screening for eligibility, giving informed consent, and completing a baseline questionnaire (*Appendix L1: Pilot StudyT0 Intake Survey*), all patient participants will be asked to interact with the online preference assessment tool (AVITA) independently (at their chosen place and time) before the heart team appointment. AVITA generates a “snapshot” summary of their treatment goals and preferences that will be emailed or faxed to their designated heart team HCP, either directly or via their designated staff. The content of the AVITA tool is shown in Appendix K: Pilot Study AVITA Preference Assessment Tool. Just after completing AVITA, patients are asked to complete a short evaluation (including ease of use, trustworthiness, content, and helpfulness in understanding one’s goals and priorities, communicating with their cardiologist, and being involved in decision making (see *Appendix* *L2: Pilot Study T1 Post-AVITA Evaluation Survey*). After the patient’s appointment with their heart team HCP, both the patient and their heart team HCP(s) will be emailed separate evaluation surveys (see *Appendix L3: Pilot Study T2 Patient Survey* and *Appendix L4: Pilot Study T2 HCP Survey*). The HCP Pilot Survey may be faxed or mailed to the HCPs, depending on their preference.

**Primary Outcome (Pilot Study)**

Shared Decision Making will be measured using the validated patient-reported **SDM process scale.** ^[[25]](#endnote-26),^^[[26]](#endnote-27)^ Specific items in this scale are: *Did the HCP explain that there were choices in what you could do to treat your condition*?, *How much did you and the health care provider talk about the reasons you might want to have [TAVR/SAVR/Medical therapy without valve replacement]*, *How much did you and the health care provider talk about the reasons you might not want to have [TAVR/SAVR/Medical therapy without valve replacement*]; *Did the health care provider ask you whether or not you wanted to have [TAVR/SAVR/Medical therapy without valve replacement*]). Response options are: *Yes/No;* or *A lot*, *Some*, *A little*, *Not at all*, *A lot*.

**Secondary Outcomes (Pilot Study)**

- **Patient-provider communication** will be measured using: 1) the 3-item CollaboRATE Scale,^[[27]](#endnote-28)^ 2) a global communication rating (“Overall, how would you rate this doctor’s communication with you? Response options range from 0 = “the very worst I could imagine” to 10 = “the very best I could imagine”), and 3) communication about goals (“During this visit, *help you understand your health issues* with your cardiology clinician? (Y/N).
- **Treatment choice.** Options include TAVR, SAVR, or medical therapy without valve replacement. Patients’ treatment choice is assessed by self-report of their treatment preference, intent, and/or planned treatment at baseline (T0), when using the AVITA tool (T1), and after meeting with their heart team HCP (T2). Patients are also asked about their HCP’s treatment recommendation (if any) at T0.
- **Decision-making process measures** include preference for participation in decision making, decision quality, assessed as the congruence of the chosen treatment with the patient’s values (the best indicator of a high-quality decision), ^^[[28]](#endnote-29)^^ and decisional conflict using the 4-item SURE Scale. ^^[[29]](#endnote-30)^^
- **Quality of care** outcomes include items from the Consumer Assessment of Healthcare Providers and Systems (CAHPS) SDM Quality of Care Measures^^[[30]](#endnote-31)^^ that assess patients’ perception of the extent to which the treatment chosen helped them achieved their own personal treatment goals**.**
- **Knowledge,** including both subjective and objective measures to assess changes in knowledge before and after receiving the AVITA questionnaire.

**HCP outcomes** include perceptions of the value of the preference tool and the patient summary that it generates. Using previously developed instruments that include the perceived impact of the tool on the quality of care provided, efficiency of the visit, shared decision making, and patient-provider communication.^[[31]](#endnote-32)^ We also ask HCPs about their treatment recommendation (if any) and treatment selected.

### Figure 1: Study Design and Sample


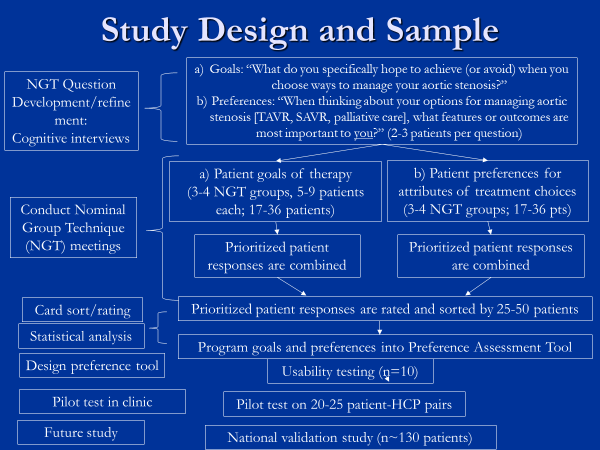


b) Patient preferences for attributes of treatment choices

(3-4 NGT groups; 17-36 pts)

Card sort/rating

Prioritized patient responses are combined

Prioritized patient responses are rated and sorted by 25-50 patients

Statistical analysis

Program goals and preferences into Preference Assessment Tool

National validation study (n~130 patients)

Future study

Design preference tool

Usability testing (n=10)

Pilot test in clinic

Pilot test on 20-25 patient-HCP pairs

**Sample size**

Because the first 3 phases of research are mixed methods involving qualitative analyses and nonparametric analyses, there is no power analysis.

**Setting**

There will be one central research site (Phases 1-5)**:**

1. The Internet

Targetted referral sites include:

Phase 1-4

- Dartmouth-Hitchcock Medical Center, Lebanon, NH, USA
- MedStar Washington Hospital Center, Washington DC, USA
- Structural Heart and Valve Center, Vanderbilt University Medical Center, Nashville, TN, USA
- University of Louisville School of Medicine, Louisville, KY, USA
- Palestine Regional Medical Center, Palestine, TX, USA
- Central Vermont Medical Center, Berlin, VT, USA
- Department of Cardiovascular Medicine, The Erlanger Heart and Lung Institute, Chattanooga, TN, USA.

**Phase 5: Pilot Study** (This list may be updated based on recruitment needs and relocations of advisory panel members)

- - Department of Cardiovascular Medicine, The Erlanger Heart and Lung Institute, Chattanooga, TN, USA.
  - Heart Valve Voice US, CA
  - Department of Cardiovascular Medicine, University of Louisville School of Medicine, Louisville, KY, USA
  - Vanderbilt University Medical Center, Nashville, TN, USA
    - Structural Heart and Valve Center
    - Department of Cardiac Surgery
  - HeartCare Specialists, Medical City North Hills. North Richland Hills, Texas, USA.
  - American Heart Association, Atlanta, GA.
  - Central Vermont Medical Center, Berlin, VT, USA.
  - Columbia University, New York, NY, USA
  - Santa Barbara Cardiovascular Medicine Group, CA
  - University of California-San Francisco, San Francisco, CA
  - HeartValve Voice

Subject Recruitment (for patients and HCPs): Phases 1-4: Recruitment will be primarily passive using purposeful and snowball sampling through strategically placed ads, flyers, cards, and emails invitations using referral channels available through Dr. Coylewright or developed by our Advisory Panel (AP) (e.g., flyers posted in physician practices). We expect to rely predominantly on HCPs referring potentially eligible patients with AS to the study through distribution of flyers, wallet-sized business cards, or emails that refer potentially interested patients to contact the study coordinator. Referring HCPs at identified sites will be contacted through physician-to-physician referrals. Recruitment channels may also include contacts at peer support groups, professional organizations, and community organizations (e.g. YMCA). A copy of the recruitment ads (flyers, and wallet-sized cards) and email invitations and outreach are shown in Appendices G (emails) and H (Study ads).

Referral HCPs or collaborating clinics or centers may place flyers in waiting rooms, bulletin boards, or other areas, distribute wallet-size cards directly to patients, or send e-mail notifications. They may mention the study at staff meetings or support group meetings or through clinician referrals. Details of subject referral will be worked out collaboratively with clinic sites.

Confirmation of AS diagnosis: We rely on patient self-report to assess whether a patient has AS, without confirmation through their medical record. Because our recruitment channels are in places in which AS patients receive medical care, this is expected to greatly increase the accuracy of self-reporting. In the pilot study, our HCP-mediated referral process coupled with patient self-report is expected to greatly increase the accuracy of self-reporting.

Incentive payments to participants: Each patient participant will receive Amazon gift card valued $50 for a 60-90 minute NGT meeting or a usability interview, $20 for the 10-20 minute cognitive interview (phone call), $35 for the online card sort survey, and $75 total for completing the **pilot study** (Phase 5), divided into 2 separate payments of $40 and $35 to encourage study retention. Refreshments will be provided at in-person NGT meetings (potentially including a meal, depending on the chosen time and location of the meeting). Some funds (up to $20) will be available in special cases (only when no free parking or public transportation is available at the chosen meeting site) to allay parking expenses*.*

HCPs who are involved during Phase 5, the pilot validation study, will receive an incentive payment of $75 for completing the short evaluation for each participating patient.

Patient Enrollment Protocol: Screening for eligibility, obtaining baseline information, and informed consent will be done centrally using the study website (see Figure 2, *Patient Recruitment Overview*). Potential participants may email the research office of Dr. Col where they will receive an invitation email containing a unique link to a confidential electronic survey (Qualtrics).

For the **pilot study**, potential participants can email the research office of Dr. Col where they will receive an invitation email containing a unique link to a confidential electronic survey (Qualtrics). They can also enroll via the study website (ValveSurvey.com) where they can learn more about the study and enroll directly by accessing a link to the confidential electronic survey (which uses Qualtrics software). The study contact email and study website URL will be listed on referral flyers and cards and also a QR code. We also include talking points for referring providers who prefer to speak directly with prospective participants ([Appendix H6: Pilot Study: Talking Points](#_Toc92893963)). The email invitation explains the purposes of the study, options for study participation, and reviews general eligibility requirements (*Appendix G, Email #6: Pilot Study: Response to emails from patients inquiring about participation).* Other survey reminder emails for patients and HCPs are also shown in *Appendix G, Emails #7-12*.

Participants will have the opportunity to opt-out by return email. Participants who do not opt-out will receive up to 3 follow-up emails and/or a telephone call.

Subjects who express an interest in participation and respond to the study invitation will click on the link to the study site and will then be asked to complete the screening for eligibility. If eligible, they will be asked to complete the baseline questionnaire. Participants in Phases 1-4 (but not the pilot study) will be asked to select which of the available study activities interest them (if more than one study activity is available). They will then be asked to give informed consent to be included in that study activity (Appendix I shows the intake Questionnaire which includes screening/baseline survey/study activity selection questionnaire, and ICF). Depending upon the phase of the study at that time (e.g., which activities are ongoing), they may be invited to proceed to the next step (e.g., take the online survey or participate in an online forum, schedule a time for the conference call), else their information and study activity preferences will be retained in the Participant Panel database so that they can be contacted at a later date about the activity that they are interested in (depending on the timing of the study activities, location of meetings, and types of subjects needed at that study phase). For the **Pilot Study**, participants who consent and complete the baseline survey will proceed directly to the next step (e.g., review the AVITA Preference Assessment Tool).

Informed consent for each study activity will be obtained online for that study activity shortly before it is scheduled (Appendix J: ICFs). Participants will be able to call or email the study PI or coordinator to address any questions they may have.

### Figure 2. Patient Recruitment Overview

For Phase 5 **Pilot Study**, referred patients can enroll in the Pilot Study via an email that includes a link to the study or via the study website (ValveSurvey.com).

**Proposed Future Directions**: Upon successful completion of Phases *1-5*, we hope to conduct a larger validation study, engaging adult patients with symptomatic AS to confirm patient preferences in a broader population, while describing the impact of a preference assessment tool on both patients and HCPs, their shared decision-making processes, the treatment chosen, and quality of care. That future study would assess the tool’s efficacy in: 1) clarifying and communicating patients’ goals and preferences to the HCP; 2) helping patients participate in SDM, 3) helping HCPs understand their patients’ preferences, and 4) helping patients achieve their own personal preferences and treatment goals, using the preference tool as a novel PRO quality measure. The primary and secondary outcomes and sample size of that future validation study will be guided by pilot study findings, in collaboration with our stakeholders.

**Significance**: This project provides innovative solutions for people with cardiovascular disease by helping patients make more informed decisions about treatment, reflecting their preferences and promoting access to TAVR as clinical indications expand. Decision aids tend to increase uptake of procedures in the setting of underuse of available therapies.^[[32]](#endnote-33)^ Recognizing that patients are more willing to undergo treatments that are less-invasive and that improve short-term quality of life, it is expected that the proposed SDM tool will result in greater penetration of treatment of symptomatic AS, addressing the current undertreatment. Our proposed tool targets all key stakeholders: while placing patients at the center of decision making and improving patient-provider communication, patient preference tools can assist HCPs to achieve higher quality ratings (which are associated with higher reimbursements) on the Consumer Assessment of Healthcare Providers and Systems (CAHPS) SDM Quality of Care Measures^^[[33]](#endnote-34)^^ and on the ACA Meaningful Use requirements.^[[34]](#endnote-35),^^[[35]](#endnote-36)^

The proposed study will be the first to identify patient-identified preferences for AS treatment, to deploy a tool to elicit individual patients’ treatment goals and preferences for AS, and to help patients with AS communicate their treatment goals and preferences with their HCPs using an SDM framework. Understanding patient preferences is essential to SDM, yet HCPs’ failure to elicit patient preferences has been identified as a key barrier to implementing SDM in clinical practice. Given that current expert consensus pathways highlight SDM as a requirement for top tier status for TAVR programs, the proposed preference assessment tool will help clinicians comply with these recommendations, simplifying the process of eliciting patient preferences and documenting that SDM occurred.^5^

Dr. Col and members of the applicant team have developed the first preference assessment tool of this kind for multiple sclerosis (MS) and chronic pain. These preference-assessment methods were recently selected by the National Quality Forum (NQF) as a Next Generation Innovator.^[[36]](#endnote-37)^ The manuscript describing the validation of the preference assessment tool for patients with Multiple Sclerosis was recently recognized as the best manuscript of the year (2018) by the Consortium of Multiple Sclerosis Centers and the International Journal of MS Care (The Herndon Award). Dr. Col recently coauthored Cochrane evidence reviews on this topic^[[37]](#endnote-38),^^[[38]](#endnote-39)^ and chaired the NIH Small Business Innovations Research (SBIR/STTR) study section on Risk, Prevention, and Health Behavior Integrated Review Group (Medical Informatics) and thus is familiar with currently available tools. The specialized proprietary software that will be used for the NGTs, sort cards, and to generate SDM templates was developed by the applicant organization for MS and chronic pain tools and is not publicly available. Previous work in MS and chronic pain established the efficacy of the software and that the online protocol was comparable to the in-person protocols.^21^

**Human Subjects Research**

## A. Specific Aims and Study Methodology

**A.1. Purpose of Research**

Decisions about treating aortic stenosis are difficult. Many patients do not know how to integrate their preferences in decisions about treatment. There are presently no validated instruments to assess patient preferences for AS treatments. Clinical guidelines highlight he importance of shared decision-making and incorporating patient preferences and values into patients’ treatment decisions for AS. This study hopes to better understand patient preferences for AS treatment in order to develop a shared decision-making tool that will help patients with AS clarify their preferences when considering treatment options and facilitate communication of their preferences to their health care providers.

**Study Design Overview:** We propose a 5-phase phase mixed methods study involving nominal group technique (NGT) and card sorting activities, with patients driving the identification and categorization of preferences regarding treatment options for symptomatic AS. We enlist an Advisory Panel and Stakeholder Design Team to provide guidance and oversight of the study.

**We propose to:**

1. **Conduct NGT meetings**: To draft the NGT question, we will work with our patient and stakeholder advisers. We will then conduct cognitive interviews to evaluate how candidate NGT questions are understood from the perspective of the patient, following a written protocol (Appendix A; n=2-6).
   1. Candidate questions describing the following preference areas include:
      1. Treatment goals: What goals drive or underlie decisions about AS treatment?
      2. Treatment features: What attributes of treatment matter most for patients?

Each of the final NGTquestions will be addressed in 3-4 NGT meetings. We propose to convene approximately 6-8 separate NGT meetings with approximately 56 (30-80) participants stratified in terms of question focus, and NGT type (in-person or online). Each meeting will consist of 5-10 subjects, last 60-90 minutes, and focus on a single issue. During each meeting, participants first will be asked to work independently and silently to generate ideas in response to one open-ended question. A round-robin approach will be used to elicit a single idea from each participant in each round. Every idea will be recorded in view of the participants and the process will continue until participants are unable to generate more ideas. At that point, the group will be invited to participate in a discussion to focus or clarify the nominated responses. Each participant will then be instructed to select 5-9 (depending on the number of responses generated by the group) of what they perceive to be the most important of the nominated responses and to rank order their selected issues. Individual rankings will be aggregated across all members of the group and the aggregated results may be presented to the group for discussion.^^[[39]](#endnote-40)^,^^[[40]](#endnote-41)^

Time permitting, we may ask the group to review the list and identify themes/categories that subsume the top ranked responses, or probe specific topics and questions, such as how treatment goals influenced their decisions in the past, how they managed trade-offs between competing goals/priorities, how they compared substantially different options, key uncertainties, timing, and sequencing of decisions. See Appendix B for details of the protocol for in-person NGT meetings, and Appendix C for details of the protocol for online NGT meetings.

1. Conduct card sorting and rating activities: We will develop separate card sorting tasks to examine how patients cognitively organize the *goals* and *treatment features* identified in the NGT meetings. For each card sorting task, participants will be provided a list of preference items (e.g., goals) and asked to consider the meaning of the phrase or word reflecting either a perceived goal or DMT feature. They will be instructed to use their own criteria (e.g., how you see these going together) to sort the items into an unspecified number (2-10) of card stacks containing any number of perceptually similar cards. The online version of this activity uses specialized software. The Card Sorting Outline of the protocol is described in Appendix D.
2. Design and program the preference assessment tool**,** based on patient-defined goals and preferences for the treatment of AS**.** The patient-identified goals and preferences will serve as the core of the preference tool. We will draw from a validated SDM template^22^ to summarize and communicate patient preferences to their HCPs. An early prototype will be developed, and subsequent usability testing with patients with a history of symptomatic AS will refine the tool. The prototype that will be used is available at: <https://tinyurl.com/WhatMattersMS>. Appendix E shows screen shots form this tool.
3. Usability testing of draft preference-assessment tool: This tool will be pre-tested among the AP and patient advisers). The panel will answer questions addressing 1) treatment goals; 2) treatment features that drive decisions about AS; 3) preference summary that is intended to be shared with their HCP. The usability testing protocol is described in Appendix F.

*Phase 5:* **Pilot test** the AVITA preference assessment tool with approximately 25 adult patients with sAS [range 20-40] and their heart team HCP(s). After completing online screening for eligibility, giving informed consent, and completing a baseline questionnaire (Appendix L1: Pilot Study T0 Intake Survey), all patient participants will be asked to interact with the online preference assessment tool (AVITA) independently (at their chosen place and time) before the heart team appointment. AVITA generates a “snapshot” summary of their treatment goals and preferences that will be emailed or faxed to their designated heart team HCP, either directly or via their designated staff. Just after completing AVITA, patients are asked to complete a short evaluation (including ease of use, trustworthiness, content, and helpfulness in understanding one’s goals and priorities, communicating with their cardiologist, and being involved in decision making (see Appendix L2: Pilot Study T1 Post-AVITA Evaluation Survey). After the patient’s appointment with their heart team HCP, both the patient and their heart team HCP(s) will be emailed separate evaluation surveys (see *Appendix L3: Pilot Study T2 Patient Survey* and

*Appendix L4: Pilot Study T2 HCP Survey*). The HCP Pilot Survey may be faxed or mailed to the HCPs, depending on their preference.

**Primary Outcome (Pilot Testing)**

Shared Decision Making will be measured using the validated patient-reported SDM process scale.^[[41]](#endnote-42),^^[[42]](#endnote-43)^ Specific items in this scale are: *Did the HCP explain that there were choices in what you could do to treat your condition*?, *How much did you and the health care provider talk about the reasons you might want to have [TAVR/SAVR/Medical therapy without valve replacement]*, *How much did you and the health care provider talk about the reasons you might not want to have [TAVR/SAVR/Medical therapy without valve replacement*]; *Did the health care provider ask you whether or not you wanted to have [TAVR/SAVR/Medical therapy without valve replacement*]). Response options are: *Yes/No;* or *A lot*, *Some*, *A little*, *Not at all*, *A lot*.

**Secondary Outcomes (Pilot Testing)**

- **Patient-provider communication** will be measured using: 1) the 3-item CollaboRATE Scale,^[[43]](#endnote-44)^ 2) a global communication rating (“Overall, how would you rate this doctor’s communication with you? Response options range from 0 = “the very worst I could imagine” to 10 = “the very best I could imagine”), and 3) communication about goals (“During this visit, did you discuss your personal goals for treatment with your cardiology clinician? (Y/N).
- **Treatment choice.** Options include TAVR, SAVR, or medical therapy without valve replacement. Patients’ treatment choice is assessed by self-report **of their treatment preference, intent, and/or planned treatment** at baseline (T0), when using the AVITA tool (T1), and after meeting with their heart team HCP (T2). Patients are also asked about their HCP’s treatment recommendation (if any) at T0.
- **Decision-making process measures** include preference for participation in decision making, congruence of the chosen treatment with the patient’s values (the best indicator of a high-quality decision), ^^[[44]](#endnote-45)^^ and decisional conflict using the 4-item SURE Scale. ^^[[45]](#endnote-46)^^
- **Quality of care** outcomes include items from the Consumer Assessment of Healthcare Providers and Systems (CAHPS) SDM Quality of Care Measures^^[[46]](#endnote-47)^^ that assess patients’ perception of the extent to which the treatment chosen helped them achieved their own personal treatment goals**.**
- **Knowledge,** including both subjective and objective measures to assess changes in knowledge before and after receiving the AVITA questionnaire.

**HCP outcomes** include perceptions of the value of the preference tool and the patient summary that it generates. Using previously developed instruments that includes the perceived impact of the tool on the quality of care provided, efficiency of the visit, shared decision making, and patient-provider communication. ^[[47]](#endnote-48)^ We will also ask HCPs about their treatment recommendation (if any) and treatment selected.

**Pilot study analyses**

Analyses will assess the feasibility of the patient referral process and study design by examining accrual rates, attrition rates, AVITA completion rates, percentage of patients who use the summary page with their HCP, survey completion rates for patients and HCPs, and feedback from participants. We will also compare responses to the various communication measures assessed at T2 to determine which, if any, measures appear to be more sensitive to assessing communication (anticipating a strong ceiling effect for responses). Evaluations of the preference tool at T1 will be descriptive (including distribution of responses, means, and standard deviations). Analyses of our primary endpoint, SDM Process scale, will include descriptive statistics for each item as well as the total score, examining rates of missing data and assessment of the distribution of responses (e.g., normal distribution). The weighting of each response is as follows: yes=1, no=0, a lot=1, some=0.5, a little=0 (based upon a published validation study).^25^ To the extent permitting given our small sample size, we will compare the correspondence between the patient’s and their HCP’s preference for treatment (using the Kappa statistic). These pair-wise analyses will be performed separately, data permitting, by type of HCP (cardiac surgeon, interventional cardiologist, and APP).

The impact on the tool on knowledge, decisional conflict, preference for participation in decision-making, treatment intent/choice, decision quality, and whether treatment goals were discussed, will be done by comparing pre and post assessments. Means (S.D.) will be compared using the chi-square for categorical values and t-tests for continuous variables. The distribution of these variables will be assessed. We do not anticipate having a large enough sample to support multivariable analyses, though simple stratified analyses will be explored (by gender, stage of decision making).

## B. Risks and Benefits

RISKS TO THE SUBJECTS

Human Subjects Involvement and Characteristics:

Our study will involve AS patients and AS HCPs to develop a shared decision making instrument for assessing patient preferences for AS treatment and communicating those preferences to their HCPs. The population will include approximately 132 patients with AS the age of 18 or over, who can read English; and 25 experienced AS HCPs (cardiologists, surgeons, PAs, and Nurse Practitioners). We encourage participants to participate in several different parts of the study, which results in some uncertainty as to the total number of unique study participants. Men and women will be included and we strive for racial and ethnic diversity. This research will not involve children because AS is a disease associated with advanced age. We intend to recruit approximately 2 patient participants for cognitive interviews, 56 patient participants for NGTs (including both in-person and online),37 patient participants for card sorting, and 25 patients and 25 HCP participants for the pilot testing.

Inclusion/Exclusion Criteria for different portions of the study:

**For AS Patients** (all study activities)**:**

*Inclusion criteria:*

- Able to read English
- Over the age of 18 (reflecting the epidemiology of sAS)
- Having a diagnosis of AS (per patient self-report)
- Currently face a decision about treatment of sAS
- Have an upcoming appointment with a cardiology heart team HCP
- Access to the Internet and a valid email address.

*Exclusion criteria:*

- Unable/unwilling to give informed consent
- Unable/unwilling to complete the AVITA Preference Assessment Tool.

### Inclusion Criteria for Patients in the Pilot Study:

- Able to read English
- Over the age of 18 (reflecting the epidemiology of sAS)
- Having a diagnosis of sAS
- Access to the Internet and a valid email address
- Currently face a decision about treatment of sAS
- Have an upcoming appointment with a cardiology heart team HCP
- Live in and receive care in the US (assessed by Zip Code).

**For HCPs** (phase 5 only):

*Inclusion criteria:*

- Cardiologist, cardiac surgeon, cardiology Advanced Practice Provider (APP) who manage patients with sAS.

*Exclusion criteria:*

- Unable/unwilling to give informed consent

All subjects involved in the study will be asked to complete a brief demographic questionnaire.. HCPs will be asked about their professional training, year of graduation, volume of AS patients that they see, and practice setting. Patients will be asked about their age gender, AS diagnosis,year of diagnosis, activity level, symptoms of heart failure, quality of life and social limitations (using the KCCQ-12^[[48]](#endnote-49)^), past treatment/s for AS, relevant comorbidities (e.g., diabetes, peripheral artery disease, atrial fibrillation, pacemaker), zip code, education and health literacy. These questions are intended to screen for eligibility and create participant panels that can be used to identify subjects that live proximal to each other to facilitate convenient in-person meetings and to identify subjects with desired characteristics (e.g., to ensure diversity of participation with regard to age, gender, race, literacy, treatments chosen, and clinical characteristics).

**During Cognitive interviews**, an experienced interviewer will read aloud and give a written copy of a brief background introduction and a potential NGT question to the subject, asking: 1) what they perceive the question is asking, 2) if the question is clear, and 3) what responses immediately come to mind. Subjects may be asked to choose amongst one or more alternative wordings of the question. Participants may be asked to “think out loud” about how they might answer the question, and suggestions on how to improve the question. Interviews last about 15-30 minutes. Responses will be qualitative. Interviews may be audio recorded (with permission) for future reference or clarification. We may also pretest and refine the online screening and baseline questionnaires during these interviews. We will pretest each question with 0-5 subjects (depending on results obtained with patient advisers on our stakeholder panel). See Appendix A for detailed Cognitive Interview protocol.

**NGT meetings** will be conducted in-person or online. Each will consist of 5-10 subjects, last 60-90 minutes total, and focus on a single issue. During in-person meetings, participants first will be asked to work independently and silently to generate ideas in response to one open-ended question. A round-robin approach will be used to elicit a single idea from each participant in each round. Embarassing or sensitive topics will be collected anonymously and read aloud by the moderator to avoid the need for participants to disclose sensitive information.

Every idea will be recorded for the group to see and the process will continue until participants are unable to generate more ideas. The group will be invited to discuss the group list, but not to criticize or otherwise evaluate, the nominated responses. Each participant will be instructed to privately select 5-9 of what they perceive to be the most important of the nominated responses and to rank or prioritize their selected issues. Individual rankings will be aggregated across all members of the group (anonymously) and the aggregated results presented to the group for discussion.^^[[49]](#endnote-50)^,^^[[50]](#endnote-51)^

The same protocol will be followed for online NGTs (participants will be asked to generate ideas based on a single question), with the exception that the list of compiled ideas, submitted online, will be shared via the private online forum (using a Qualtrics survey). Participants will then be asked to rank the ideas using this same forum (Qualtrics survey).

During card sorting, we will examine how patients cognitively organize the *goals* and *DMT features* identified in the NGT meetings. For each card sorting task, participants will be provided a list of items and asked to consider the meaning of the phrase or word reflecting either a perceived goal or treatment feature. They will be instructed to use their own criteria (e.g., how do you see these going together) to sort the cards into an unspecified number (3-10) of categories containing any number of perceptually similar cards. See Appendix D for details. This online activity will use specialized Qualtrics software.

During usability testing of the preference tool, an experienced interviewer will present participants with the tool using a shared computer screen, potentially showing alternative designs for various pages. Participants will be asked to “think out loud” about finding their way through the prototype. Interviews may be audio recorded. (See Appendix F). This testing will be done initially among our Advisory Panel, potentially enriched by their suggested contacts (patients and HCPs), as needed.

Patients will not need to undergo any specific labwork or clinical testing to participate in the study; no new clinical tests will be ordered for the purpose of this study. All data is self-reported. No clinic or medical record data will be used.

During **Pilot Testing**, online evaluations will be obtained from both patients and HCPs, addressing SDM processes, communication, SDM outcomes, treatment intention and treatment choice.

Sources of Materials**:**

The source of materials include screening and baseline data completed online, written responses to NGT questions, individual and group scoring (voting) sheets (online and paper-based), card sorting groupings (online), digital audio recordings of cognitive interviews, NGT discussions, and usability testing, and online evaluations. All of the responses obtained from NGT meetings will be anonymous (participants will write responses on blank cards that do not include their name or any other personal identifying information). Online NGT meetings (“forums”) will contain no personal identifiers. The source of material from the online surveys, including all intake and follow-up data, will be from self-administered questionnaires from both patients and their HCP. For the **pilot study**, information about which treatment is planned may be obtained through patient self-report in T2, by telephoning the patients (if they preferred that means of communication) or through the patient’s HCP or their designated staff (e.g., the HCP’s structural heart coordinator). The data will be obtained specifically for research purposes to assist in the development and refinement of our preference assessment tool and to ease its integration into clinical practice.

**Primary Data:** Phases 1-4: All study participants will complete the online screening survey followed by a baseline survey (for those who are eligible and give consent) at the time of study enrollment. These surveys include self-reported socio-demographic characteristics, employment status, education, health literacy, ability to read, write, and use a computer, year of diagnosis of AS, AS symptoms (activity, swelling, fatigue, shortness of breath, limited enjoyment of life), type of HCPs seen for AS care, past AS treatments (TAVR, SAVR, medications). All data will be self-reported. The source of material for the self-administered surveys will come largely from pre-existing, validated instruments. (see Appendix I); <https://fiveislands.iad1.qualtrics.com/jfe/form/SV_bE4Ceha7VPViD7D>

**Phase 5 Pilot Study:** All patient participants will complete the online screening survey followed by a baseline survey (for those who are eligible and give consent) at the time of study enrollment (*Appendix L1: Pilot Study T0 Intake Survey*). They will then review the AVITA Preference Assessment tool.

**Screening Data**

All study participants will complete the online screening (having a diagnosis of aortic stenosis, upcoming cardiology appointment to discuss treatment options), caregiver status Y/N)

**Baseline Data (T0)**

This survey includes self-reported patient information (name, phone, email, zip code), socio-demographic characteristics (sex, race, age, education, native language), health literacy, insurance type, year of diagnosis of AS, AS symptoms (including heart failure), baseline health (depression, anxiety, general health), comorbidities, type of HCPs seen for AS care, past AS treatments (TAVR, SAVR, medications), HCP information, patient attitudes and beliefs, role preference, Decision Quality (Values congruence), Knowledge (objective and subjective), Decisional Conflict, and Stage of Decision Making.

**AVITA Questionnaire and Evaluation (T1)**

Just after completing the preference tool ([*Appendix K: Pilot Study AVITA Preference Assessment Tool)*](#_Toc93316315), participants complete a brief evaluation of AVITA (*Appendix L2: Pilot Study T1 Post-AVITA Evaluation Survey*). This evaluation contains questions regarding usability, whether they would recommend the tool, and whether the information was trustworthy and helpful for making a treatment decision.

**Post Appointment Survey (T2)**

After their scheduled appointment with their heart team HCP to discuss treatment options, participants will be asked about their appointment (*Appendix L3: Pilot Study T2 Patient Survey*) This survey includes questions about logistics (whether the appointment occurred or was or cancelled or rescheduled, appointment type (in-person, telehealth) and HCP seen), use of and evaluation of the AVITA summary, quality of communication with their HCP, trust in their HCP, quality of care, treatment decision (preferred treatment, HCP recommendation), role preference and actual role in decision-making, decision quality (Values congruence), knowledge (objective and subjective), decision conflict, and stage of decision making.

**HCP Participant Surveys**

HCPs will be asked to complete a survey for each eligible participant they refer to the study (*Appendix L4: Pilot Study T2 HCP Survey*). HCPs will be asked about their treatment recommendation or leanings (TAVR, SAVR, if medical treatment, asked reasons why; treatment scheduled date). HCPs will be asked questions about the AVITA shared summary (If and how the patient shared the summary, if the HCP reviewed the summary, the time spent reviewing the summary, and reasons for not reviewing (if applicable). HCPs will also be asked to evaluate their experience using the AVITA questionnaire (whether they’d use again, if no: asked why; encounter communication and satisfaction ratings). HCPs will be asked about shared decision making (time spent versus expected; practiced SDM Y/N, Why Y/N, Patient preferred role). Finally, if this is the first time a HCP is completing the HCP survey, they will be asked about their training and demographics (gender, present position, specialty center Y/N, years practicing).

**Potential Risks**

Potential risks are of four types: 1) breach of confidentiality resulting in loss of privacy, 2) time, cost, and inconvenience of emails, teleconference (Zoom) calls, in-person meetings, and survey completion, 3) coercion from compensation, 4) content of the discussion: participants could experience psychological stress or anxiety resulting from thinking about AS and its treatment.

2. Adequacy of Protection against Risks

a. Recruitment and Informed Consent

Screening for eligibility and obtaining baseline information and informed consent will be done online (centrally) using the study website. People interested in participating will email or call the research office of Dr. Col. Those who indicate interest who are identified as potentially eligible will then receive an invitation email that will contain a unique link to the study URL and the phone number and email of the PI to contact about any questions. The email will explain the purpose of the study, options for study participation and why they have been selected to participate. Participants will have the opportunity to opt-out by return email.

Participants will be able to click on the link to the study site and then complete the screening for eligibility online. Participants who do not opt-out and who do not complete the screening and/or baseline questionnaire may receive up to 3 follow-up emails and a telephone call.

If the subject meets inclusion criteria, they will be asked to select which of the available study activities are of interest to them and asked to give informed consent to be included in that activity (Appendix I), and then asked to complete the baseline questionnaire. Depending upon the phase of the study at that time (e.g., which activities are ongoing), they may be invited to proceed directly to begin the activity (e.g., begin online card sorting or online NGT), else be asked for further information to schedule the activity (e.g., for an in-person NGT or teleconference for usability testing). Preferences for future activities will be retained in the Participant Panel database so that they can be contacted about opportunities to participate as they arise (depending on the timing of the study activities, location of meetings, and types of subjects needed at that study phase). Informed consent for each study activity will be obtained for that study activity shortly before it is begun or scheduled. Participants will be able to call or email the study PI to address any questions they may have, or to opt out.

During in-person activities, participants will be reminded that they had previously consented online and offered an opportunity to ask any questions. Participants will be reminded of the need for confidentiality. No consenting will be performed at the study activity nor in any clinic sites. This is intended to decrease the burden on subjects by decreasing the total duration of the meeting. All participants will have the opportunity to opt-out from the study at any time. Copies of the consent form will be available.

The informed consent forms (Appendix J) contain detailed information on the study protocol, description of topics to be discussed, the patient’s right to decline participation at any time, and that declining participation will not affect standard of care. The informed consent document discloses the following risks of participation to patients: losing privacy, feeling upset from thinking about AS and its treatment, and the inconvenience of participating. The informed consent documents have been reviewed by patient stakeholders to ensure that they are easy to understand by our target audience.

Patients who contact us about the study but decline participation will be recorded using only aggregate de-identified information that will be available only to study personnel.

b. Protection Against Risk

The alternative to participating in this study is to not participate. Study participation is strictly voluntary and participants can withdraw at any time. Participation, refusal or discontinuation at any time will involve no penalty or loss of benefits to which patients are entitled under standard care.

1. Protection against Breach of confidentiality and violation of privacy.

Patient identifying information will be collected at recruitment through the screening and baseline survey and will be necessary for patient contact and follow-up. We have extensive procedures in place to minimize the risk of violation of subjects’ confidentiality and privacy, including physical and electronic methods of protecting data. Every patient will be assigned a unique study identification (ID) number, and the file used for data analysis will contain only this study ID number and no identifying information such as names or addresses. Only those persons directly involved in patient care, recruitment and follow-up will be privy to patient identity. Links between study ID and patient identifiers will be kept separately, any electronic files will be password-protected, and only the PI will have access to these files. No attempt will be made to identify an individual in the analytic file. Any paper records will be kept under lock and key unless in active use. All results will be aggregated and no research or study report will allow the identification of individual participants.

Any paper forms (including notes from NGT meetings) will be locked in a secure filing cabinet at all times, and only authorized study personnel will have access to these forms. Names will be removed from any pages and replaced by study ID. Names will be kept separately in a locked or password-protected file. Data will be kept on secured servers (Qualtrics) or on electronically password-protected files. Only study personnel who have completed the mandatory Human Subjects research training and HIPAA training will be allowed access to this data. Identifiable data will be used only to link records across databases when necessary. Access to summarized data prior to publication will be limited to study personnel.

To minimize the risk that comments made during interviews or group discussions will result in loss of privacy, participants will be asked not to repeat comments beyond the discussion and to only use first names during the discussions. Audio recordings and transcripts will be stripped of spoken HIPPAA identifiers (names, dates, location). Audio recordings will be stored in a password-protected file until they are either transcribed or deleted.

We use only Qualtrics highly secure software to collect and store electronic data and to transmit emails containing the study links. This software has numerous features to protect confidentiality. Qualtrics' servers are protected by high-end firewall systems, and vulnerability scans are performed regularly. Complete penetration tests are performed yearly. Qualtrics uses Transport Layer Security (TLS) encryption (HTTPS) for all transmitted data. Surveys are protected with passwords and HTTP referrer checking. Data are hosted by third party data centers that are SSAE-16 SOC II certified; all data at rest are encrypted, and data on deprecated hard drives are destroyed by U.S. DOD methods and delivered to a third-party data destruction service. Data will be transferred using encrypted storage devices (e.g., thumb drive).

2) Time, cost, and inconvenience of email, telephone and survey completion:

Every effort will be made to make surveys as brief as possible in order to minimize participant burden while maintaining the integrity of research goals. Surveys are self-administered at the convenience of participants. We estimate the baseline questionnaire will take no longer than 10 minutes to complete, and the NGT online forum 60-90 minutes to complete. Card sort activities should take less than 30 minutes.

3) Coercion from compensation:

For Phases 1-4, the gift cards valued at $20-50for patients are modest enough so as not to coerce participation in the study, and standard of care will not be affected by study participation. For the **Pilot Study**, Amazon gift cards with a total value of $75 for patients (divided into 2 payments ($40 and $35) to encourage retention), are modest enough so as not to coerce participation. HCPs receive a single gift card of $75 for completing the HCP survey.

Light refreshments will be provided and some funds (up to $20) may be made available to allay parking expenses for some of the in-person study activities*.* Subjects will be informed that there are no other financial benefits from participation. In addition, patients will be told that participation is voluntary, that consent can be withdrawn at any time, and that participation does not affect clinical care.

4) Content of the discussion and/or preference assessment tool (for the Pilot Study).

Information about AS and its treatment is already broadly available. Any information we present to patients will be based upon the best available scientific evidence about treatment options and risks and will be carefully reviewed by leading experts in the field and undergo extensive pretesting to ensure that the content presented is understood as intended. We believe that these discussions present no greater than minimal risk and have developed a plan to reduce these risks.

Data and safety monitoring plan: Not applicable.

INCLUSION OF WOMEN AND MINORITIES

Women and minorities will be recruited in the proportion in which they meet inclusion criteria and provide consent.

INCLUSION OF CHILDREN

Children will not be included in the study because symptomatic AS is not a disease of children and the communication strategies for children with AS would necessarily be very different and designed to target children and their parents or caregivers, which is not the purpose of this study.

## Appendix A. Cognitive interviews

After obtaining informed consent, participants with AS will be asked to talk with us on the telephone using video-conference software (Zoom.com) in which the investigator’s computer screen can be shared. The discussion will last 15 to 30 minutes and may be audio recorded. If the meeting is in person, the participant will meet with study staff in a private meeting room at a nearby hotel or designated facility.

Participants will be shown a draft question along with a short introduction to the question and asked what they think the question is asking, how they might answer it, and how they might make the question easier to understand and answer. The question will be designed to ask about what's most important when making decisions about their health, healthcare, or choosing the best treatment for AS. We may discuss sensitive topics such as AS symptoms or personal values that come into play when making a health-related decision. If time permits, we may ask the participant to critique another question.

**Cognitive Interview Protocol**: Note that all Video Conferences will use Zoom Software where the participant is viewing the investigator’s computer screen on their home computer or portable device.

**Discussion script (guide):**

Good (morning/evening/afternoon) and welcome. Thank you for taking the time to be part of this study. My name is [Nananda Col], and I am the [Lead Researcher/ Study Coordinator/Research Assistant].

Before we begin, let me suggest some things that will make our discussion more productive. With your permission, we will be recording the session because we don’t want to miss any of your comments. We will destroy the recordings after we have transcribed them or completed the study. We will not use your full name or any personal identifiers in the transcripts.

We are interested in your opinion on how we ask questions about Aortic Stenosis. These questions, once finalized, will be presented to small groups of 5-10 people with AS, and they will be asked to write down responses to those questions.

I will read to you the question. You can read along using the written version that you can see on my computer screen. You can take notes as you like. We will then ask you some questions. We would like your candid point of view. Do not worry about saying anything that might hurt my feelings. If something about the question makes you uncomfortable, anxious, or confused, please let me know so that we can improve the question. We encourage you to share any and all of your thoughts and reactions to the materials we present. You can choose not to answer the questions discussed if you do not feel comfortable.

My role here is to ask questions and listen.

Any questions before we proceed?

I wanted to remind you that we will be recording this meeting. Is that OK with you?

(If yes, proceed; if no, then turn off recording option on ZOOM)

This is a cognitive interview about AS decisions focusing on [treatment goals, treatment decisions] at [LOCATION], led by [PRIMARY FACILITATOR] on [DATE].

INTRODUCTION

Today we’d like to hear from you about what you think about a question regarding your AS and choosing a treatment. We realize that some of these issues can be personal, and we’ve tried to be mindful of this in the phrasing and scope of the questions. If you are willing to talk but prefer that we turn off the recording device, that’s fine too.

As I read you the background to the question and the question, I would like you to first think about whether the question is clear, and what you think it is asking.

I am now going to read it to you, including the introductory paragraph. [I have emailed the question to you and you can also see it on my computer screen.] You can read along and take notes, marking anything that is confusing or upsetting.

[Everyone has their own experience with AS. What’s important to one person may not be important to another. There are different ways to manage AS. These include valve replacement, less invasive procedures, and medical therapy. These approaches have differing benefits, risks, and costs. In addition to reducing the symptoms resulting from aortic stenosis, there are many other things that may matter to you when you think about managing your aortic stenosis. These could be things you hope to accomplish or things you hope to avoid. You can think of them as treatment goals. That’s why we’d like to learn about your goals and what’s important to you in your life when you make decisions about managing AS. Here’s the question I’d like you to think about. Because you’ve already made a decision about treating AS, we’d like you to reflect on how you made your decision about treatment, and also on what would be your goals if you were making that decision today.

**What do you specifically hope to accomplish (or avoid), now and in the future, when choosing how you manage your Aortic Stenosis?]**

Please **silently** write down your **main** treatment **goals** using brief phrases or short sentences.

***************************************************************************

1) What do you think this question is asking?

2) Is the question clear?

3) What responses immediately come to mind? Write down as many answers as you can [on the paper.]

4) Was there anything upsetting or confusing about this question?

5) How might you make it better?

6) Here is another way of asking this question: [insert alternate wording, if developed]

7) What about the background information given?

- Was it enough? Too much?
- What there any information you think we should have included? Removed?
- [insert alternate wording of quesion, if available]

8) Which wording do you prefer?

9) Do you have any other suggestions for how we might improve either the **introduction** or the **question** itself?

10) Are there any other things that we haven’t included that would have helped you answer this question?

11) Do you have any other comments/ suggestions that you would like to share?

12) Would you be willing to comment on a refined version of the question at a later time?

Concluding Remarks: Thank you so very much for participating in our interview. We’ve learned a lot from you, and your comments will be invaluable to our research project. I will be here for the next few minutes if you have any questions about the study. You can also e-mail us your questions.

## Appendix B. In-person NGT Meeting Protocol

Each of the final NGT questions will be addressed in NGT meetings with the target audience (patients with AS). Each meeting will consist of 5-10 subjects, last 60-90 minutes, and focus on a single NGT question. During each meeting, participants first will be asked to work independently and silently to generate ideas in response to one open-ended NGT question. A round-robin approach will be used to elicit a single idea from each participant in each round. Every idea will be recorded in view of the participants and the process will continue until participants are unable to generate more ideas. At that point, the group will be invited to participate in a discussion to focus or clarify responses, but not to criticize or otherwise evaluate, the nominated responses. Each participant then will be instructed to select 5-9 (depending on the number of responses generated by the group) of what they perceive to be the most important of the nominated responses and to rank or prioritize their selected issues. Individual rankings will be aggregated across all members of the group.^^[[51]](#endnote-52)^,^[[52]](#endnote-53)^^ If time permits, the aggregated results will be presented to the group for discussion.

We expect that each panel of NGT participants will generate between 24 to 36 unique responses to the question that is being addressed with approximately 80% overlap in substantive content across panels addressing the same issues. The final refined lists will each consist of approximately 20-25 non-redundant responses that convey similar meanings across the two groups.

**Detailed protocol**: During NGT meetings, the following steps will be implemented:

A series of short explanatory videos may be used to standardize the procedures across NGT groups.

1. **Introduction**:

An experienced moderator (and trained assistant) will deliver a welcome statement, facilitate brief introductions, review session structure, and present relevant background material to set the context for the question. Clarification questions will be sought and answered. Remind participants that consent was done online but briefly review the importance of keeping everything said in the room confidential. Illustrate the desired level of abstraction and scope with an example which does not distort (lead) group responses (e.g., choosing a vacation locale).

1. **Silent generation of ideas** in writing:

An experienced moderator will: 1) present the nominal question to the group in writing and read the question out loud 2) ask the group to write ideas in brief phrases or statements (on a card/sheet of paper) 3) ask group members to work silently and independently 4) sanction disruption of silence and encourage independent activity by comments addressed to the group as a whole.

1. **Round robin** recording of ideas using a projected computer screen or a flip chart:
2. Each participant provides an idea (one of their responses to the question) in turn. No discussion occurs, although the leader may ask for a show of hands on how many participants had a similar idea. Those responding then eliminate that idea from their respective lists. The process may continue in a circular fashion until all participants’ lists are exhausted.
3. Explain the need to present ideas in brief words or phrases, that the process will take one idea serially from each member, that group members must decide if items are duplicates, that an individual may 'pass' when he/she has no further items, but may 're-enter' later, express the desirability of ‘hitch-hiking’ and adding new ideas even if they are not on individual’s NGT list/worksheets, explain the inappropriateness of discussion prior to completion of listing, record ideas as rapidly as possible, record ideas in the words used by group members, provide assistance in abbreviating only in special situations (e.g., AS means aortic stenosis), make the entire list visible to all, sanction group as a whole if individuals engage in side conversations or attempt to discuss items prior to completing the listing.
4. **Serial discussion** to clarify ideas listed during the round robin:
5. Verbally define the purpose of the step:
   1. To clarify the meaning of items
   2. To explain reasons for agreement or disagreement
6. Indicate that final judgements will be expressed by voting, so arguments are unnecessary
7. Pace the group so that all ideas receive sufficient time for clarification
8. Avoid forcing the member who originally lists the idea to be solely responsible for clarifying the item.
9. Vote on item importance
   - 1. Ask the group to individually select from the entire list a specific number (7+/-2) of priority (important) items^^[[53]](#footnote-1)^^
     2. Participants write each priority item on a separate 3x5 card (see figure below) or rating form
     3. Rank order or rate the selected priority items
     4. Collect the cards or rating forms and shuffle them to retain anonymity. **
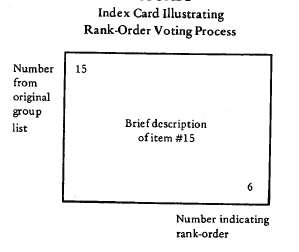
**

Depending on the remaining time, the session may end here. If time remains, continue:

- - 1. Tally the vote and record the results on the computer spread sheet (or flip chart) in front of the group. A format similar to that shown below will be used:

**
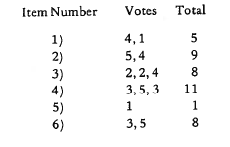
**

The following 2 steps are optional and will depend on the time remaining.

1. Discuss the vote
   - - 1. Define the role of the step as clarification, not pressure towards artificial consensus
       2. Keep the discussion brief
       3. Caution group members to think carefully about any changes they make in their voting
2. Final vote
   - - 1. Repeat step 5.

Short Break: Time permitting, we may:

1. Ask the group to review the list and identify themes/categories that subsume the top ranked responses
2. We may probe specific topics and questions, such as how treatment goals influenced their decisions in the past, how they compared substantially different options, key uncertainties, timing, and sequencing of decisions.

## Appendix C. Online NGT Forum

We developed a 2-step online NGT protocol that mirrored the in-person protocol. Participants can respond when and where they chose (asynchronously), spend as much time as needed, and complete the activity over multiple sessions. Instructional videos (identical to those shown during the in-person meetings) explain procedures. In step one, participants will view the videos, read the NGT question, and submit their responses. After all participants’ responses are submitted, responses will be consolidated independently by two facilitators (NFC and CF/VS), involving a third advisor to resolve any differences. In step two, 1-3 weeks later, participants will be shown the consolidated list, ask if they agree with the way items were combined and how they would like to change it. They will then be asked to rate each item for clarity (“is this concept clear?”, “how would you suggest it be improved?”) and redundancy (is the item unique? If not, “which other item(s) should it be combined with?”), and then asked to rank the top 9 items.

After obtaining online informed consent,

**Part one of the Online NGT Survey:**

1. Introduction: Participants will view the welcome statement, instructions for the exercise, review of session structure, and read an illustration of the desired level of abstraction and scope using an example.
   1. Opportunity to request clarification from the moderator (via a personal message or email to the facilitator).
   2. View relevant background material and NGTquestion online, including reminder to work independently; give time frame for responding (8-48 hours); and the opportunity to request clarification from the moderator.
2. Participants type ideas in brief phrases using the online survey.
3. Participants will be informed that they may be contacted for questions and that part two will be ready within a couple of weeks.
4. Email reminders to non-responders to respond (up to 3 emails over the time period)
5. All responses are automatically sent to the moderator (via Qualtrics survey).
6. Clarification with panel members about any confusing items (e.g., typos, unable to make sense of the term).

**Interim analysis conducted by investigators**:

1. The moderator and one study team member independently combine items, using a similarly formatted page ('unique' responses in left column, right colulmn showing items representing similar concepts).
2. They work together to resolve disagreements, using a 3rd or 4^th^ team member to resolve differences.
3. The final document produced is generated, showing the original items and the final consolidated items

**Part two of the Online NGT Survey:**

1. Introduction: Welcome and instructions for next step are shown as a short video. Instructions include review of the consolidated list, noting areas of agreement or disagreement, and assessing the clarity of the items. The goal is to simplify the list and remove redundancies.
2. Participants are asked to weigh in on uncertain judgements about what's unique vs not.
3. Obtain agreement from all members on the final list of unique, non-overlapping items.
4. If adequate agreement on the composition of the list is attained, then ask each person to select from the entire list a specific number (7+/- 2) of priority (important) items^^[[54]](#footnote-2)^^
5. Rank order that selected priority items (online)
6. If adequate agreement on the composition of the list is not attained, then the feedback obtained about the specific items is reviewed, the composite list is reworked, and this step is repeated. Note that in previous online NGTs, this additional step was not needed.

The online survey that will be adapted to AS (after the NGT question is framed) is shown below:

ONLINE NGT Part 1: Treatment features

Start of Block: NGT Consent

Q1
Thank you for your interest in being part of an online forum as part of the ASPIRE Study.  
 To learn more about this, please read the **Consent Form for Participation in a Research Study**.
   **[INSERT ICF HERE]**

- Yes, I agree (1)
- No, I do not agree (2)

Display This Question:

If Thank you for your interest in being part of an online forum as part of the MS Decisions Study.  ... = No, I do not agree

Q2 Thank you for taking the time to learn about our study. Feel free to contact us at a future time if you are interested in participating in other study activities.

Skip To: End of Survey If Thank you for taking the time to learn about our study. Feel free to contact us at a future time...() Is Displayed

Q4  
**Hello and welcome! Thank you for being part of the ASSIST Study. Please watch the following video to learn more about the study. If you like, you can also read the transcript of the video**.
 
 [VIDEO HERE]
 
*Thank you for watching.  Would you also like to read a transcript of the video?*

- Yes (1)
- No (2)

Display This Question:

If   Hello and welcome! Thank you for being part of the MS

Decisions Study. Please watch the followi... = Yes

Q5 Transcript of Video #1:

Hello and welcome! I would like to thank you for being part of the ASPIRE Study. My name is Nananda Col and I am leading the study. Our objective is an important one. At the conclusion of this study, we will have a better understanding of what’s important to people with AS and their health care providers when they think about treatment options.

Q7
Please watch the next video:

[INSERT VIDEO HERE]

*Would you also like to read a transcript of the video?*

- Yes (1)
- No (2)

Display This Question:

If Please watch the next video: Would you also like to read a transcript of the video?     = Yes

Q8 Transcript of Video #2:

You and 5 to 9 other people are participating in this online forum. Our success depends on every member contributing their insight from his or her own perspective. There are no status differences amongst us in this forum. I appreciate the willingness of every one of you to fully contribute your ideas. The ideas which you generate during this time will become the basis of a preference assessment tool.

Q10

To understand what will happen, please watch the next video.
[INSERT VIDEO HERE]
 
*Would you also like to read a transcript of the video?*

- Yes (1)
- No (2)

Display This Question:

Q11 Transcript of Video #3:

Here’s what will happen. I will ask you a question that I want you to think hard about and write down your responses. The question will not be about how much you know, but about what’s important to you.  If you don’t understand the question, you can ask me to explain it by sending me an online message. After each of you has written down all of your responses, we will combine everyone’s answers (anonymously) onto one list, remove any duplicates, and review the items to make sure that everyone understands them. Then you will privately vote on the items that are most important to you. I’ll combine everyone’s votes and give you the group ranking.   Remember that there is no topic that is too messy to write down. We want you to be frank. We understand that there may be some topics that are sensitive or embarrassing. But those topics may be important, and we want to know about them. No one else in the group will know who came up with the sensitive or embarrassing topics, but those topics will be included on our group list for consideration.   I will now illustrate the process using an example. Let’s say the question is “**What’s important to you when you choose your next winter vacation destination?**” Please write down your responses using a brief phrase or a few words.
 Responses could include things like “visiting my family”, “downhill skiing”, ‘outdoor adventure’, ‘inexpensive', ‘not having to fly’, and ‘warm weather’. I’m sure you can each think of other things. You might wonder how general or specific your answer should be. Well, that depends on what’s important to you. Let’s think about weather. Many people seek ‘good weather’ but that means different things to different people, right? Some people may only care that it’s warm, but a skier might be looking for cold weather and fresh snow. If someone really didn’t have anything specific in mind but the idea of good weather was still very important to them, then good weather would be what that person would write. They are all good answers, and each conveys a different meaning. We are looking for the level of detail that is important to **you**.

 We expect that it will be hard to answer the question, so we will give you enough time to think about your answers.

Here's the question we'd like you to answer: [INSERT NGT QUESTION HERE]

Please be as specific as you can. Please list as many aspects that you consider important, using brief phrases or short sentences.

- 1 ________________________________________________
- 2 ________________________________________________
- 3 ________________________________________________
- 4 ________________________________________________
- 5 ________________________________________________
- 6 ________________________________________________
- 7 ________________________________________________
- 8 ________________________________________________
- 9 ________________________________________________
- 10 ________________________________________________
- 11 ________________________________________________
- 12 ________________________________________________
- 13 ________________________________________________
- 14 ________________________________________________
- 15 ________________________________________________
- 16 ________________________________________________
- 17 ________________________________________________
- 18 ________________________________________________
- 19 ________________________________________________
- 20 ________________________________________________

Display This Question:

If display < 2

Q16 Would you like to enter more responses?

- Yes
- No

End of Block: NGT FORM Question

Start of Block: End of Part 1 Intermission

Q17 Thank you. We will now take a break while we compile the answers from the others in the group. We'll get back to you by email as soon as we are ready to resume. It may take a week or two. We thank you for your patience.

End of Block: End of Part 1 Intermission

**END OF ONLINE NGT PART 1.**

***********************

**PART 2 begins below**  *It will be given about a week later, allowing study team to compile findings and adapt survey below to include the complied responses from Part 1.*

Online NGT _Part 2

Q1 Welcome back to our Online Forum. 
    Since you last heard from us, members of our research team have reviewed everyone's responses to the question "**[INSERT NGT QUESTION HERE"**   We have reviewed all of your responses and combined similar responses.  The left column below shows the **Final Voting Response List**. The right column shows the **Similar Response List,** which are responses that seemed to be saying the same thing as the response on the left. These responses are worded as they were written by group participants.    

[SHOW COMBINED LIST OF ITEMS. Below are actual responses from a group addressing MS treatment goals]


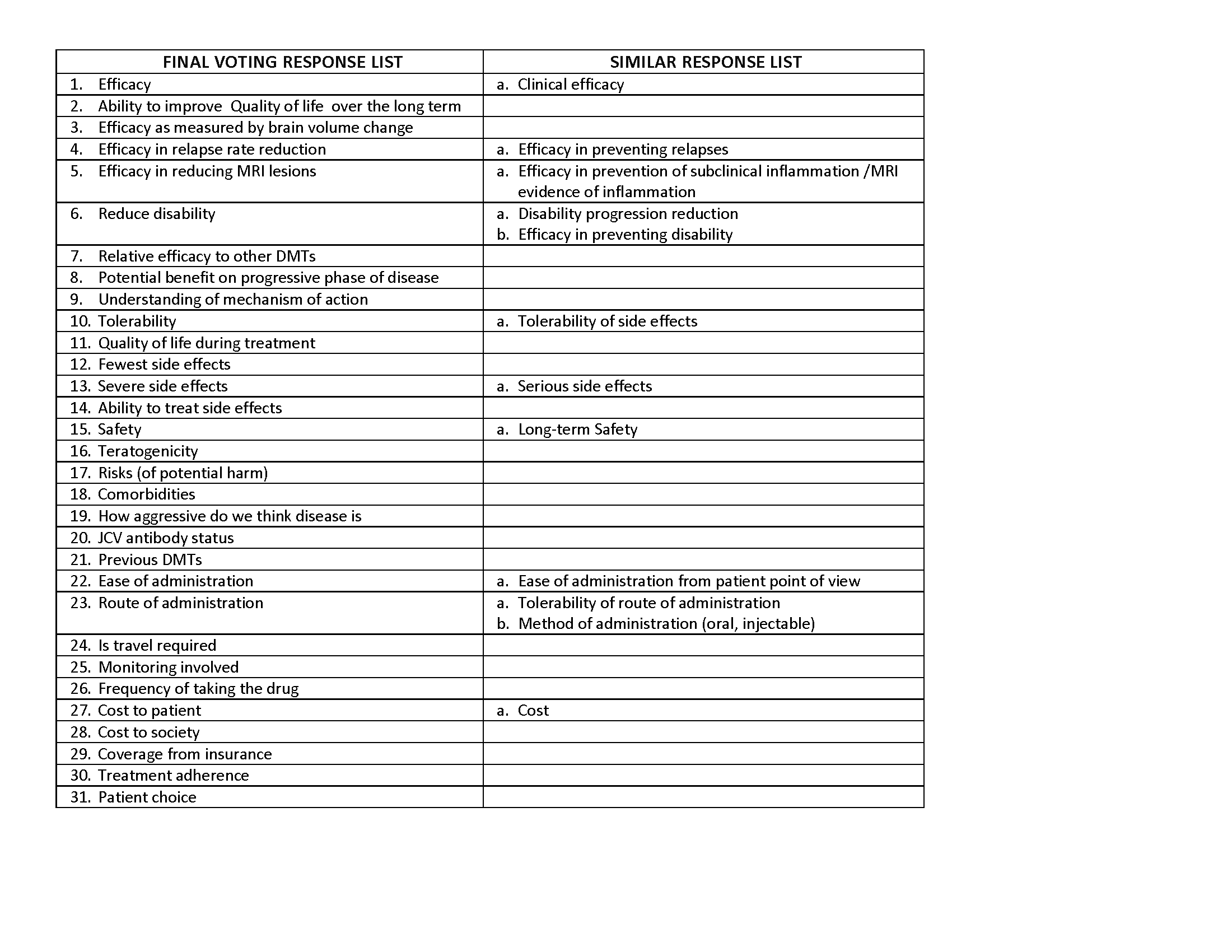


*Please think about how these responses are combined and if you think that the Final Response List makes sense.*     *What do you think about this list?*

- I am OK with the way items are combined on the list (1)
- I would like to change the way some of these items are grouped (2)

Display This Question:

If Welcome back to our Online Forum.      Since you last heard from us, members of our research team... = I would like to change the way some of these items are grouped

Q2 Which item or items do you think it is important to change, and how? Please refer to the question number.
 
You can go back by using the red arrows to navigate backwards and forwards through the survey

________________________________________________________________

________________________________________________________________

________________________________________________________________

________________________________________________________________

________________________________________________________________

| Page Break |  |
| --- | --- |

Q3 Below is the list of items generated by the group. We would like to see if the items are clear. Please read each item, and indicate whether it is clear to you. If an item is unclear, please write how you might improve it.

|  | If this item is not clear, how would you suggest it be improved? | Is this concept clear? | |
| --- | --- | --- | --- |
|  | Answer (1) | Yes (1) | No (2) |
| 1. Efficacy |  |  |  |
| 2. Ability to improve Quality of life over the long term |  |  |  |
| 3. Efficacy as measured by brain volume change |  |  |  |
| 4. Efficacy in relapse rate reduction |  |  |  |
| 5. Efficacy in reducing MRI lesions |  |  |  |
| 6. Reduce disability |  |  |  |
| 7. Relative efficacy to other DMTs |  |  |  |
| 8. Potential benefit on progressive phase of disease |  |  |  |
| 9. Understanding of mechanism of action |  |  |  |
| 10. Tolerability |  |  |  |
| 11. Quality of life during treatment |  |  |  |
| 12. Fewest side effects |  |  |  |
| 13. Severe side effects |  |  |  |
| 14. Ability to treat side effects |  |  |  |
| 15. Safety |  |  |  |
| 16. Teratogenicity |  |  |  |
| 17. Risks (of potential harm) |  |  |  |
| 18. Comorbidities |  |  |  |
| 19. How aggressive do we think disease is |  |  |  |
| 20. JCV antibody status |  |  |  |
| 21. Previous DMTs |  |  |  |
| 22. Ease of administration |  |  |  |
| 23. Route of administration |  |  |  |
| 24. Is travel required |  |  |  |
| 25. Monitoring involved |  |  |  |
| 26. Frequency of taking the drug |  |  |  |
| 27. Cost to patient |  |  |  |
| 28. Cost to society |  |  |  |
| 29. Coverage from insurance |  |  |  |
| 30. Treatment adherence |  |  |  |
| 31. Patient choice |  |  |  |

Q4 We would now like to know if you think any items should be combined or removed. Please read through all items on the list,  then respond to the questions next to each item. If you think that some of the items are saying the same thing, please write down the number(s) of the items that you think should be combined. You only need to write down **the number** of the repetitive statement once in the answer column.

|  | If this item is not unique, which other item(s) should it be combined with? Please write the statement number(s) | This item is unique | |
| --- | --- | --- | --- |
|  | Answer (1) | Yes (1) | No, it is the same as another item (2) |
| 1. Efficacy (58) |  |  |  |
| 2. Ability to improve Quality of life over the long term (59) |  |  |  |
| 3. Efficacy as measured by brain volume change (60) |  |  |  |
| 4. Efficacy in relapse rate reduction (61) |  |  |  |
| 5. Efficacy in reducing MRI lesions (62) |  |  |  |
| 6. Reduce disability (63) |  |  |  |
| 7. Relative efficacy to other DMTs (64) |  |  |  |
| 8. Potential benefit on progressive phase of disease (65) |  |  |  |
| 9. Understanding of mechanism of action (66) |  |  |  |
| 10. Tolerability (67) |  |  |  |
| 11. Quality of life during treatment (68) |  |  |  |
| 12. Fewest side effects (69) |  |  |  |
| 13. Severe side effects (70) |  |  |  |
| 14. Ability to treat side effects |  |  |  |
| 15. Safety |  |  |  |
| 16. Teratogenicity |  |  |  |
| 17. Risks (of potential harm) |  |  |  |
| 18. Comorbidities |  |  |  |
| 19. How aggressive do we think disease is |  |  |  |
| 20. JCV antibody status |  |  |  |
| 21. Previous DMTs |  |  |  |
| 22. Ease of administration |  |  |  |
| 23. Route of administration |  |  |  |
| 24. Is travel required |  |  |  |
| 25. Monitoring involved |  |  |  |
| 26. Frequency of taking the drug |  |  |  |
| 27. Cost to patient |  |  |  |
| 28. Cost to society |  |  |  |
| 29. Coverage from insurance |  |  |  |
| 30. Treatment adherence |  |  |  |
| 31. Patient choice |  |  |  |

Q5


If you wrote that some of the items are unclear or the same as another item, a member of the research team may contact you by e-mail to further clarify this item.

 Are there any other aspects that you would like to add to the list?

- Yes
- No

Display This Question:

If If you wrote that some of the items are unclear or the same as another item, a member of the rese... = Yes

Q6 What would you like to add?

________________________________________________________________

________________________________________________________________

________________________________________________________________

________________________________________________________________

________________________________________________________________

End of Block: Grouping and eliminating redundancies

Q7 Please review all of the items on the final list (shown below) and rank them from #1 (most important) to # 9. This will require careful thought on your part. Please take your time.   First, decide which one is the most important to you.  Type a #1 next to that item. From the remaining items, identify that which is most important to you and type #2 next to it. Continue until you have typed #1 through #9 into your top 9 choices. **Not all items will be ranked, only your top 9 choices.**   Note that even though some of the items may have seemed unclear or overlapping, we ask that you rank the items as best you can.

Once you select your top 9 items, you **will not be able to come back** to this page. You **will** be able to re-order your rankings on the next page.

______ Efficacy

______ Ability to improve Quality of life over the long term

______ Efficacy as measured by brain volume change

______ Efficacy in relapse rate reduction

______ Efficacy in reducing MRI lesions

______ Reduce disability

______ Relative efficacy to other DMTs

______ Potential benefit on progressive phase of disease

______ Understanding of mechanism of action

______ Tolerability

______ Quality of life during treatment

______ Fewest side effects

______ Severe side effects

______ Ability to treat side effects

______ Safety (60)

______ Teratogenicity

______ Risks (of potential harm)

______ Comorbidities

______ How aggressive do we think disease is

______ JCV antibody status

______ Previous DMTs

______ Ease of administration

______ Route of administration

______ Is travel required

______ Monitoring involved

______ Frequency of taking the drug

______ Cost to patient

______ Cost to society

______ Coverage from insurance

______ Treatment adherence

______ Patient choice

Q8 Please review your choices below. If you would like to change the ranking of the items you selected, use your mouse to click and drag the items into a different order.

Please review all of the items on the final list (shown below) and rank them from #1 (most import... [ Efficacy ] > 0

______ Efficacy

Please review all of the items on the final list (shown below) and rank them from #1 (most import... [ Ability to improve Quality of life over the long term ] > 0

______ Ability to improve Quality of life over the long term

Please review all of the items on the final list (shown below) and rank them from #1 (most import... [ Efficacy as measured by brain volume change ] > 0

______ Efficacy as measured by brain volume change

Please review all of the items on the final list (shown below) and rank the from #1 (most import... [ Efficacy in relapse rate reduction ] > 0

______ Efficacy in relapse rate reduction

Please review all of the items on the final list (shown below) and rank them from #1 (most import... [ Efficacy in reducing MRI lesions ] > 0

______ Efficacy in reducing MRI lesions

Please review all of the items on the final list (shown below) and rank them from #1 (most import... [ Reduce disability ] > 0

______ Reduce disability

Please review all of the items on the final list (shown below) and rank them from #1 (most import... [ Relative efficacy to other DMTs ] > 0

______ Relative efficacy to other DMTs

Please review all of the items on the final list (shown below) and rank them from #1 (most import... [ Potential benefit on progressive phase of disease ] > 0

______ Potential benefit on progressive phase of disease

Please review all of the items on the final list (shown below) and rank them from #1 (most import... [ Understanding of mechanism of action ] > 0

______ Understanding of mechanism of action

Please review all of the items on the final list (shown below) and rank them from #1 (most import... [ Tolerability ] > 0

______ Tolerability

Please review all of the items on the final list (shown below) and rank them from #1 (most import... [ Quality of life during treatment ] > 0

______ Quality of life during treatment

Please review all of the items on the final list (shown below) and rank them from #1 (most import... [ Fewest side effects ] > 0

______ Fewest side effects

Please review all of the items on the final list (shown below) and rank them from #1 (most import... [ Severe side effects ] > 0

______ Severe side effects

Please review all of the items on the final list (shown below) and rank them from #1 (most import... [ Ability to treat side effects ] > 0

______ Ability to treat side effects

Please review all of the items on the final list (shown below) and rank them from #1 (most import... [ Safety ] > 0

______ Safety

Please review all of the items on the final list (shown below) and rank them from #1 (most import... [ Teratogenicity ] > 0

______ Teratogenicity

Please review all of the items on the final list (shown below) and rank them from #1 (most import... [ Risks (of potential harm) ] > 0

______ Risks (of potential harm)

Please review all of the items on the final list (shown below) and rank them from #1 (most import... [ Comorbidities ] > 0

______ Comorbidities

Please review all of the items on the final list (shown below) and rank them from #1 (most import... [ How aggressive do we think disease is ] > 0

______ How aggressive do we think disease is

Please review all of the items on the final list (shown below) and rank them from #1 (most import... [ JCV antibody status ] > 0

______ JCV antibody status

Please review all of the items on the final list (shown below) and rank them from #1 (most import... [ Previous DMTs ] > 0

______ Previous DMTs

the final list (shown below) and rank them from #1 (most import... [ Ease of administration ] > 0

______ Ease of administration

Please review all of the items on the final list (shown below) and rank them from #1 (most import... [ Route of administration ] > 0

______ Route of administration

Please review all of the items on the final list (shown below) and rank them from #1 (most import... [ Is travel required ] > 0

______ Is travel required?

Please review all of the items on the final list (shown below) and rank them from #1 (most import... [ Monitoring involved ] > 0

______ Monitoring involved

Please review all of the items on the final list (shown below) and rank them from #1 (most import... [ Frequency of taking the drug ] > 0

______ Frequency of taking the drug

Please review all of the items on the final list (shown below) and rank them from #1 (most import... [ Cost to patient ] > 0

______ Cost to patient

Please review all of the items on the final list (shown below) and rank them from #1 (most import... [ Cost to society ] > 0

______ Cost to society

Please review all of the items on the final list (shown below) and rank them from #1 (most import... [ Coverage from insurance ] > 0

______ Coverage from insurance

Please review all of the items on the final list (shown below) and rank them from #1 (most import... [ Treatment adherence ] > 0

______ Treatment adherence

Please review all of the items on the final list (shown below) and rank them from #1 (most import... [ Patient choice ] > 0

______ Patient choice

| Page Break |  |
| --- | --- |

Q40 When you are happy with your rankings, please click here

- I am done

| Page Break |  |
| --- | --- |

Q9 Thank you. As soon as we have everyone's responses and ratings, we will email you the results. Depending on everyone's  responses, we may ask you to do a second and final rating.  Thank you for your help - we will get back to you soon.

End of Block: rank check java

## Appendix D. Online Card Sort

Q1 To help us better understand the similarities between the responses listed on the previous page, we now ask you to sort these items into groups.

Instructions:

1.     Read through all of the items before you begin sorting.

2.     Based on your own perceptions and experience please **sort these items into groups based on how similar they are to each other**.  Use whatever rules make sense to you to decide how these items should be sorted.  There is no right or wrong way to sort them.

**IMPORTANT:**

1)    Try to sort the items into groups that make sense to you.
 2)     Sort the items that you think are similar in the same group and sort those that you think are different into different groups.
 3)     Each group should have at least 2 items.
 4)     You should have 3 or more groups**.** **5)     You do not need to use all of the boxes that are provided.** 6)   If there is an item that does not fit into any of your groups, please place that item in the box labeled UNMATCHED ITEMS. Please limit the unmatched items to 3.
 7)     This will be difficult. Please take all the time you need.

| 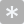 |
| --- |

Q15 **Below are responses from patients when asked [what do you hope to achieve (or avoid) when managing the patient's AS.]**  
 **Click and drag each item into a group.  The order and numbering of the item within each group does not matter.**

| Group 1 | Group 2 | Group 3 | Group 4 | Group 5 | Group 6 | Group 7 | Group 8 | Group 9 | UNMATCHED ITEMS-limit 3 |
| --- | --- | --- | --- | --- | --- | --- | --- | --- | --- |
|  |  |  |  |  |  |  |  |  |  |
|  |  |  |  |  |  |  |  |  |  |
|  |  |  |  |  |  |  |  |  |  |
|  |  |  |  |  |  |  |  |  |  |
|  |  |  |  |  |  |  |  |  |  |
|  |  |  |  |  |  |  |  |  |  |
|  |  |  |  |  |  |  |  |  |  |
|  |  |  |  |  |  |  |  |  |  |
|  |  |  |  |  |  |  |  |  |  |
|  |  |  |  |  |  |  |  |  |  |
|  |  |  |  |  |  |  |  |  |  |
|  |  |  |  |  |  |  |  |  |  |

Q15 What label or description would give to your groups? You can go back to review your groups by scrolling up. If you cannot think of a label for a Group, please skip to the next group. Please do the best you can. Leave blank any groups that you did not use.

- Group 1 ________________________________________________
- Group 2 ________________________________________________
- Group 3 ________________________________________________
- Group 4 ________________________________________________
- Group 5 ________________________________________________
- Group 6 ________________________________________________
- Group 7 ________________________________________________
- Group 8 ________________________________________________
- Group 9 ________________________________________________

| Page Break |  |
| --- | --- |

Q12 How confident were you about being able to complete this activity?

- Very confident that I did it correctly
- Somewhat confident that I did it correctly
- Not very confident

Q18 Did you have enough boxes to sort your responses?

- Yes
- No

Q19 How difficult was this process?

- Very Difficult
- Difficult
- Moderately Difficult
- Somewhat Easy
- Easy

Q17 Thank you for taking the time to be part of our study. Your responses are very important to us and we greatly appreciate the time that you have spent.

## Appendix E: Screen Shots of Prototype Preference Assessment Tool


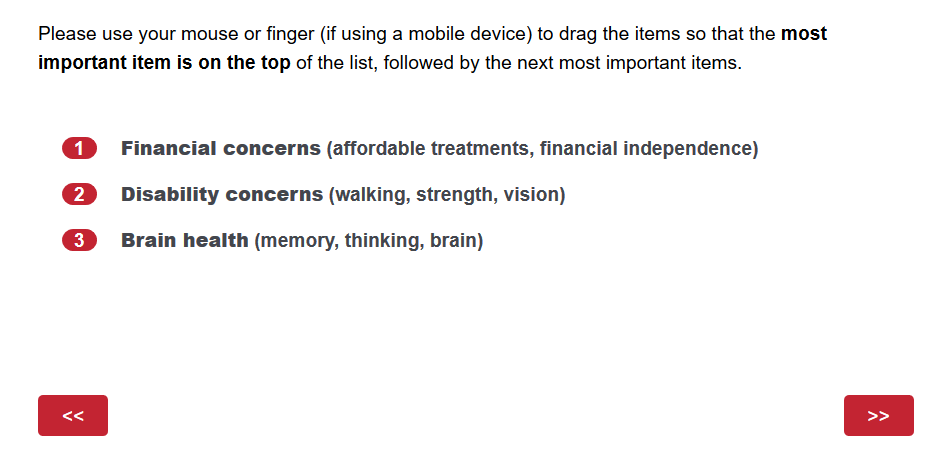


## Appendix F. Usability Testing Guide for the Preference Assessment Tool

Outline

Interview summary

Interview details

Introduction (Informed consent, Study procedures, etc)

Part 1: Unguided usage

Part 2: Guided discussion

Part 3: Information-seeking tasks

Part 4: HCP summary pages

Part 5: *Evaluation* Questionnaires

Part 7: Debriefing

TELECONFERENCE INTERVIEW SUMMARY:

Note that depending on the phase of development, parts of the testing may involve the participant interacting directly with the computer tool (e.g., the preference assessment tool) or viewing a screen shot of what the computer screen is intended to display or print. Not all questions below will necessarily be asked of every participant, the questions asked will depend on how far along the study has progressed at the time of the interview.

Introduction: Explain study procedures (as described in the ICF that the participant would have already reviewed and consented); request permission to audio record the interview and to share the participant’s computer screen.

Interview: All interviews will be audio recorded (with permission) and the interviewer will take notes throughout. The test will begin with a less structured segment in which participants are asked to interact with the preference assessment tool without specific guidance. The participant will then be given specific tasks to perform (i.e., find a specific piece of information). Time to completion and the path navigated will be monitored. This will determine usability of the user-interface format. Users will be encouraged to "think aloud" as they navigate through the tool and may be asked to rate their impressions of each component (page) and of the tool overall.

Closing: Thank participant; mention that gift card will be emailed; ask for permission to recontact them again for feedback on later iterations of the templates or for participating in other phases of study.

INTERVIEW DETAILS (after obtaining informed consent online)

Introduction

Explanation of Study Procedures:

My name is [name] and I’ll be conducting the interview today. I appreciate that you took time out of your busy schedules to come here. The first thing I’d like to do is restate why we’ve asked you to be here to make sure our purpose is clear.

This interview is part of a study sponsored by Shared Decision Making Resources under a Scientific Research Agreement from Edwards Lifesciences to better understand what's important to patients with Aortic Stenosis and their health care providers when they make decisions about their health or choosing treatment. Findings will be used to develop tools and resources to help patients with AS choose treatment that is more consistent with their own preferences and values. We are at the [design phase] and would like to show you some of the parts that might appear on the [website]. We hope that you will go through the parts to see if it is easy or hard to use them or get information from them. Based on what you tell us, the design will be modified and improved.

Our interview will be informal, so please feel free to share your thoughts as they come to you. We are interested in a variety of opinions, so please let us know what’s on your mind. This isn’t a test of you but of the design, so there’s no such thing as a right or wrong answer. There may well be important design issues that you can help us identify and fix. Since our goal is to improve the design, you don’t have to worry about hurting my feelings with anything you may say. You can choose not to answer the questions discussed if you do not feel comfortable.

We’re going to be recording our discussion today, if you agree, but you will not be individually identified in any reports of this research. I’ll only use your first name to protect your privacy. Nothing you say today will be reported to your doctor or other health professional.

Part 1 - Unguided Usage of Preference Assessment tool and SDM feedback page:

The subject is asked to view the first screen or page of the website on their computer and to share their computer screen (using Zoom software), and told:

Imagine you have been asked by your health care provider to visit this website. Please go through the website as you normally would if you were alone.

- Please speak freely about your thoughts, comments, concerns and suggestions as you interact with the website.
- Oral questions
- What are your initial impressions of the site [page]?
- What do you think the site is for? How can you tell?
- Oral Questions when they are finished running through the site
  - Do you have any questions or comments?
  - What did you like about the web site?
  - What did you not like about the web site?
  - Would you like to see anything added?
  - What would you like to see removed?
  - How do you feel about the graphics?
  - How do you feel about the design of the web site [page] (including look, feel, colors, font)?
  - What would make the website more interesting, appealing, or useful?
  - Is the text size readable?
  - Additional comments?

Part 2 - Guided Discussion to obtain feedback on specific issues.

2a. Introduction /welcome page:

- How was the introduction?
- Did anything on this page seem confusing? If yes, why? (e.g. layout, wording)
- What is your overall impression of this page (look, feel, colors, font)?
- Are there any changes you would suggest? If yes, what?

2b. Preference Assessment Page (and other intake questions)

- Could you complete the question?
- Did any of the questions seem confusing? If yes, why? (e.g. layout, wording)
- What do you think about the way this page looks (look, colors, font)?
- Are there any other changes you would suggest? If yes, what?
- Is there anything you would add or remove? If yes, what?

*2c. Feedback page: SDM summary page*

*Script:* Here is a summary page that the tool will generate for patients. We would like you to read through it and tell us what you think of it. Again, we want to make this page helpful for patients, so don't worry about hurting my feelings with anything you say.

- Is there anything about the wording or layout that seems confusing?
- Does the page look useful?
- Are there any changes you would make; anything you would add or remove? If yes, what?
- Does this accurately summarize your preferences, values, and main concerns?
- Did any of the sections seem confusing? If yes, why? (e.g. layout, wording)
- What do you think about the way this page looks (look, colors, font)?
- What did you think of the graphics? Tables?
- What would you do with this information?

*Part 3 - Information Seeking Tasks*

*During each task, the proctor will be minimally involved. He/she will monitor how long it takes the participant to complete the task/scenario and whether it was completed. She will also monitor any questions asked and the computer system’s functionality. At the end of each task the proctor will ask the user the following questions:*

- How easy or hard was it to complete the task?
- Are you satisfied with the results?
- How could we make it easier to use?

*Script:* Now I would like you to try to find the answers to a few questions. All of the information you need is available somewhere on the website. You don't have to write anything down, just let me know when you have found the information. I will then verify that the task has been completed. We are not interested in the actual answers to the questions (you may already know them), but rather whether you can find the information on the web site. There are no trick questions. These tasks will test how well the website [page] is organized and how easy it is to navigate.

Questions for participant:

- Who made this website?
- What is this website for?
- Once you get this information, can you go back to where you were before?
- What happens to the information you enter on this website?
- Can you go back and change your answers to one of your questions

4. SDM summary page for HCPs

*Script:* I would like you to pretend that you have finished using the Preference Assessment tool and are now seeing your doctor, nurse, or physician's assistant. The tool has created a page that summarizes your personal preferences and treatment goals for AS. I am going to show you what this page might look like and I would like to know what you think of it and how you might want to change it. We are trying to make the page as helpful as possible, so don't worry about hurting my feelings with anything you say.

- *[show the SDM summary here]* What did you think of this page?
- What changes would you want to make to it?
- Does this accurately summarize your preferences, values, and main concerns?
- Did anything on this page seem confusing? If yes, why? (e.g. layout, wording)
- Were there any topics missing?
- What do you think about the way this page looks (look, colors, font)?
- What did you think of the graphics? Tables?
- Are there any other changes you would suggest? If yes, what?
- Would you want to share this information with your cardiologist? Your surgeon? With your primary care doctor? With other HCPs? With your caregiver?
- Why or why not?
- Now imagine that you don't want to talk about these topics with your doctor. What would you do now?
- Is there any specific information here that you would **not** want to share with your HCPs?
- Is there a topic you don't see listed that should be on the list?
- Do you think sharing this summary page with your cardiologist would help you choose the best treatment for your AS?

4a. Preference Checklist/summary

- Were there any topics missing?
- Did anything on this page seem confusing? If yes, why? (e.g. layout, wording)
- What do you think about the way this page looks (look, colors, font)?
- Are there any other changes you would suggest? If yes, what?

4b. Brief evaluation of the Preference Summary Page

- These are some questions asking what you thought of this website. Can you complete these?
- Did any of the questions seem confusing? If yes, why? (e.g. layout, wording)
- What do you think about the way this page looks (look, colors, font)?
- Are there any other changes you would suggest? If yes, what?

Part 5 - Evaluation Questionnaires

(Not all subjects will complete this section.)

*Script:* Here are some other questions that we will be asking people who use the tool to fill out. Can you take a look at them and tell us what you think of them?

- - Is there anything about the wording or layout that seems confusing?
  - Is there anything else that you think we should ask?

Part 6 – Debriefing (optional)

Feel free to let me know any overall thoughts and comments.

1. Do you have any questions about the [preference assessment Tool]?
2. What did you like the most about it?
3. What did you like the least?
4. Do you have any other suggestions for improving the Tool?
5. Would you recommend the Tool to your friends who have AS?

Thank you for helping us improve the Preference tool.

*Mention that the gift card will be emailed to them. Ask for permission to contact them again to ask for more feedback or to ask if they want to participate in another phase of the study.*

## Appendix G: Patient Outreach and Reminder Emails (Phases 1-4)

### Email #1: Response to emails inquiring about participation

**Subject line:** Invitation to the ASPIRE study

Greetings!

Thank you for your interest in the ASPIRE Study. This research study aims to help patients make decisions about treating aortic stenosis. (Aortic Stenosis is a narrowed heart valve that doesn’t open properly.)

Your experience is needed to help [newly diagnosed] patients express their treatment preferences and to help healthcare providers understand what's important to their patients.

- You may be eligible to participate if you have been treated for [diagnosed with] aortic stenosis.
- You can participate online, in-person, or over the phone.
- You will be asked to think about what [matters] [mattered] to you when choosing treatment for aortic stenosis.
- [You may be given access to a new online doctor-patient tool.]
- You will be **paid for your time and effort** for participating.

If you are eligible to participate, you can choose which activity interests you. Each study activity involves different levels of involvement on your part. Participation in more than one activity is possible.

Click here to see if you qualify.

Your participation in this study is completely voluntary and you can withdraw at any time by contacting the lead researchers (contact information below).

[There are a limited number of slots available - so be sure not to delay!] 

Thank you,

Nananda Col, MD, MPH, MPP, FACP and Megan Coylewright, MD

Lead Researchers

The **ASPIRE Study** (**A**ortic **S**tenosis **P**references **I**n T**RE**atment)

[ASPIRE@SDMR.US](mailto:ASPIRE@SDMR.US).

### Email #2: General Outreach

**Subject line: [Your voice matters] [Do you have AORTIC STENOSIS]**

**Have you have been treated for AORTIC STENOSIS (**a narrowed heart valve)**?**

W**e need your help!**

We are trying to help patients make decisions about treating aortic stenosis and help healthcare providers learn how patients make their decisions.

- Your experience is needed to help [newly diagnosed] patients express their treatment preferences and to help healthcare providers understand what's important to their patients.
- You can participate online, in-person, or over the phone.
- You may be eligible to participate in this research study if you have been treated for [diagnosed with] aortic stenosis.
- You will be asked to think about what [matters] [mattered] to you when choosing treatment for aortic stenosis.
- [You may be given access to a new online doctor-patient tool.]
- You will be paid for your time and effort.

**Click here** [to see if you qualify.]

Your participation in this study is completely voluntary and you can withdraw at any time by contacting the lead researchers (contact information below).

Thank you,

Nananda Col, MD and Megan Coylewright, MD

Lead Researchers

The **ASPIRE Study** (**A**ortic **S**tenosis **P**references **I**n T**RE**atment)

Email: [ASPIRE@SDMR.US](mailto:ASPIRE@SDMR.US)

Phone: 207-272-9829

### Email #3: Targeted Outreach for Specific Activities

Greetings!

Shared Decision Making Resources is conducting a research study to test a new way to help people with **aortic stenosis** make decisions about treating aortic stenosis. (Aortic Stenosis is a narrowed heart valve that doesn’t open properly.)

At this time we seek adults with aortic stenosis to take part in [small group meetings] in [town, state]], [a conference call] [an online survey].

- You will be asked to think about what [matters] [mattered] to you when choosing treatment for aortic stenosis.
- You will be paid for your time and effort.

Click here to see if you qualify.

Your participation in this study is completely voluntary and you can withdraw at any time by contacting the lead researchers (contact information below).

[There are a limited number of slots available - so be sure not to delay!] 

Thank you,

Nananda Col, MD and Megan Coylewright, MD

Lead Researchers

The **ASPIRE Study** (**A**ortic **S**tenosis **P**references **I**n T**RE**atment)

Email: [ASPIRE@SDMR.US](mailto:ASPIRE@SDMR.US)

Phone: 207-272-9829

### Email #4: Follow-up Email about in-person NGT Group Meeting

Dear Mr/Ms. [NAME],

Thank you for agreeing to participate in the group discussion/online forum that Shared Decision Making Resources is conducting [on DATE at TIME at the LOCATION]. [A map and directions are attached to show you how to get to the LOCATION]. We will be meeting in the [NAME] room. [There will be someone available at the door [or signs] to show you where that room is located.]

The purpose of this group discussion is to understand more about what's important to patients when they make decisions about treating aortic stenosis. You will be part of a group of five to ten people with aortic stenosis from the local area. We are very interested in learning more about your thoughts regarding how you managed your aortic stenosis.

The session will begin at TIME and will end by TIME. We know how valuable your time is, and we will respect everyone’s schedules by starting and ending on time. Please allow yourself enough time to reach the LOCATION by TIME.

We will provide [a light snack][lunch][dinner] and email you an Amazon gift card worth $50.00 after the meeting. We will audio record parts of the discussion so that we can keep a careful record of things that we hear from you and the other participants. We will take every step possible to maintain your privacy to the extent of the law.

We are so glad you have accepted our invitation to participate in this study. If you cannot attend for any reason, please email us at ASPIRE@SDMR.US or call us at (207) 272-9829 or [local number] as soon as possible.

Sincerely,

Nananda Col, MD and Megan Coylewright, MD

Lead Researchers

The **ASPIRE Study** (**A**ortic **S**tenosis **P**references **I**n T**RE**atment)

Email: [ASPIRE@SDMR.US](mailto:ASPIRE@SDMR.US)

Phone: 207-272-9829

### Email #5: Follow-up email for Online NGT Forums

Dear Mr/Ms xxx,

Thank you for agreeing to participate in the online Forum that Shared Decision Making Resources is conducting, starting [on DATE]. You will receive an email which will contain a link that you can click on to get started. You do not need any specialized equipment or software to participate. The Online Forum will take place over the course of 2-14 days so you will not have to respond to anything immediately. You will receive a series of online messages that will each give you specific instructions and questions to answer. The entire activity should take no longer than 60 minutes. Please ensure that you will have sufficient time during this time to thoughtfully answer the questions posed to you. You can answer questions at your own pace and time.

The **purpose** of this online Forum is to understand more about what's important to patients when they make decisions about their aortic stenosis. You will be part of an online panel of six to ten (anonymous) people with aortic stenosis from around the country. We are interested in learning more about your thoughts and decisions regarding your health.

We will email you an Amazon gift certificate worth $50.00 after you complete the Online Forum. We will take every step possible to maintain your privacy to the extent of the law.

We are so glad you have accepted our invitation to participate in this study. If you cannot participate for any reason, please email us at ASPIRE@SDMR.US as soon as possible.

Sincerely,

Nananda Col, MD and Megan Coylewright, MD

Lead Researchers

The **ASPIRE Study** (**A**ortic **S**tenosis **P**references **I**n T**RE**atment)

Email: [ASPIRE@SDMR.US](mailto:ASPIRE@SDMR.US)

Phone: 207-272-9829

### Email #6: - Pilot Study: Response to emails from patients inquiring about participation

Subject: Invitation to The AVITA study

[Greetings!]

Thank you for your interest in the AVITA Study. This research study aims to help patients with decisions about treating aortic stenosis. (Aortic Stenosis is a narrowed heart valve that doesn’t open properly.)

- You may be eligible to participate if you have been diagnosed with aortic stenosis and are scheduled to see a cardiology healthcare provider to discuss your treatment options.
- You will be given access to a new online learning tool that focuses on treatment for aortic stenosis.
- All study surveys are online.
- You will be **paid for your time and effort** for participating.

Click here to see if you qualify.

Click here to learn more about the study.

Your participation in this study is completely voluntary and you can withdraw at any time by contacting the lead researchers (contact information below).

[There are a limited number of slots available - so be sure not to delay!]

Thank you,

Nananda Col, MD, MPH, MPP, FACP and Megan Coylewright, MD

Lead Researchers

The AVITA Study

**A**ortic **V**alve **I**mproved **T**reatment **A**pproaches

info@valvesurvey.com

Follow the link to opt out of future emails:
${l://OptOutLink?d=Click here to unsubscribe}

### Email #7: Pilot Study: Patient –Invitation for T2 survey (after HCP appointment)

Subject: Time to take your final AVITA survey

Dear ${m://FirstName},

If you just had an appointment with your valve specialist, it’s time to take the last AVITA survey.

Click **here** to take the survey

- This survey will ask about your recent appointment with your cardiology provider and your treatment preferences.
- When you complete the survey, you will be emailed an Amazon gift card.

If your doctor’s appointment was **rescheduled or cancelled**, please let us know by clicking on the survey link above. The survey will let you change your appointment date so that we can send you the survey at the right time.

We really appreciate your involvement in the study and hope that you will be able to complete this survey.

Thank you,

Nananda Col, MD, MPH, MPP, FACP and Megan Coylewright, MD

Lead Researchers

The AVITA Study

**A**ortic **V**alve **I**mproved **T**reatment **A**pproaches

info@valvesurvey.com

Follow the link to opt out of future emails:
${l://OptOutLink?d=Click here to unsubscribe}

### Email #8: Pilot Study: Patient – Survey reminder for T0 or T2 survey

Subject: Reminder to take the final MS-SUPPORT Survey

Message:

Dear [Name],

Just a friendly reminder to please [take] [complete] the [next][last] survey for the AVITA study.

**Follow this link to the Survey:**
${l://SurveyLink?d=Take the Survey}

Or copy and paste the URL below into your internet browser:
${l://SurveyURL}

We greatly appreciate your involvement in the study and hope that you will be able to complete this final survey. You will receive a $[30][45] Amazon gift card after you complete the survey. [If you need any help or have questions, please feel free to contact us ([info@valvesurvey.com)](mailto:info@valvesurvey.com)).]

[We look forward to your continued involvement and are so grateful for your support!]

[Thank you for being part of this study!]

Nananda Col, MD, MPH, MPP, FACP and Megan Coylewright, MD

Lead Researchers

The AVITA Study

**A**ortic **V**alve **I**mproved **T**reatment **A**pproaches

info@valvesurvey.com

Follow the link to opt out of future emails:
${l://OptOutLink?d=Click here to unsubscribe}

### Email #9: Pilot Study: Patient – Survey reminder (alternative)

Subject: The AVITA Study – Please complete the AVITA survey [Survey Name] by [Date]

[Dear][Hello] [patient name],

My name is [team member name] and I am part of the AVITA Study team. I am reaching out [again] because we noticed that you haven’t completed [Survey Name].

We hope you can complete the survey below at your earliest convenience.

**[Survey Link]**

[If you need any help or have questions, please feel free to contact us ([info@valvesurvey.com](mailto:info@valvesurvey.com), [207-272-9829]).]

We look forward to your continued involvement and are so grateful for your support!

Thank you,

[team member name]

[title]

The AVITA Study

Shared Decision Making Resources

If you no longer want to participate in this study, please email us at ([info@valvesurvey.com](mailto:info@valvesurvey.com)) or click the opt-out link below.

Follow the link to opt out of future emails:
${l://OptOutLink?d=Click here to unsubscribe}

### Email #10: Pilot Study: HCP - Survey Reminder 1

Hello [HCP name],

My name is [team member name] and I am part of the AVITA Study team. I am reaching out [again] because we noticed that you haven’t completed [Survey Name].

Please complete the survey I have linked below at your earliest convenience.

If you need any help or have questions, please feel free to contact us (info@valvesurvey.com).

We look forward to your continued involvement and are so grateful for your support!

[Survey Link]

Thank you,

[team member name]

[title]

The AVITA Study

Shared Decision Making Resources

If you no longer want to participate in this study, please email us at ([info@valvesurvey.com](mailto:info@valvesurvey.com)) or click the opt-out link below.

[Qualtrics Opt-Out Link]

### Email #11: Pilot Study: HCP - Survey Reminder 2

Subject: The AVITA Study – Checking In

Hello [HCP name],

My name is [team member name] and I am part of the AVITA Study team. I am reaching out because we noticed that we haven’t heard back from you in a while.

If you would still like to be a part of the AVITA Study please complete the survey I have linked below.

If you need any help or have questions, please feel free to contact us (info@valvesurvey.com).

We look forward to your continued involvement and are so grateful for your support!

[Survey Link]

Thank you,

[team member name]

[title]

The AVITA Study

Shared Decision Making Resources

If you no longer want to participate in this study, please contact us at [info@valvesurvey.com](mailto:info@valvesurvey.com).

[Qualtrics Opt-Out Link]

### Email #12: Pilot Study: HCP - Patient/HCP Missing / incomplete data follow-up

Subject: The AVITA Study – Following up about your recent survey

Hello [HCP name],

My name is [team member name] and I am part of the AVITA Study team. I am reaching out because we had a question about your survey response(s). At your earliest convenience, can you answer the question below?

[*Description of event of missing data*]

If you need any help or have questions, please feel free to contact us (info@valvesurvey.com).

We look forward to your continued involvement and are so grateful for your support!

[Survey Link]

Thank you,

[team member name]

[title]

The AVITA Study

Shared Decision Making Resources

If you no longer want to participate in this study, please email us at (info@valvesurvey.com).

Follow the link to opt out of future emails:
${l://OptOutLink?d=Click here to unsubscribe}

## Appendix H. Study Outreadh—Ads, flyers, talking points

### Appendix H1: Study Flyer

**Have you been treated for Aortic Stenosis?**

(Aortic Stenosis: a narrowed heart valve that fails to open properly)

**We need your help for our research study!**

You will be paid for your time and effort.

**What’s involved?**

| - Help us understand what you think is important when making decisions about treatment. - You can participate in online surveys or small group meetings, at your convenience. |
| --- |
|  |
| **FOR MORE INFORMATION:**  **email: ASPIRE@SDMR.US** |

***This research study is being conducted by Dr. Nananda Col, Shared Decision Making Resources, Georgetown, ME and Dr. Megan Coylewright, Dartmouth-Hitchcock Medical Center, Lebanon, New Hampshire.***

### Appendix H2: Wallet-size Card 1


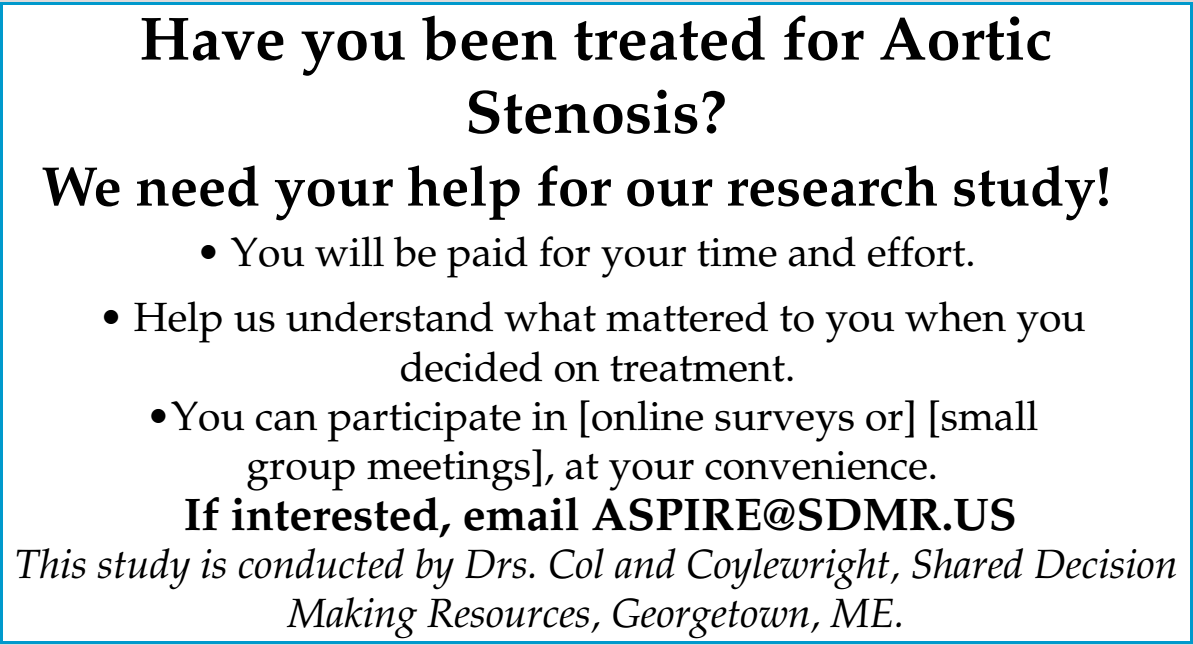


### Appendix H3: Wallet-size Card 2 (for untreated patients)


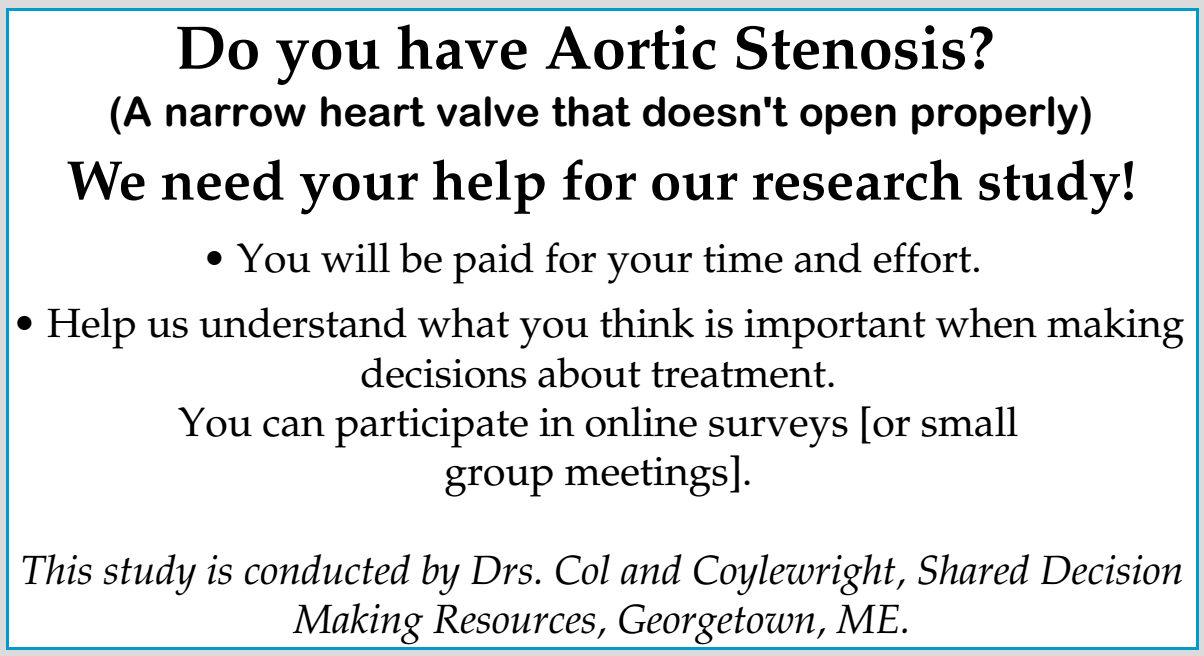


### Appendix H4: Pilot Study Wallet-size Card (for patients facing a decision about treatment)

Below are the proofs to be used for printing:

*Front side*:


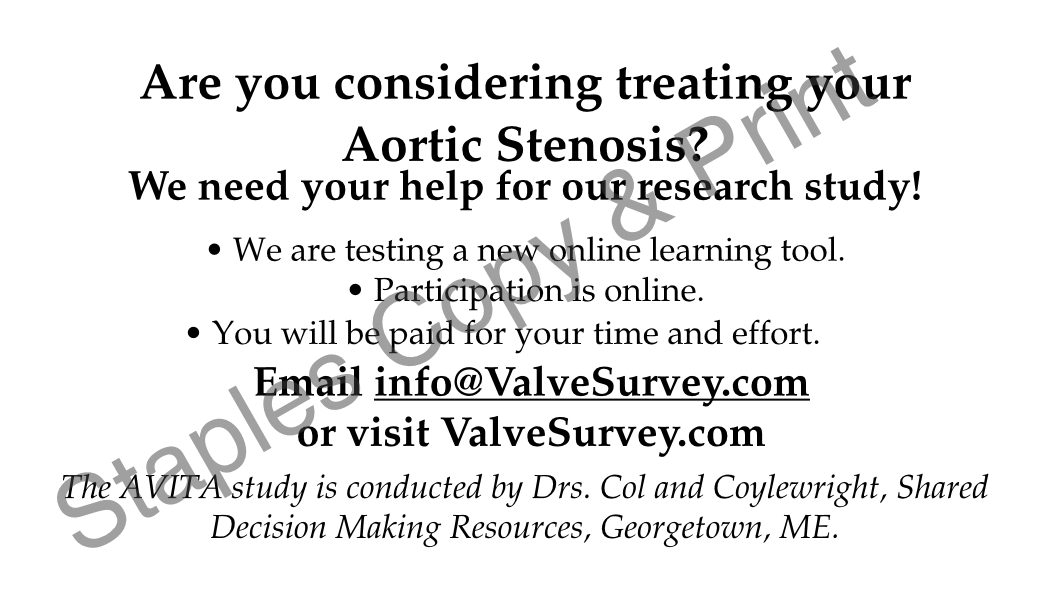


*Back side:*

### Appendix H5: Pilot Study Flyer (for patients facing a decision about treatment)

**Do you have Aortic Stenosis?**

- **Do you need a new valve?**
- **Are you considering treatment?**
- **Do you have an appointment to discuss treatment?**

**If so, we need your help for our research study!**

- We are testing a new online learning tool designed to help people explore their treatment options.
- You will be paid for your time and effort.

**What’s involved?**

| - Complete 2 online surveys and view the AVITA learning tool over the next few weeks. - AVITA asks about your treatment goals and preferences. - AVITA gives you personalized feedback as well as a printable summary of your goals and preferences that you can share with your heart team. |
| --- |
| **FOR MORE INFORMATION: Valvesurvey.com**  or  **Email:** [**Info@Valvesurvey.com**](mailto:Info@Valvesurvey.com) |

***This research study is being conducted by Dr. Nananda Col, Shared Decision Making Resources, Georgetown, ME and Dr. Megan Coylewright.***

### Appendix H6: Pilot Study Talking Points (for potential patient participants)

Talking Points Phone calls

*These talking points can be used when contacting a patient with severe aortic stenosis with an appointment within 6 weeks to discuss treatment options:*

1. [Dr. xxx/Ms xxx] is taking part in a study that helps people with aortic stenosis make better decisions with their healthcare provider. Everything is online and it is optional to participate.

1. If you complete the study, you will receive gift cards totaling $75.
2. If you would like to learn more about the study, we can share your email with Dr. Col, who is leading the study. Dr. Col can then email you more information.
3. Would you like us to send your email to Dr. Col?

If yes 🡪obtain email. [we do not need name or any other information]

If no 🡪 you may contact her at [**Info@Valvesurvey.com**](mailto:Info@Valvesurvey.com). Just write “I’m interested”.

Or you can learn more about the study at [valvesurvey.com](file:///C:\Users\NanandaCol\Documents\.AorticStenosis_Edwards\HCP%20outreach\valvesurvey.com\pageupdate).

**Questions**?

Please refer any questions about the study to Dr. Nananda Col: Phone: (207) 272-9829 or Email: [**Info@Valvesurvey.com**](mailto:Info@Valvesurvey.com)

## Appendix I: Intake Survey (Screening/baseline/consent) (Phases 1-4).

Aortic Stenosis Intake Survey – Copied directly from Qualtrics Server

Survey Flow:

Standard: Introduction (4 Questions)

Branch: New Branch If If Welcome to the ASPIRE Study&nbsp; &nbsp; (Aortic Stenosis Preference Integration REsource) &nbsp; This study hopes to better understand what's important to people with aortic stenosis when the... No Is Selected

Block: Message: not eligible (2 Questions)

EndSurvey:

Branch: New Branch If Welcome to the ASPIRE Study&nbsp; &nbsp; (Aortic Stenosis Preference Integration REsource) &nbsp; This study hopes to better understand what's important to people with aortic stenosis when the... Yes Is Selected

Block: Intake Screener: AS patients (16 Questions)

EmbeddedData

QState_Q22Value will be set from Panel or URL.

Block: Health literacy screener (Lisa Chew 2004) (1 Question)

Branch: New Branch If Aortic stenosis can cause “heart failure.” When that happens, you can get fluid in your lungs, le... Yes Is Selected

Standard: KCCQ-12:Heart Failure (8 Questions)

Branch: New Branch If Aortic stenosis can cause “heart failure.” When that happens, you can get fluid in your lungs, le... No Is Selected

Standard: KCCQ-12:AS (8 Questions)

Standard: single item health measure (1 Question)

Block: Other AS comorbidities (1 Question)

Block: Participation options (2 Questions)

Standard: ICFs (3 Questions)

Block: Scheduling (5 Questions)

EndSurvey:

| Page Break |  |
| --- | --- |

Start of Block: Introduction

Q1
**Welcome to the ASPIRE Study (A**ortic **S**tenosis **P**references **I**n t**RE**eatment**)**
  
This study hopes to better understand what's important to people with aortic stenosis when they make decisions about choosing treatment. It also aims to help newly diagnosed patients make decisions about treatment and help health care providers learn how patients make their decisions. Aortic Stenosis is a narrowed heart valve that doesn't open properly, resulting in impaired heart function.* Study findings will be used to help patients express their treatment preferences and help healthcare providers understand what's important to their patients. You can participate online, in-person, or by telephone. All study activities will address what matters when choosing treatment for aortic stenosis.  You can choose which activity you are interested in, if any, on another page of this survey. You will be paid for your time and effort. Please answer a few questions to see if you qualify to participate and to help us understand our participants. Have you ever been **told by a health care provider** that you have Aortic Stenosis?

- Yes (2)
- No (3)

| Page Break |  |
| --- | --- |

Display This Question:

If Welcome to the ASPIRE Study (Aortic Stenosis Preferences In tREeatment)   This study hopes to bet... = Yes

| 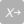 |
| --- |

Q2 Have you had a **valve replacement** for your aortic stenosis?

- Yes (1)
- No (0)

Display This Question:

If Have you had a valve replacement for your aortic stenosis? = No

Q3 **Are you pursuing medical management** instead of valve replacement?

- Yes (1)
- No (2)

Display This Question:

If Have you had a valve replacement for your aortic stenosis? = No

And Are you pursuing medical management instead of valve replacement? = No

Q4 Are you **planning on getting your valve replaced**?

- Yes (1)
- No (2)

End of Block: Introduction

Start of Block: Message: not eligible

Display This Question:

If Welcome to the ASPIRE Study (Aortic Stenosis Preferences In tREeatment)   This study hopes to bet... = No

Q5 You are not eligible to participate in this study because you do not have aortic stenosis.  Thank you for your interest.

Display This Question:

If Have you had a valve replacement for your aortic stenosis? = No

Q6 You are not eligible to participate at this time. Would you like to be contacted (be email) at a later time to see if you are eligible?

- Yes (1)
- No (2)

End of Block: Message: not eligible

Start of Block: Intake Screener: AS patients

| 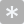 |
| --- |

Q7 What **year** did you learn that you had aortic stenosis? If you are not sure, please give your best guess.

________________________________________________________________

Display This Question:

If Have you had a valve replacement for your aortic stenosis? = Yes

Q8 How was your aortic stenosis treated? Check all that apply.

- **TAVR** (**Transcatheter** aortic valve replacement)
- **SAVR** (**Surgical** aortic valve replacement)

Display This Question:

If Have you had a valve replacement for your aortic stenosis? = Yes

| 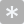 |
| --- |

Q9 What year did you have your aortic valve replacement?

________________________________________________________________

Display This Question:

If How was your aortic stenosis treated? Check all that apply. = <strong>SAVR </strong>(<strong>Surgical </strong>aortic valve replacement)

Q10 What type of valve did you receive?

- Mechanical valve (1)
- Tissue valve (also called "bioprosthetic," made from animal tissue) (2)
- I'm not sure (3)

Display This Question:

If How was your aortic stenosis treated? Check all that apply. = <strong>TAVR </strong>(<strong>Transcatheter </strong>aortic valve replacement), or transcatheter aortic valve implantation (TAVI)

Or How was your aortic stenosis treated? Check all that apply. = <strong>SAVR </strong>(<strong>Surgical </strong>aortic valve replacement)

| 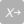 |
| --- |

Q11 Since your original valve replacement for aortic stenosis, have any of the following happened? Please select all that apply.

- Another aortic valve replacement (1)
- Balloon valvuloplasty (also called "valvulotomy" or "valvotomy") (5)
- Another cardiac surgery, not related to the aortic valve (0)
- A stent procedure (often called PCI) (2)
- A pacemaker (6)
- One or more additional hospitalization (not counting the one for your valve replacement) (3)
- None of the above (4)

| Page Break |  |
| --- | --- |

Q13
Aortic stenosis can cause “heart failure.” When that happens, you can get fluid in your lungs, legs, and belly which can cause shortness of breath and fatigue.

 
Do you **now** have heart failure?

- Yes (2)
- No (3)

| 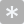 |
| --- |

Q14 How old are you?

________________________________________________________________

Display This Question:

If If How old are you? Text Response Is Less Than 18

Q15 I'm sorry but you are not eligible to participate in this study.  Thank you for your interest.

Skip To: End of Survey If I'm sorry but you are not eligible to participate in this study. Thank you for your interest.() Is Displayed

Q16
What is your name? 


We keep all of your information and personal identifiers encrypted and strictly confidential.

- First name (4) ________________________________________________
- Last name (5) ________________________________________________

| 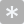 |
| --- |

Q17
Please enter the best email address to contact you.


 If you are eligible for the study, we will use this email to contact you.

________________________________________________________________

| 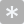 |
| --- |

Q18 Please re-enter your email address.

________________________________________________________________

Q19 **What is your gender**?

- Male (1)
- Female (2)
- Choose not to respond (3)

Q20 Which racial group or groups best describes you?

- Hispanic or Latino (1)
- Black or African American (2)
- White (3)
- Asian (4)
- Native American or Alaska Native (5)
- Native Hawaiian or other Pacific Islander (6)
- Other (please specify) (7) ________________________________________________
- Choose not to respond (8)

Q21 What is the highest grade in school you completed?

- Less than high school (1)
- High school graduate or GED (2)
- Some college (3)
- Two year college or technical school (4)
- College graduate (5)
- Graduate school or professional degree (6)

| 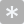 |
| --- |

Q22 What is your zip code (postal code) for your primary residence?

________________________________________________________________

| 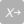 |
| --- |

Q23 What type of health insurance do you presently have?

- Medicaid or other type of state medical assistance (5)
- Medicare (4)
- Private health insurance offered through an employer or a union (1)
- Private health insurance paid by the individual, such as The Patient Protection and Affordable Care Act (ACA, or "Obamacare") (2)
- Military or Veterans (VA) (3)
- Uninsured (0)
- Other (9) ________________________________________________

End of Block: Intake Screener: AS patients

Start of Block: Health literacy screener (Lisa Chew 2004)

| 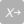 |
| --- |

Q24 How often do you need to have someone help you when you read instructions, pamphlets, or other written material from your doctor or pharmacy?

- Never (1)
- Rarely (2)
- Sometimes (3)
- Often (4)
- Always (5)

End of Block: Health literacy screener (Lisa Chew 2004)

Start of Block: KCCQ-12:Heart Failure

Q25

 The following questions refer to your heart failure and how it may affect your life. Please read and complete the following questions. There are no right or wrong answers. Please mark the answer that best applies to you. 
Heart failure affects different people in different ways. Some feel shortness of breath while others feel fatigue.
 
**Please indicate how much you are limited by heart failure (shortness of breath or fatigue) in your ability to do the following activities over the past 2 weeks.**

|  | **Extremely** limited (1) | **Quite a bit** Limited (2) | **Moderately** Limited (3) | **Slightly** Limited (4) | **Not at all** Limited (5) | Limited for other reasons or did not do the activity (6) |
| --- | --- | --- | --- | --- | --- | --- |
| a. Showering/bathing (1) |  |  |  |  |  |  |
| b. Walking one block on level ground (2) |  |  |  |  |  |  |
| c. Hurrying or jogging (as if to catch a bus) (3) |  |  |  |  |  |  |

| Page Break |  |
| --- | --- |

Q26 Over the past 2 weeks, how many times did you have **swelling in your feet, ankles or legs when you woke up** in the morning?

- Every morning (1)
- 3 or more times per week but not every day (2)
- 1-2 times per week (3)
- Less than once a week (4)
- Never over the past 2 weeks (5)

Q27 Over the past 2 weeks, on average, how many times has **fatigue** limited your ability to do what you wanted?

- All of the time (1)
- Several times per day (2)
- At least once a day (3)
- 3 or more times per week but not every day (4)
- 1-2 times per week (5)
- Less than once a week (6)
- Never over the past 2 weeks (7)

| Page Break |  |
| --- | --- |

Q28 Over the past 2 weeks, on average, how many times has **shortness of breath** limited your ability to do what you wanted?

- All of the time (1)
- Several times per day (2)
- At least once a day (3)
- 3 or more times per week but not every day (4)
- 1-2 times per week (5)
- Less than once a week (6)
- Never over the past 2 weeks (7)

Q29 Over the past 2 weeks, on average, how many times have you been **forced to sleep sitting up in a chair or with at least 3 pillows to prop you up because of shortness of breath**?

- Every night (1)
- 3 or more times per week but not every day (2)
- 1-2 times per week (3)
- Less than once a week (4)
- Never over the past 2 weeks (5)

| Page Break |  |
| --- | --- |

Q30 Over the past 2 weeks, how much has your heart failure **limited your enjoyment of life**?

- It has extremely limited my enjoyment of life (1)
- It has limited my enjoyment of life quite a bit (2)
- It has moderately limited my enjoyment of life (3)
- It has slightly limited my enjoyment of life (4)
- It has not limited my enjoyment of life at all (5)

Q31 If you had to spend the rest of your life with your heart failure the way it is right now, how would you feel about this?

- Not at all satisfied (1)
- Mostly dissatisfied (2)
- Somewhat satisfied (3)
- Mostly satisfied (4)
- Completely satisfied (5)

| Page Break |  |
| --- | --- |

Q32 How much does your heart failure affect your lifestyle? Please indicate how your heart failure may have limited your participation in the following activities over the past 2 weeks

- A great deal (1)
- A lot (2)
- A moderate amount (3)
- A little (4)
- None at all (5)

End of Block: KCCQ-12:Heart Failure

Start of Block: KCCQ-12:AS

Q126

 The following questions refer to your aortic stenosis and how it may affect your life. Please read and complete the following questions. There are no right or wrong answers. Please mark the answer that best applies to you. 
Aortic stenosis can cause heart failure which affects different people in different ways. Some feel shortness of breath while others feel fatigue.
 
**Please indicate how much you are limited by aortic stenosis (shortness of breath or fatigue) in your ability to do the following activities over the past 2 weeks.**

|  | **Extremely** limited (1) | **Quite a bit** Limited (2) | **Moderately** Limited (3) | **Slightly** Limited (4) | **Not at all** Limited (5) | Limited for other reasons or did not do the activity (6) |
| --- | --- | --- | --- | --- | --- | --- |
| a. Showering/bathing (1) |  |  |  |  |  |  |
| b. Walking one block on level ground (2) |  |  |  |  |  |  |
| c. Hurrying or jogging (as if to catch a bus) (3) |  |  |  |  |  |  |

| Page Break |  |
| --- | --- |

Q127 Over the past 2 weeks, how many times did you have **swelling in your feet, ankles or legs when you woke up** in the morning?

- Every morning (1)
- 3 or more times per week but not every day (2)
- 1-2 times per week (3)
- Less than once a week (4)
- Never over the past 2 weeks (5)

Q128 Over the past 2 weeks, on average, how many times has **fatigue** limited your ability to do what you wanted?

- All of the time (1)
- Several times per day (2)
- At least once a day (3)
- 3 or more times per week but not every day (4)
- 1-2 times per week (5)
- Less than once a week (6)
- Never over the past 2 weeks (7)

| Page Break |  |
| --- | --- |

Q129 Over the past 2 weeks, on average, how many times has **shortness of breath** limited your ability to do what you wanted?

- All of the time (1)
- Several times per day (2)
- At least once a day (3)
- 3 or more times per week but not every day (4)
- 1-2 times per week (5)
- Less than once a week (6)
- Never over the past 2 weeks (7)

Q130 Over the past 2 weeks, on average, how many times have you been **forced to sleep sitting up in a chair or with at least 3 pillows to prop you up because of shortness of breath**?

- Every night (1)
- 3 or more times per week but not every day (2)
- 1-2 times per week (3)
- Less than once a week (4)
- Never over the past 2 weeks (5)

| Page Break |  |
| --- | --- |

Q131 Over the past 2 weeks, how much has your aortic stenosis **limited your enjoyment of life**?

- It has extremely limited my enjoyment of life (1)
- It has limited my enjoyment of life quite a bit (2)
- It has moderately limited my enjoyment of life (3)
- It has slightly limited my enjoyment of life (4)
- It has not limited my enjoyment of life at all (5)

Q132 If you had to spend the rest of your life with your aortic stenosis the way it is right now, how would you feel about this?

- Not at all satisfied (1)
- Mostly dissatisfied (2)
- Somewhat satisfied (3)
- Mostly satisfied (4)
- Completely satisfied (5)

| Page Break |  |
| --- | --- |

Q133 How much does your aortic stenosis affect your lifestyle? Please indicate how your aortic stenosis may have limited your participation in the following activities over the past 2 weeks

- A great deal (1)
- A lot (2)
- A moderate amount (3)
- A little (4)
- None at all (5)

End of Block: KCCQ-12:AS

Start of Block: single item health measure

Q104 In general, would you say your health is...?

- Excellent (1)
- Very good (2)
- Good (3)
- Fair (4)
- Poor (5)

End of Block: single item health measure

Start of Block: Other AS comorbidities

Q34 Which of the following apply to you? Please check all that apply.

- Diabetes (1)
- COPD (chronic obstructive pulmonary disease) or emphysema (3)
- Previous stroke (cerebral vascular disease) (4)
- Previous heart attack (myocardial infarction) (5)
- Peripheral vascular disease (also known as PVD or Peripheral Artery Disease) (8)
- Atrial Fibrillation (A-Fib or an irregular heart beat) (6)
- Kidney disease (2)

End of Block: Other AS comorbidities

Start of Block: Participation options

Q35 **Thank you!** 

There are different ways that you can be involved in this study. All involve thinking about treatment decisions for aortic stenosis.  Please select the options that interest you. Some are available now, some will start later. away. For those that start later, we can contact you when they begin.

QState_Q22 = NH

Or QState_Q22 = TN

Or QState_Q22 = DC

Or QState_Q22 = VA

Or QState_Q22 = MD

- **Small group in-person meetings** of up to ten people from your local area that will last up to 90 minutes. You will be paid for your time and effort. (1)
- **Online small group forums** of up to ten people. You can answer questions online at your convenience, the total amount of time is about 60-90 minutes. You will be paid for your time and effort. (2)
- **An on-line survey**  that will take about [20-30] minutes to complete. You will be paid for your time and effort. (3)
- **A conference call** with a member of the study team who will share a computer screen online with you. The call will last [20 to 30] minutes]. You will be paid for your time and effort. (4)

| Page Break |  |
| --- | --- |

Display This Question:

If Thank you!  There are different ways that you can be involved in this study. All involve thinking... = <strong>An on-line survey </strong> that will take about [20-30] minutes to complete. You will be paid for your time and effort.

Or Thank you!  There are different ways that you can be involved in this study. All involve thinking... = <strong>Online small group forums</strong> of up to ten people. You can answer questions online at your convenience, the total amount of time is about 60-90 minutes. You will be paid for your time and effort.

Q36 Thank you! You selected one or more activities that have not yet been scheduled. We will contact you soon (by email) about the activity or activities that you selected.

End of Block: Participation options

Start of Block: ICFs

Display This Question:

If Thank you!  There are different ways that you can be involved in this study. All involve thinking... = <strong>A </strong><strong>conference call</strong> with a member of the study team who will share a computer screen online with you. The call will last [20 to 30] minutes]. You will be paid for your time and effort.

Q37 **Consent Form for Participation in a Research Study** 

 **Study Title**: The **ASPIRE Study** (**A**ortic **S**tenosis: **P**references **I**n T**RE**atment)
  
[INSERT RELEVANT CONSENT FORM HERE]

 **Statement of consent**

 I have read the above consent form and agree to participate in this study and I am over 18 years of age.

- Yes, I agree to participate (1)
- No, I do not agree (2)

Display This Question:

If Thank you!  There are different ways that you can be involved in this study. All involve thinking... = <strong>Small group in-person meetings</strong> of up to ten people from your local area that will last up to 90 minutes. You will be paid for your time and effort.

Q38 INSERT ICF for in-person NGT here

- Yes, I agree to participate (1)
- No, I do not agree (2)

Display This Question:

If Thank you!  There are different ways that you can be involved in this study. All involve thinking... = <strong>Online small group forums</strong> of up to ten people. You can answer questions online at your convenience, the total amount of time is about 60-90 minutes. You will be paid for your time and effort.

Q39 **INSERT Consent Form for Participation in a Research Study** 
 [ONLINE NGT]

 I have read the above consent form and agree to participate in this study and I am over 18 years of age.

- Yes, I agree to participate (1)
- No, I do not agree (2)

End of Block: ICFs

Start of Block: Scheduling

Display This Question:

If Thank you!  There are different ways that you can be involved in this study. All involve thinking... = <strong>Small group in-person meetings</strong> of up to ten people from your local area that will last up to 90 minutes. You will be paid for your time and effort.

Q40 To help us schedule a meeting, please check any of the following:

- I live in or near Hanover, NH (1)
- I live in or near [ ] (2)
- I live in or near [] (3)
- I live in or near [] (4)

Display This Question:

If Thank you!  There are different ways that you can be involved in this study. All involve thinking... = <strong>Small group in-person meetings</strong> of up to ten people from your local area that will last up to 90 minutes. You will be paid for your time and effort.

Q41 Please let us know if you have any specific scheduling requests or travel limitations. We will do our best to accommodate your needs. If you prefer that we contact you by telephone to schedule the meeting or discuss arrangements, please list your telephone number below.

________________________________________________________________

________________________________________________________________

________________________________________________________________

________________________________________________________________

________________________________________________________________

Display This Question:

If Thank you!  There are different ways that you can be involved in this study. All involve thinking... = <strong>Small group in-person meetings</strong> of up to ten people from your local area that will last up to 90 minutes. You will be paid for your time and effort.

Q42 Thank you!  We'll  be in touch soon by email to let you know next steps.

Display This Question:

If Thank you!  There are different ways that you can be involved in this study. All involve thinking... = <strong>A </strong><strong>conference call</strong> with a member of the study team who will share a computer screen online with you. The call will last [20 to 30] minutes]. You will be paid for your time and effort.

Q43 To simplify scheduling a call, please let us know what time of day generally works best for you in the next week or two. You will need access to either a computer connected to the internet, an i-phone, or an i-pad. The call will last [20-30] minutes  Choose as many times as you like.

|  | Monday (1) | Tuesday (2) | Wednesday (3) | Thursday (4) | Friday (5) |
| --- | --- | --- | --- | --- | --- |
| 10-12 AM (Eastern Time) (1) |  |  |  |  |  |
| 12-2 PM (2) |  |  |  |  |  |
| 2-4 PM (3) |  |  |  |  |  |
| 4-6 PM (4) |  |  |  |  |  |
| 6-8 PM (5) |  |  |  |  |  |

Display This Question:

If Thank you!  There are different ways that you can be involved in this study. All involve thinking... = <strong>A </strong><strong>conference call</strong> with a member of the study team who will share a computer screen online with you. The call will last [20 to 30] minutes]. You will be paid for your time and effort.

Q44 Please let us know if you have any specific scheduling requests. We will do our best to accommodate your schedule.

________________________________________________________________

________________________________________________________________

________________________________________________________________

________________________________________________________________

________________________________________________________________

End of Block: Scheduling

## Appendix J. Informed Consent Forms (ICF)

### Appendix J1: Cognitive Interview ICF

**Consent Form for Participation in a Research Study** 

**Study Title**: The **ASPIRE Study** (**A**ortic **S**tenosis: **P**references **I**n T**RE**atment)

You are being asked for your consent to take part in a research study. This paragraph provides a summary of this research. It describes the key information that we believe most people need in order to decide whether to take part in this research.  We include this form due to regulations regarding what all studies are required to include.

**What should I know about this research?**

- Participants are limited to people 18 years or older with a history of symptomatic aortic stenosis.
- Taking part in this research is voluntary. Whether you take part is up to you. If you don’t take part, it won’t be held against you.
- This study is sponsored by Shared Decision Making Resources under a Scientific Research Agreement with Edwards Lifesciences (https://www.edwards.com/).
- You will be invited to a conference call with a member of the study team who will share a computer screen online with you. You will be shown one or more questions asking about treatment goals or preferences for treatment. You will be asked for suggestions to help us refine the question.
- This interview may be audio recorded (with your permission) and will last about 15 to 30 minutes.

**Why is this research being done?**
Decisions about aortic stenosis are difficult. This study hopes to better understand what's important to people in making treatment decisions for aortic stenosis. It also aims to help newly diagnosed patients make decisions about treatment and help health care providers learn how patients make their decisions.

**How long will I be in this research?**We expect that taking part in this research will take 15-30 minutes total.

**What happens to me if I agree to take part in this research?**

- We will schedule a conference call with a member of the study team who will share a computer screen online with you.
- We will schedule the call at your convenience.
- We will ask you what you think the question is asking, how you might answer it, and how you might make the question easier to understand.
- We may address sensitive topics such as aortic stenosis symptoms or personal values that come into play when making a health-related decision.

**Could being in this research hurt me or pose a risk?**You may feel emotionally uneasy when asked to think about AS. There is a risk of loss of confidentiality through data entered online or audio-recorded. To the best of our ability, all of the information you give us will remain completely confidential. We take many steps to minimize the risk of losing confidentiality by encrypting data, using the most secure software, removing personal identifiers from stored data, and restricting access to the data to only the study investigators. There is also the possible inconvenience from taking the time to complete the study.

**Who will have access to my study information?**
Records of your participation in this study will be held confidential so far as permitted by law. However, the study principal investigator and, under certain circumstances, New England Independent Review Board (IRB) will be able to inspect and have access to confidential data that identifies you by name. Any publication or presentation of the data will not identify you.

**Will being in this research benefit me?**There are no direct benefits to participants. However, your participation may help us learn more about what matters to patients and improve decision-making tools about treating aortic stenosis.

**What other options are available?**
You may choose not to participate in this study without penalty.

**Will it cost me anything to participate in this study?**We do not expect that you will incur any expenses for this online activity. We do offer a small compensation for your effort (see below).

**Will I be paid for being in this study?**
As compensation for your participation, at the end of the session you will be given an Amazon gift card worth $20.00.

**Am I required to participate in this study?**
Your participation in this study is completely voluntary and you can withdraw at any time. Your refusal to participate or your withdrawal from the study will involve no penalty or loss to you. You may stop your participation at any time without affecting your ongoing medical care.

**Can I be removed from the study without my permission?**
We may end your participation in this study for any of the following reasons:

- If we are unable to schedule the activity;
- If the study is cancelled by the sponsor or the New England Independent Review Board; or
- For administrative reasons.

**Who do I contact if I have questions about the study?**
If you have questions or concerns about this study, you may contact Dr. Nananda Col at (207) 272-9829 or via email at ASPIRE[@SDMR.US.](mailto:GOALS@SDMR.US.) If you have questions about your rights as a research subject, or other concerns about this research, you can contact the New England Independent Review Board at 1-800-232- 9570 or at info@neirb.com.

**Statement of consent**
I have read the above consent form and agree to participate in this study and I am over 18 years of age.

- Yes
- No

### Appendix J2: In-person Small group meeting ICF

**Consent Form for Participation in a Research Study**

**Study Title:**The **ASPIRE Study** (**A**ortic **S**tenosis **P**references **I**n T**RE**atment)

You are being asked for your consent to take part in a research study. This paragraph provides a summary of this research. It describes the key information that we believe most people need in order to decide whether to take part in this research.  We include this form due to regulations regarding what all studies are required to include.

**What should I know about this research?**

- Participants are limited to people 18 years or older with a history of symptomatic aortic stenosis.
- Taking part in this research is voluntary. Whether you take part is up to you. If you don’t take part, it won’t be held against you.
- This study is sponsored by Shared Decision Making Resources under a Scientific Research Agreement with Edwards Lifesciences (https://www.edwards.com/).
- During this study, you will be invited to a small group meeting with 5-10 other people with aortic stenosis that will last 60-90 minutes. We will discuss factors that influenced your decision about treating your aortic stenosis. The meeting will take place at a nearby hotel or designated facility. The discussion may be audio recorded (with your permission) but no one will know your name. Refreshments will be served.

**Why is this research being done?**
Decisions about aortic stenosis are difficult. This study hopes to better understand what's important to people in making treatment decisions for aortic stenosis. It also aims to help newly diagnosed patients make decisions about treatment and help health care providers learn how patients make their decisions.

**How long will I be in this research?**
We expect that taking part in this research will take 60-90 minutes.

**What happens to me if I agree to take part in this research?**

- We will schedule a small group meeting at the convenience of participants.
- At the meeting, you will be shown a question about what was important to you when making decisions about choosing how to treat aortic stenosis.
- You will be asked to write down your responses to the question.
- We will then gather and combine responses from everyone in the room and ask you to rate and rank them according to their importance to you.
- We may discuss sensitive topics such as aortic stenosis symptoms or personal values that come into play when making a health-related decision.

**Could being in this research hurt me or pose a risk?**
You may feel emotionally uneasy when asked to think about aortic stenosis. There is a risk of losing privacy through comments you make during the meeting. There is a risk of loss of confidentiality through information entered online. There is also the possible inconvenience from taking the time to complete the study.

To the best of our ability, all of the information you give us will remain completely confidential. We take many steps to minimize the risk of losing confidentiality by encrypting data, using the most secure software, removing personal identifiers from stored data, and restricting access to the data to only the study investigators.

**Who will have access to my study information?**
Records of your participation in this study will be held confidential so far as permitted by law. However, the study principal investigator and, under certain circumstances, New England Independent Review Board (IRB) will be able to inspect and have access to confidential data that identifies you by name. Any publication or presentation of the data will not identify you.

**Will being in this research benefit me?**
There are no direct benefits to participants. However, your participation may help us learn more about what matters to patients and improve decision-making tools about treating aortic stenosis.

**What other options are available?**
You may choose not to participate in this study without penalty.

**Will it cost me anything to participate in this study?**
You may incur miscellaneous expenses such as travel or childcare costs for small group meetings. All attempts will be made to schedule meetings at a convenient location and time, since we will not be able to pay for any travel or child care costs. We do offer a small compensation for your effort (see below).

**Will I be paid for being in this study?**
As compensation for your participation, at the end of the session you will be given an Amazon gift card worth $50.00.

**Am I required to participate in this study?**
Your participation in this study is completely voluntary and you can withdraw at any time. Your refusal to participate or your withdrawal from the study will involve no penalty or loss to you. You may stop your participation at any time without affecting your ongoing medical care.

**Can I be removed from the study without my permission?**
We may end your participation in this study for any of the following reasons:

- If we are unable to schedule the activity;
- If the study is cancelled by the sponsor or the New England Independent Review Board; or
- For administrative reasons.

**Who do I contact if I have questions about the study?**
If you have questions or concerns about this study, you may contact Dr. Nananda Col at (207) 272-9829 or via email at ASPIRE[@SDMR.US.](mailto:GOALS@SDMR.US.) If you have questions about your rights as a research subject, or other concerns about this research, you can contact the New England Independent Review Board at 1-800-232- 9570 or at info@neirb.com.

**Statement of consent**

I have read the above consent form and agree to participate in this study and I am over 18 years of age.

- Yes
- No

### Appendix J3: Online NGT ICF

Consent Form for Participation in a Research Study 

Study Title: The **ASPIRE Study** (**A**ortic **S**tenosis **P**references **I**n T**RE**atment)

You are being asked for your consent to take part in a research study. This paragraph provides a summary of this research. It describes the key information that we believe most people need in order to decide whether to take part in this research.  We include this form due to regulations regarding what all studies are required to include.

**What should I know about this research?**

- Participants are limited to people 18 years or older with a history of symptomatic aortic stenosis.
- Taking part in this research is voluntary. Whether you take part is up to you. If you don’t take part, it won’t be held against you.
- This study is sponsored by Shared Decision Making Resources under a Scientific Research Agreement with Edwards Lifesciences (https://www.edwards.com/).
- During this study, you will be invited to take part in an Online Forum with five to ten other (anonymous) people with aortic stenosis. Over the course of a few weeks, you will receive two online surveys that will give you specific instructions and questions to answer. You can answer the questions at your convenience.

**Why is this research being done?**
Decisions about aortic stenosis are difficult. This study hopes to better understand what's important to people in making treatment decisions for aortic stenosis. It also aims to help newly diagnosed patients make decisions about treatment and help health care providers learn how patients make their decisions.

**How long will I be in this research?**We expect that taking part in this research will take 60-90 minutes total, spread out over several weeks.

**What happens to me if I agree to take part in this research?**

- You will receive the online survey [after reading this consent form and agreeing to participate] [within the next few days].
- You will be asked to write down responses to a question about what’s important to you when making decisions about choosing how to treat aortic stenosis.
- You will be asked to review and rate other people’s responses to this question.
- We may address sensitive topics such as aortic stenosis symptoms or personal values that come into play when making a health-related decision.

**Could being in this research hurt me or pose a risk?**You may feel emotionally uneasy when asked to think about AS. There is a risk of loss of confidentiality through data entered online. To the best of our ability, all of the information you give us will remain completely confidential. We take many steps to minimize the risk of losing confidentiality by encrypting data, using the most secure software, removing personal identifiers from stored data, and restricting access to the data to only the study investigators. There is also the possible inconvenience from taking the time to complete the study.

**Who will have access to my study information?**
Records of your participation in this study will be held confidential so far as permitted by law. However, the study principal investigator and, under certain circumstances, New England Independent Review Board (IRB) will be able to inspect and have access to confidential data that identifies you by name. Any publication or presentation of the data will not identify you.

**Will being in this research benefit me?**There are no direct benefits to participants. However, your participation may help us learn more about what matters to patients and improve decision-making tools about treating aortic stenosis.

**What other options are available?**
You may choose not to participate in this study without penalty.

**Will it cost me anything to participate in this study?**We do not expect that you will incur any expenses for this online activity. We do offer a small compensation for your effort (see below).

**Will I be paid for being in this study?**
As compensation for your participation, at the end of the session you will be given an Amazon gift card worth $50.00.

**Am I required to participate in this study?**
Your participation in this study is completely voluntary and you can withdraw at any time. Your refusal to participate or your withdrawal from the study will involve no penalty or loss to you. You may stop your participation at any time without affecting your ongoing medical care.

**Can I be removed from the study without my permission?**
We may end your participation in this study for any of the following reasons:

- If we are unable to schedule the activity;
- If the study is cancelled by the sponsor or the New England Independent Review Board; or
- For administrative reasons.

**Who do I contact if I have questions about the study?**
If you have questions or concerns about this study, you may contact Dr. Nananda Col at (207) 272-9829 or via email at ASPIRE[@SDMR.US.](mailto:GOALS@SDMR.US.) If you have questions about your rights as a research subject, or other concerns about this research, you can contact the New England Independent Review Board at 1-800-232- 9570 or at info@neirb.com.

**Statement of consent**

I have read the above consent form and agree to participate in this study and I am over 18 years of age.

- Yes
- No

### Appendix J4: Card Sort ICF

**Consent Form for Participation in a Research Study** 

**Study Title:** The **ASPIRE Study** (**A**ortic **S**tenosis **P**references **I**n T**RE**atment)

You are being asked for your consent to take part in a research study. This paragraph provides a summary of this research. It describes the key information that we believe most people need in order to decide whether to take part in this research.  We include this form due to regulations regarding what all studies are required to include.

**What should I know about this research?**

- Participants are limited to people 18 years or older with a history of symptomatic aortic stenosis.
- Taking part in this research is voluntary. Whether you take part is up to you. If you don’t take part, it won’t be held against you.
- This study is sponsored by Shared Decision Making Resources under a Scientific Research Agreement with Edwards Lifesciences (https://www.edwards.com/).
- During this study, you will be invited to take one online survey that will ask you to rate and group together items that are important to people making decisions about treating aortic stenosis. You can answer the questions at your convenience.

**Why is this research being done?**
Decisions about aortic stenosis are difficult. This study hopes to better understand what's important to people in making treatment decisions for aortic stenosis. It also aims to help newly diagnosed patients make decisions about treatment and help health care providers learn how patients make their decisions.

**How long will I be in this research?**
We expect that taking part in this research will take 20-30 minutes.

**What happens to me if I agree to take part in this research?**

- You will be asked to review and rate a list of items that are important to people making decisions about treating aortic stenosis.
- You will be asked to group items together that seem similar to you.
- We will ask you to try to come up with a name for the groups.
- The items may address sensitive topics such as aortic stenosis symptoms or personal values that come into play when making a health-related decision.

**Could being in this research hurt me or pose a risk?**
You may feel emotionally uneasy when asked to think about AS. There is a risk of loss of confidentiality through data entered online. To the best of our ability, all of the information you give us will remain completely confidential. We take many steps to minimize the risk of losing confidentiality by encrypting data, using the most secure software, removing personal identifiers from stored data, and restricting access to the data to only the study investigators. There is also the possible inconvenience from taking the time to complete the study.

**Who will have access to my study information?**
Records of your participation in this study will be held confidential so far as permitted by law. However, the study principal investigator and, under certain circumstances, New England Independent Review Board (IRB) will be able to inspect and have access to confidential data that identifies you by name. Any publication or presentation of the data will not identify you.

**Will being in this research benefit me?**
There are no direct benefits to participants. However, your participation may help us learn more about what matters to patients and improve decision-making tools about treating aortic stenosis.

**What other options are available?**You may choose not to participate in this study without penalty.

**Will it cost me anything to participate in this study?**
We do not expect that you will incur any expenses for this online activity. We do offer a small compensation for your effort (see below).

**Will I be paid for being in this study?**
As compensation for your participation, at the end of the session you will be given an Amazon gift card worth $35.00.

**Am I required to participate in this study?**
Your participation in this study is completely voluntary and you can withdraw at any time. Your refusal to participate or your withdrawal from the study will involve no penalty or loss to you. You may stop your participation at any time without affecting your ongoing medical care.

**Can I be removed from the study without my permission?**
We may end your participation in this study for any of the following reasons:

- If we are unable to schedule the activity;
- If the study is cancelled by the sponsor or the New England Independent Review Board; or
- For administrative reasons.

**Who do I contact if I have questions about the study?**
If you have questions or concerns about this study, you may contact Dr. Nananda Col at (207) 272-9829 or via email at ASPIRE[@SDMR.US.](mailto:GOALS@SDMR.US.) If you have questions about your rights as a research subject, or other concerns about this research, you can contact the New England Independent Review Board at 1-800-232- 9570 or at info@neirb.com.

**Statement of consent**

I have read the above consent form and agree to participate in this study and I am over 18 years of age.

- Yes
- No

### Appendix J5: Usability Testing ICF

**Consent Form for Participation in a Research Study** 
**Study Title**: **ASPIRE: A**ortic **S**tenosis **P**reference **I**ntegration **RE**source

You are being asked for your consent to take part in a research study. This paragraph provides a summary of this research. It describes the key information that we believe most people need in order to decide whether to take part in this research.  We include this form due to regulations regarding what all studies are required to include.

**What should I know about this research?**

- Participants are limited to people 18 years or older with a history of symptomatic aortic stenosis.
- Taking part in this research is voluntary. Whether you take part is up to you. If you don’t take part, it won’t be held against you.
- This study is sponsored by Shared Decision Making Resources under a Scientific Research Agreement with Edwards Lifesciences (https://www.edwards.com/).
- You will be invited to a conference call with a member of the study team who will share a computer screen online with you. You will be shown all or part of an online tool designed to help people with aortic stenosis make treatment decisions. You will be asked for your feedback about the tool, including how it looked, how you felt about the information shown, how you might navigate the tool, and what changes you might suggest. This interview will be audio recorded and will last about 30-60 minutes.

**Why is this research being done?**
Decisions about aortic stenosis are difficult. This study hopes to better understand what's important to people in making treatment decisions for aortic stenosis. It also aims to help newly diagnosed patients make decisions about treatment and help health care providers learn how patients make their decisions.

**How long will I be in this research?**We expect that taking part in this research will take 30-60 minutes total.

**What happens to me if I agree to take part in this research?**

- We will schedule a conference call over the next few [days][weeks] at your convenience where you and a member of the study team can share a computer screen online.
- You will be asked to review the online preference assessment tool, "thinking aloud" as you do so. You will be asked to rate your impressions of the tool and for suggestions to improve it.
- We may address sensitive topics such as aortic stenosis symptoms or personal values that come into play when making a health-related decision.

**Could being in this research hurt me or pose a risk?**You may feel emotionally uneasy when asked to think about AS. There is a risk of loss of confidentiality through data entered online or audio-recorded. To the best of our ability, all of the information you give us will remain completely confidential. We take many steps to minimize the risk of losing confidentiality by encrypting data, using the most secure software, removing personal identifiers from stored data, and restricting access to the data to only the study investigators. There is also the possible inconvenience from taking the time to complete the study.

**Who will have access to my study information?**
Records of your participation in this study will be held confidential so far as permitted by law. However, the study principal investigator and, under certain circumstances, New England Independent Review Board (IRB) will be able to inspect and have access to confidential data that identifies you by name. Any publication or presentation of the data will not identify you.

**Will being in this research benefit me?**There are no direct benefits to participants. However, your participation may help us learn more about what matters to patients and improve decision-making tools about treating aortic stenosis.

**What other options are available?**
You may choose not to participate in this study without penalty.

**Will it cost me anything to participate in this study?**We do not expect that you will incur any expenses for this online activity. We do offer a small compensation for your effort (see below).

**Will I be paid for being in this study?**
As compensation for your participation, at the end of the session you will be given an Amazon gift card worth $50.00.

**Am I required to participate in this study?**
Your participation in this study is completely voluntary and you can withdraw at any time. Your refusal to participate or your withdrawal from the study will involve no penalty or loss to you. You may stop your participation at any time without affecting your ongoing medical care.

**Can I be removed from the study without my permission?**
We may end your participation in this study for any of the following reasons:

- If we are unable to schedule the activity;
- If the study is cancelled by the sponsor or the New England Independent Review Board; or
- For administrative reasons.

**Who do I contact if I have questions about the study?**
If you have questions or concerns about this study, you may contact Dr. Nananda Col at (207) 272-9829 or via email at ASPIRE[@SDMR.US.](mailto:GOALS@SDMR.US.) If you have questions about your rights as a research subject, or other concerns about this research, you can contact the New England Independent Review Board at 1-800-232- 9570 or at info@neirb.com.

**Statement of consent**
I have read the above consent form and agree to participate in this study and I am over 18 years of age.

- Yes
- No

### Appendix J6: Pilot Study: Patient ICF (Phase 5)

**Consent Form for Participation in a Research Study**

**Study Title**: The AVITA Study

**DETAILED RESEARCH CONSENT**

You are being asked for your consent to take part in a research study. The following section provides a summary of this study; it describes the key information that we believe most people need to decide whether to take part in this project.

**What should I know about this research?**

- Participants are limited to about 25 adults with aortic stenosis who are considering valve replacement and have an upcoming appointment to discuss treatment options for aortic stenosis.
- Participants will be asked to view an online learning tool about aortic stenosis (called AVITA) and to complete 2 online surveys.
- Taking part in this research is voluntary. There are no consequences if you choose not to participate or decide to participate now and later drop out.
- The website, Valvesurvey.com, describes the study in more detail.
- You are invited to ask all your questions before you decide whether you want to participate. Contact information is listed below.

**Why is this research being done?**

- Choosing treatment for aortic stenosis can be difficult. We are testing an online learning tool called AVITA (**A**ortic **V**alve **i**n **T**reatment **A**pproaches) that was designed to help people explore their treatment options for aortic stenosis.

**How long will I be in this research?**

You will be enrolled in this study until you complete the final survey, which will be emailed to you shortly after your upcoming heart team appointment.

**What happens to me if I agree to take part in this research?**

- You will be asked to complete 2 online surveys and to view an online learning tool.
- The first survey follows this consent form and should take about 5-10 minutes.
- Then you will be connected (automatically) to the AVITA learning tool, which takes about 15-20 minutes to review.
- AVITA asks about your treatment goals and preferences. It gives you personalized feedback as well as a printable summary of your goals and preferences that you can share with your heart team.
- AVITA will email your summary to you and securely email or fax it to your designated heart team clinician(s). You can also print it and bring it with you to the appointment. The study team can also print and mail it to you.
- Shortly after your heart team appointment, you will be emailed another survey asking about your treatment decision. That survey should take 10-15 minutes to complete.
- Information regarding your final treatment choice will be shared with the study team.
- All study surveys are online. You can contact the research team for help with any surveys.

**What are my responsibilities if I take part in this research?**

If you take part in this research, you will be asked to review the AVITA online learning tool, to consider discussing the summary report with your designated heart team clinician, and to complete 2 online surveys.

**Could being in this research hurt me?**

You may feel emotionally uneasy when asked to think about treatment options for aortic stenosis. To the best of our ability, all the information you give us will remain completely confidential but there is a risk of loss of confidentiality through data entered online. We take many steps to minimize the risk of losing confidentiality by encrypting data, using secure software, separating personal identifiers from stored data, and restricting access to the data only to the study team.

**Will it cost me money to take part in this research?**

We do not expect that you will incur any expenses while completing these online surveys. We do, however, offer a small compensation for your effort (see below).

**Will I be paid for taking part in this research?**

You will be paid for your time and effort with online Amazon gift cards or equivalent (total of $75 for all study activities). The first payment of $40 will be sent after completing this survey and the AVITA tool. The next (final) payment of $35 will be sent after completing the survey after your heart team appointment.

**Will being in this research benefit me?**

There are no direct benefits to participants other than possibly learning more about your treatment options. However, your participation may help us improve decision-making tools about treating aortic stenosis.

**What other choices do I have besides taking part in this research?**

Your alternative is to not take part in the research.

**What happens to the information collected for this research?**

Records of your participation in this study will be held confidential so far as permitted by law. However, the study principal investigator and, under certain circumstances, WCG Independent Review Board (the name of the oversight organization) will be able to inspect and have access to confidential data that identifies you by name. Any publication or presentation of the data will not identify you.

**What if I am injured because of taking part in this research?**

We do not expect that you will be hurt by taking part in this research study. No funds have been set aside for payments or other forms of compensation (such as for lost wages, lost time, or discomfort) other than compensation for your participation (see above). However, you do not give up your legal rights by signing this consent form.

**Can I be removed from this research without my approval?**

We will tell you about any new information that may affect your health, welfare, or choice to stay in this research. We may end your participation in this study for any of the following reasons:

- If the study is cancelled by the sponsor or the WCG Independent Review Board (IRB); or
- For administrative reasons.

**Who can answer my questions about this research?**

Please direct any questions to the research team at info@valvesurvey.com or to Dr. Nananda Col at (207) 272-9829.    
If you have any concerns about your rights as a study participant or the way that you have been treated, talk to the research team at the phone number listed above. This research is being overseen by WCG IRB. An IRB is a group of people who perform independent review of research studies. You may talk to them at 855-818-2289 or researchquestions@wcgirb.com if:

- You have questions, concerns, or complaints that are not being answered by the research team.
- You cannot reach the research team.
- You want to talk to someone else about the research.
- You have questions about your rights as a research participant.

**What happens if I agree to be in this research, but change my mind later?**

If you decide to leave this research study, please contact the research team (info@valvesurvey.com) so that we can remove you from the study contact list and provide follow up information if needed.

**Statement of Consent:**

I have read the above consent form and agree to participate in this study, and I am over 18 years of age.

- Yes
- No

### Appendix J7: Pilot Study HCP ICF (Phase 5)

**RESEARCH PARTICIPANT CONSENT FORM**
Title:  The AVITA Pilot Study
Protocol No.:     HCP-8243002
Sponsor:           Shared Decision Making Resources under a Scientific Research Agreement with Edwards Lifesciences
Investigator:      Nananda Col, MD, MPH, MPP, FACP
                1119 Five Islands Road
                Georgetown, ME 04548
                United States (USA)
Study-Related
Phone Number:           1 (207) 272-9829 (Nananda Col)
                              
**DETAILED RESEARCH CONSENT**
**What should I know about this research?**

- Taking part in this research is voluntary. There are no consequences if you choose not to participate.
- The shared decision making tool, AVITA, creates a “snapshot” summary of your patient’s goals and preferences and shares it with their heart team to facilitate shared decision making.
- Whether or not you received or reviewed the snapshot summary, we ask you to participate.
- Participation involves completing a brief online survey, which should take about 2-5 minutes.
- You are invited to ask all of your questions before you decide whether you want to participate. Contact information is listed below.
- About 25 patients with severe aortic stenosis and who have an upcoming appointment (within 4 weeks) and their heart team clinicians will be invited to participate in this study.
- The website, Valvesurvey.com, describes the study in more detail.

**Why is this research being done?**

- We are testing an online shared decision making tool called AVITA, designed to help people with severe aortic stenosis explore their treatment options.
- We want to find out if AVITA facilitates shared decision making.

**How long will I be in this research?**

- Your participation in this research will last for the 2-5 minutes it takes to complete this evaluation. If you have other patients participating in the study, you will be asked to consent and complete another brief evaluation.

**What happens to me if I agree to take part in this research?**

- You will be asked a few questions (online) about yourself and the clinic visit with your patient who is in the study. This should take just a few minutes.

**What are my responsibilities if I take part in this research?**

- To complete a brief survey, which follows this page.

**Could being in this research hurt me?**

- We do not anticipate that this research can hurt you.
- To the best of our ability, all of the information you give us will remain completely confidential but there is a risk of loss of confidentiality through data entered online or audio recorded. We take many steps to minimize the risk of losing confidentiality by encrypting data, using secure software, separating personal identifiers from stored data, and restricting access to the data only to the study team.

**Will it cost me money to take part in this research?**

- We do not expect that you will incur any expenses while completing these online surveys. We do, however, offer a small compensation for your effort (see below).

**Will being in this research benefit me?**

- There are no direct benefits to participants other than the possibility that the AVITA tool may improve the conversation you have with your patient about treatment. However, your participation may help us learn more about what matters to patients and improve decision-making tools for aortic stenosis.

**What other choices do I have besides taking part in this research?**

- You may choose to not participate in this study without penalty.

**What happens to the information collected for this research?**

- Records of your participation in this study will be held confidential so far as permitted by law. However, the study principal investigator and, under certain circumstances, WCG Independent Review Board (wcgirb.com, the oversight organization) will be able to inspect and have access to confidential data that identifies you by name. Any publication or presentation of the data will not identify you.

**Who can answer my questions about this research?**

- Please direct any questions to the research team at info@valvesurvey.com or to Dr. Nananda Col at (207) 272-9829 or Dr. Megan Coylewright at Megan.coylewright@erlanger.org.
- If you have any concerns about your rights as a study participant or the way that you have been treated, talk to the research team at the phone number listed above. This research is being overseen by WCG IRB. You may talk to them at 855-818-2289 or researchquestions@wcgirb.com if:

●   You have questions, concerns, or complaints that are not being answered by the research team.
●   You are not getting answers from the research team.
●   You cannot reach the research team.
●   You want to talk to someone else about the research.
●   You have questions about your rights as a research participant.

**What if I am injured because of taking part in this research?**

- We do not expect that you will be hurt by taking part in this research study. No funds have been set aside for payments or other forms of compensation (such as for lost wages, lost time, or discomfort) other than compensation for your participation (see below). However, you do not give up your legal rights by signing this consent form.

**Can I be removed from this research without my approval?**

- We will tell you about any new information that may affect your choice to participate in this research. We may end your participation in this study for any of the following reasons:
- If the study is cancelled by the sponsor or the WCG IRB; or
- For administrative reasons.

**What happens if I agree to be in this research, but I change my mind later?**

- If you decide to leave this research study, please contact the research team (info@valvesurvey.com) so that we can remove you from the study contact list and provide follow-up information if needed.

**Will I be paid for taking part in this research?**

- As compensation for your participation, you will be emailed an Amazon gift card worth $75.

**Statement of Consent:**
I have read the above consent form and agree to participate in this study and I am over 18 years of age.

- Yes, I agree
- No, I do not agree

## Appendix K: Pilot Study AVITA Preference Assessment Tool (Phase 5)

Start of Block: INTRO SCREEN and TERMS

Q1.1     Your doctor or healthcare provider sent you this brief questionnaire to help you choose treatment for your aortic stenosis and to help your treatment team understand what is important to you. The questionnaire will take around **10-15 minutes**.   **Aortic stenosis** narrows the aortic heart valve, making it harder for blood to get to the rest of the body.
   **Effective treatments are available. Choices** include valve replacement (either surgical or less invasive) or medications. Nearly everyone can be treated.
   **The best treatment** depends on what matters to you and your goals.

 Your responses will be used to create a **printable summary** to share with your healthcare provider. **Sharing** what matters to you with your healthcare provider can make your voice heard, help find the best treatment, and get your questions answered.
   **Instructions on use**: To move to the next page, click on the **NEXT** button in the bottom right corner of each page. To go back to an earlier page, click on the **BACK** button in the bottom left of the page. Not all sections allow you to go back.   Click **NEXT** to continue.   *Last updated 11/29/2021*

| Page Break |  |
| --- | --- |

Display This Question:

If Contact List FirstName Is Empty

Or Contact List LastName Is Empty

Or Contact List Email1 Is Empty

Q1.2 Please enter your name and email.

Display This Choice:

If Contact List FirstName Is Empty

- First Name ________________________________________________

Display This Choice:

If Contact List LastName Is Empty

- Last Name ________________________________________________

Display This Choice:

If Contact List Email1 Is Empty

- Email ________________________________________________

| Page Break |  |
| --- | --- |

| 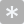 |
| --- |

Q183 What is the zip code for your primary residence where you receive care for your aortic stenosis?

________________________________________________________________

End of Block: INTRO SCREEN and TERMS

Start of Block: Decision role

| 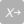 |
| --- |

Q2.1
  **Part 1. How to make the decision?**
 
**Let's Get Started!**        
Everyone deserves the quality of life that they desire. It is important to know that there are options in how you choose to treat your aortic stenosis. The decision should be made in partnership with you and your care team. These are your choices:


    *Aortic Valve Replacement can fix a valve that is not working well, improve quality of life, and prevent further decline in your health.*
 
**TAVR** (Transcatheter aortic valve replacement) is a less invasive procedure. Using local anesthesia, a small tube is placed in an artery, typically in the groin/leg* region, and threaded into the heart where the new valve is placed, pushing the old valve aside. Only tissue valves are used in TAVR.

 **SAVR** (Surgical aortic valve replacement) involves open-chest surgery under general anesthesia. The new valve can be mechanical or tissue (from human or animal tissue). Mechanical valves last longer than tissue valves but require blood thinners.
  
**No valve replacement (medications and/or comfort care**). Not replacing your valve means that your aortic stenosis will get worse. No medicines can fix the valve but there are medicines that can ease your symptoms. If you don't replace your valve, you will experience further decline, including chest pain, shortness of breath, passing out and a high likelihood of dying from this disease. 

  
How would you like to make a decision about treating your aortic stenosis? (please choose one)

- Make the final selection myself about which treatment I will receive.
- Make the final selection myself after seriously considering healthcare providers' opinions.
- Share responsibility with healthcare providers when deciding what treatment is best for me.
- Have healthcare providers make the final decision after considering my opinion.
- Leave all decisions regarding treatment to healthcare providers.

Display This Question:

If Part 1. How to make the decision? Let's get started! = Leave all decisions regarding treatment to healthcare providers.

Q2.3
 
**How to make the decision?** 
 
It can be tempting to leave all decisions to a trusted healthcare provider. 
 
But the treatment you choose will affect your life in so many ways.
Each treatment has its own benefits and risks. Knowing what you want and which risks you can accept will help you and your healthcare provider find the best treatment.
  It is best to discuss options with a heart team, which includes both a cardiac surgeon and a cardiologist who specializes in performing TAVR (this specialist is called an "interventional cardiologist"). Only cardiac surgeons perform SAVR. Interventional cardiologists and cardiac surgeons perform TAVR. General cardiologists (MD, DO) and cardiology Nurse Practitioners (NP) and Physician Assistants (PA) diagnose valve problems but do not perform heart valve procedures like SAVR or TAVR.
  Healthcare providers are experts in medicine, but you're the only expert on *you*.  You don't have to learn everything about aortic stenosis or valve replacement. You just have to know what's important to you.
  Answering the following questions will help you tell your healthcare providers what matters to you. If you don't tell your healthcare providers, how will they know?

 If you would like to change your response to this question, please select the **BACK** button.

Display This Question:

If Part 1. How to make the decision? Let's get started! = Make the final selection myself after seriously considering healthcare providers' opinions.

Or Part 1. How to make the decision? Let's get started! = Share responsibility with healthcare providers when deciding what treatment is best for me.

Or Part 1. How to make the decision? Let's get started! = Have healthcare providers make the final decision after considering my opinion.

Q2.4
 
**How to make the decision?**
 
Being an active part of the treatment team is one of the most important things you can do to find the best treatment.

 **Healthcare providers are experts in medicine, but you're the only expert on *you*.**

 Your heart team includes your general cardiologist, a cardiac surgeon and a cardiologist who specializes in TAVR (this specialist is called an "interventional cardiologist"). SAVR is only done by cardiac surgeons. TAVR is done by interventional cardiologists or cardiac surgeons. General cardiologists (MD, DO) and cardiology Nurse Practitioners (NP) and Physician Assistants (PA) diagnose valve problems but do not perform heart valve procedures like SAVR or TAVR.   Answering the following questions will help you share your preferences with your healthcare providers.

Display This Question:

If Part 1. How to make the decision? Let's get started! = Make the final selection myself about which treatment I will receive.

Q2.5
  **How to make the decision?**

 Having trusted medical professionals involved in decision-making can help you arrive at the best treatment. Medical care has become so complex that it is difficult for any one person to be aware of the latest treatment options and developments.

 If there are barriers that keep you from reaching out to your doctor, connect with your nurse or another health professional for support.

 Your heart team knows all about the latest treatment options. This team includes both a cardiac surgeon and a cardiologist who specializes in performing TAVR (this specialist is called an "interventional cardiologist"). Only cardiac surgeons perform SAVR. Interventional cardiologists and cardiac surgeons perform TAVR. General cardiologists (MD, DO) and cardiology Nurse Practitioners (NP) and Physician Assistants (PA) diagnose valve problems but do not perform heart valve procedures like SAVR or TAVR.
 Answering the following questions will help you tell your healthcare providers what matters to you. If you don't tell your healthcare providers, how will they know?

 To change your response to the question, select the **BACK** button.

End of Block: Decision role

Start of Block: Treatment Goals:Outcomes

| 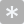 | 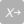 | 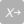 |
| --- | --- | --- |

Q3.1   **Part 2.  Hopes and expectations**     Now let's think about what's important to you.   **What do you hope for or expect will happen after treating your aortic stenosis?** Please choose the **3 most important items** from the list **below**.

 *(The list below came from real patients with aortic stenosis who experienced treatment first-hand.)*

- To be **independent** or active
- To lead a **long life**
- To reduce **fear of heart failure** and future risks
- To improve my **health, breathing, and quality of life**
- To feel comfortable about my **medications** and **future** plans
- To spend time with **family** and give them **peace of mind**
- To have a **less invasive** procedure, shorter **recovery**, and **support** services.

Display This Question:

If If &nbsp; Part 2.&nbsp; Hopes and expectations Now let's think about what's important to you. &nbsp; What do you hope for or expect will happen after treating your aortic stenosis?&nbsp; Please c... q://QID183/SelectedChoicesCount Is Greater Than or Equal to 1

| 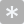 | 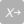 | 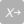 |
| --- | --- | --- |

Q3.3
 
**Hopes and expectations**

 Based upon your answers to the last question, here are more **hopes and expectations for treatment** for you to consider**.**
  
Please choose the **3 most important items** from the list **below**.

 To de-select (or remove) your response to an item, just click on it.

Display This Choice:

If   Part 2.  Hopes and expectations     Now let's think about what's important to you.   What do yo... = To reduce <strong>fear of heart failure</strong> and future risks

- To reduce fear of heart failure

Display This Choice:

If   Part 2.  Hopes and expectations     Now let's think about what's important to you.   What do yo... = To lead a <strong>long life</strong>

- To lead a long life

Display This Choice:

If   Part 2.  Hopes and expectations     Now let's think about what's important to you.   What do yo... = To reduce <strong>fear of heart failure</strong> and future risks

- To find a long-lasting solution to avoid repeat treatment

Display This Choice:

If   Part 2.  Hopes and expectations     Now let's think about what's important to you.   What do yo... = To reduce <strong>fear of heart failure</strong> and future risks

- To choose a treatment that has fewer long-term risks

Display This Choice:

If   Part 2.  Hopes and expectations     Now let's think about what's important to you.   What do yo... = To improve my <strong>health, breathing, and quality of life</strong>

- To improve my health

Display This Choice:

If   Part 2.  Hopes and expectations     Now let's think about what's important to you.   What do yo... = To improve my <strong>health, breathing, and quality of life</strong>

- To improve my quality of life

Display This Choice:

If   Part 2.  Hopes and expectations     Now let's think about what's important to you.   What do yo... = To improve my <strong>health, breathing, and quality of life</strong>

- To have more energy, strength, and stamina

Display This Choice:

If   Part 2.  Hopes and expectations     Now let's think about what's important to you.   What do yo... = To improve my <strong>health, breathing, and quality of life</strong>

- To breathe without difficulty

Display This Choice:

If   Part 2.  Hopes and expectations     Now let's think about what's important to you.   What do yo... = To feel comfortable about my <strong>medications </strong>and <strong>future </strong>plans

- To feel comfortable with the medications prescribed

Display This Choice:

If   Part 2.  Hopes and expectations     Now let's think about what's important to you.   What do yo... = To feel comfortable about my <strong>medications </strong>and <strong>future </strong>plans

- To be able to make realistic plans for the rest of my life

Display This Choice:

If   Part 2.  Hopes and expectations     Now let's think about what's important to you.   What do yo... = To be <strong>independent </strong>or active

- To be independent

Display This Choice:

If   Part 2.  Hopes and expectations     Now let's think about what's important to you.   What do yo... = To be <strong>independent </strong>or active

- To be able to do my normal activities

Display This Choice:

If   Part 2.  Hopes and expectations     Now let's think about what's important to you.   What do yo... = To be <strong>independent </strong>or active

- To be physically active

Display This Choice:

If   Part 2.  Hopes and expectations     Now let's think about what's important to you.   What do yo... = To be <strong>independent </strong>or active

- To lead an active lifestyle

Display This Choice:

If   Part 2.  Hopes and expectations     Now let's think about what's important to you.   What do yo... = To be <strong>independent </strong>or active

- To be able to travel

Display This Choice:

If   Part 2.  Hopes and expectations     Now let's think about what's important to you.   What do yo... = To be <strong>independent </strong>or active

- To be able to work

Display This Choice:

If   Part 2.  Hopes and expectations     Now let's think about what's important to you.   What do yo... = To spend time with <strong>family </strong>and give them <strong>peace of mind</strong>

- To spend time with family and friends

Display This Choice:

If   Part 2.  Hopes and expectations     Now let's think about what's important to you.   What do yo... = To spend time with <strong>family </strong>and give them <strong>peace of mind</strong>

- To give my family peace of mind

Display This Choice:

If   Part 2.  Hopes and expectations     Now let's think about what's important to you.   What do yo... = To have a <strong>less invasive</strong> procedure, shorter <strong>recovery</strong>, and <strong>support </strong>services.

- To avoid open heart surgery

Display This Choice:

If   Part 2.  Hopes and expectations     Now let's think about what's important to you.   What do yo... = To have a <strong>less invasive</strong> procedure, shorter <strong>recovery</strong>, and <strong>support </strong>services.

- To ensure that support services are in place

Display This Choice:

If   Part 2.  Hopes and expectations     Now let's think about what's important to you.   What do yo... = To have a <strong>less invasive</strong> procedure, shorter <strong>recovery</strong>, and <strong>support </strong>services.

- To know what to expect for recovery.

Display This Choice:

If   Part 2.  Hopes and expectations     Now let's think about what's important to you.   What do yo... = To have a <strong>less invasive</strong> procedure, shorter <strong>recovery</strong>, and <strong>support </strong>services.

- To minimize the length of recovery

Display This Choice:

If   Part 2.  Hopes and expectations     Now let's think about what's important to you.   What do yo... = To have a <strong>less invasive</strong> procedure, shorter <strong>recovery</strong>, and <strong>support </strong>services.

- To avoid general anesthesia

Display This Question:

If If   Hopes and expectationsBased upon your answers to the last question, here are more hopes and exp... q://QID184/SelectedChoicesCount Is Greater Than or Equal to 2

Q3.5 **Hopes and expectations**

 Here are the **hopes and expectations for treatment** that you just selected.
 
Please **rank** these items so that #1 is most important**,** #2 is your next most important, and # 3 is your third most important.
  
  If you are using a mobile device, you will see arrows that you can **tap** on to moves the questions up or down.

Display This Choice:

If   Hopes and expectationsBased upon your answers to the last question, here are more hopes and exp... = To reduce fear of heart failure

Display This Answer:

If If Hopes and expectationsBased upon your answers to the last question, here are more hopes and expec... q://QID184/SelectedChoicesCount Is Greater Than or Equal to 1

Display This Answer:

If If Hopes and expectationsBased upon your answers to the last question, here are more hopes and expec... q://QID184/SelectedChoicesCount Is Greater Than or Equal to 2

Display This Answer:

If If Hopes and expectationsBased upon your answers to the last question, here are more hopes and expec... q://QID184/SelectedChoicesCount Is Greater Than or Equal to 3

Display This Answer:

If If Hopes and expectationsBased upon your answers to the last question, here are more hopes and expec... q://QID184/SelectedChoicesCount Is Greater Than or Equal to 4

Display This Answer:

If If Hopes and expectationsBased upon your answers to the last question, here are more hopes and expec... q://QID184/SelectedChoicesCount Is Greater Than or Equal to 5

Display This Choice:

If   Hopes and expectationsBased upon your answers to the last question, here are more hopes and exp... = To lead a long life

Display This Choice:

If   Hopes and expectationsBased upon your answers to the last question, here are more hopes and exp... = To find a long-lasting solution to avoid repeat treatment

Display This Choice:

If   Hopes and expectationsBased upon your answers to the last question, here are more hopes and exp... = To choose a treatment that has fewer long-term risks

Display This Choice:

If   Hopes and expectationsBased upon your answers to the last question, here are more hopes and exp... = To improve my health

Display This Choice:

If   Hopes and expectationsBased upon your answers to the last question, here are more hopes and exp... = To improve my quality of life

Display This Choice:

If   Hopes and expectationsBased upon your answers to the last question, here are more hopes and exp... = To have more energy, strength, and stamina

Display This Choice:

If   Hopes and expectationsBased upon your answers to the last question, here are more hopes and exp... = To breathe without difficulty

Display This Choice:

If   Hopes and expectationsBased upon your answers to the last question, here are more hopes and exp... = To feel comfortable with the medications prescribed

Display This Choice:

If   Hopes and expectationsBased upon your answers to the last question, here are more hopes and exp... = To be able to make realistic plans for the rest of my life

Display This Choice:

If   Hopes and expectationsBased upon your answers to the last question, here are more hopes and exp... = To be independent

Display This Choice:

If   Hopes and expectationsBased upon your answers to the last question, here are more hopes and exp... = To be able to do my normal activities

Display This Choice:

If   Hopes and expectationsBased upon your answers to the last question, here are more hopes and exp... = To be physically active

Display This Choice:

If   Hopes and expectationsBased upon your answers to the last question, here are more hopes and exp... = To lead an active lifestyle

Display This Choice:

If   Hopes and expectationsBased upon your answers to the last question, here are more hopes and exp... = To be able to travel

Display This Choice:

If   Hopes and expectationsBased upon your answers to the last question, here are more hopes and exp... = To be able to work

Display This Choice:

If   Hopes and expectationsBased upon your answers to the last question, here are more hopes and exp... = To spend time with family and friends

Display This Choice:

If   Hopes and expectationsBased upon your answers to the last question, here are more hopes and exp... = To give my family peace of mind

Display This Choice:

If   Hopes and expectationsBased upon your answers to the last question, here are more hopes and exp... = To avoid open heart surgery

Display This Choice:

If   Hopes and expectationsBased upon your answers to the last question, here are more hopes and exp... = To ensure that support services are in place

Display This Choice:

If   Hopes and expectationsBased upon your answers to the last question, here are more hopes and exp... = To minimize the length of recovery

Display This Choice:

If   Hopes and expectationsBased upon your answers to the last question, here are more hopes and exp... = To avoid general anesthesia

Display This Choice:

If   Hopes and expectationsBased upon your answers to the last question, here are more hopes and exp... = To know what to expect for recovery.

|  | Display This Answer:  If If Hopes and expectationsBased upon your answers to the last question, here are more hopes and expec... q://QID184/SelectedChoicesCount Is Greater Than or Equal to 1  #1 | Display This Answer:  If If Hopes and expectationsBased upon your answers to the last question, here are more hopes and expec... q://QID184/SelectedChoicesCount Is Greater Than or Equal to 2  #2 | Display This Answer:  If If Hopes and expectationsBased upon your answers to the last question, here are more hopes and expec... q://QID184/SelectedChoicesCount Is Greater Than or Equal to 3  #3 | Display This Answer:  If If Hopes and expectationsBased upon your answers to the last question, here are more hopes and expec... q://QID184/SelectedChoicesCount Is Greater Than or Equal to 4  #4 | Display This Answer:  If If Hopes and expectationsBased upon your answers to the last question, here are more hopes and expec... q://QID184/SelectedChoicesCount Is Greater Than or Equal to 5  #5 |
| --- | --- | --- | --- | --- | --- |
| Display This Choice:  If   Hopes and expectationsBased upon your answers to the last question, here are more hopes and exp... = To reduce fear of heart failure  ⊗${Q3.3/ChoiceDescription/3} |  |  |  |  |  |
| Display This Choice:  If   Hopes and expectationsBased upon your answers to the last question, here are more hopes and exp... = To lead a long life  ⊗${Q3.3/ChoiceDescription/91} |  |  |  |  |  |
| Display This Choice:  If   Hopes and expectationsBased upon your answers to the last question, here are more hopes and exp... = To find a long-lasting solution to avoid repeat treatment  ⊗${Q3.3/ChoiceDescription/67} |  |  |  |  |  |
| Display This Choice:  If   Hopes and expectationsBased upon your answers to the last question, here are more hopes and exp... = To choose a treatment that has fewer long-term risks  ⊗${Q3.3/ChoiceDescription/68} |  |  |  |  |  |
| Display This Choice:  If   Hopes and expectationsBased upon your answers to the last question, here are more hopes and exp... = To improve my health  ⊗${Q3.3/ChoiceDescription/69} |  |  |  |  |  |
| Display This Choice:  If   Hopes and expectationsBased upon your answers to the last question, here are more hopes and exp... = To improve my quality of life  ⊗${Q3.3/ChoiceDescription/70} |  |  |  |  |  |
| Display This Choice:  If   Hopes and expectationsBased upon your answers to the last question, here are more hopes and exp... = To have more energy, strength, and stamina  ⊗${Q3.3/ChoiceDescription/71} |  |  |  |  |  |
| Display This Choice:  If   Hopes and expectationsBased upon your answers to the last question, here are more hopes and exp... = To breathe without difficulty  ⊗${Q3.3/ChoiceDescription/72} |  |  |  |  |  |
| Display This Choice:  If   Hopes and expectationsBased upon your answers to the last question, here are more hopes and exp... = To feel comfortable with the medications prescribed  ⊗${Q3.3/ChoiceDescription/74} |  |  |  |  |  |
| Display This Choice:  If   Hopes and expectationsBased upon your answers to the last question, here are more hopes and exp... = To be able to make realistic plans for the rest of my life  ⊗${Q3.3/ChoiceDescription/75} |  |  |  |  |  |
| Display This Choice:  If   Hopes and expectationsBased upon your answers to the last question, here are more hopes and exp... = To be independent  ⊗${Q3.3/ChoiceDescription/76} |  |  |  |  |  |
| Display This Choice:  If   Hopes and expectationsBased upon your answers to the last question, here are more hopes and exp... = To be able to do my normal activities  ⊗${Q3.3/ChoiceDescription/77} |  |  |  |  |  |
| Display This Choice:  If   Hopes and expectationsBased upon your answers to the last question, here are more hopes and exp... = To be physically active  ⊗${Q3.3/ChoiceDescription/78} |  |  |  |  |  |
| Display This Choice:  If   Hopes and expectationsBased upon your answers to the last question, here are more hopes and exp... = To lead an active lifestyle  ⊗${Q3.3/ChoiceDescription/79} |  |  |  |  |  |
| Display This Choice:  If   Hopes and expectationsBased upon your answers to the last question, here are more hopes and exp... = To be able to travel  ⊗${Q3.3/ChoiceDescription/80} |  |  |  |  |  |
| Display This Choice:  If   Hopes and expectationsBased upon your answers to the last question, here are more hopes and exp... = To be able to work  ⊗${Q3.3/ChoiceDescription/81} |  |  |  |  |  |
| Display This Choice:  If   Hopes and expectationsBased upon your answers to the last question, here are more hopes and exp... = To spend time with family and friends  ⊗${Q3.3/ChoiceDescription/82} |  |  |  |  |  |
| Display This Choice:  If   Hopes and expectationsBased upon your answers to the last question, here are more hopes and exp... = To give my family peace of mind  ⊗${Q3.3/ChoiceDescription/83} |  |  |  |  |  |
| Display This Choice:  If   Hopes and expectationsBased upon your answers to the last question, here are more hopes and exp... = To avoid open heart surgery  ⊗${Q3.3/ChoiceDescription/84} |  |  |  |  |  |
| Display This Choice:  If   Hopes and expectationsBased upon your answers to the last question, here are more hopes and exp... = To ensure that support services are in place  ⊗${Q3.3/ChoiceDescription/85} |  |  |  |  |  |
| Display This Choice:  If   Hopes and expectationsBased upon your answers to the last question, here are more hopes and exp... = To minimize the length of recovery  ⊗${Q3.3/ChoiceDescription/86} |  |  |  |  |  |
| Display This Choice:  If   Hopes and expectationsBased upon your answers to the last question, here are more hopes and exp... = To avoid general anesthesia  ⊗${Q3.3/ChoiceDescription/87} |  |  |  |  |  |
| Display This Choice:  If   Hopes and expectationsBased upon your answers to the last question, here are more hopes and exp... = To know what to expect for recovery.  ${Q3.3/ChoiceDescription/93} |  |  |  |  |  |

| Page Break |  |
| --- | --- |

Display This Question:

If Hopes and expectationsHere are the hopes and expectations for treatment that you just selected.  ... [ #5] (Count) = 1

Q3.6 **Hopes and expectations**

 You identified the following **hopes and expectations** as being most important to you**:**

**Important Specific Goals** 
${Q3.5/AnswerDescription/1}  ${Q3.5/ChoiceGroup/SelectedChoicesForAnswer/1}
${Q3.5/AnswerDescription/2}  ${Q3.5/ChoiceGroup/SelectedChoicesForAnswer/2}
${Q3.5/AnswerDescription/3}  ${Q3.5/ChoiceGroup/SelectedChoicesForAnswer/3}
${Q3.5/AnswerDescription/4}  ${Q3.5/ChoiceGroup/SelectedChoicesForAnswer/4}
${Q3.5/AnswerDescription/5}  ${Q3.5/ChoiceGroup/SelectedChoicesForAnswer/5}

 **You can change your responses by using the BACK arrow.**
 
If there is something specifically important to you that isn't on the list (such as attending a special event or participating in a hobby), please write it below.

________________________________________________________________

Display This Question:

Q3.7 **Hopes and expectations**

 You identified the following **hopes and expectations** as being most important to you**:**

**Treatment Goals**
${Q3.5/AnswerDescription/1}  ${Q3.5/ChoiceGroup/SelectedChoicesForAnswer/1}
${Q3.5/AnswerDescription/2}  ${Q3.5/ChoiceGroup/SelectedChoicesForAnswer/2}
${Q3.5/AnswerDescription/3}  ${Q3.5/ChoiceGroup/SelectedChoicesForAnswer/3}
${Q3.5/AnswerDescription/4}  ${Q3.5/ChoiceGroup/SelectedChoicesForAnswer/4}
 
**You can change your responses by using the BACK arrow.**
 
If there is something specifically important to you that isn't on the list (such as attending a special event or participating in a hobby), please write it below. If not, just leave it blank.

________________________________________________________________

Display This Question:

If Hopes and expectationsHere are the hopes and expectations for treatment that you just selected.  ... [ #4] (Count) = 0

And Hopes and expectationsHere are the hopes and expectations for treatment that you just selected.  ... [ #5] (Count) = 0

Q3.8 **Hopes and expectations**
  
You identified the following **hopes and expectations** as being most important to you**:**  

**Important Treatment Goals**
${Q3.5/AnswerDescription/1}  ${Q3.5/ChoiceGroup/SelectedChoicesForAnswer/1}
${Q3.5/AnswerDescription/2}  ${Q3.5/ChoiceGroup/SelectedChoicesForAnswer/2}
${Q3.5/AnswerDescription/3}  ${Q3.5/ChoiceGroup/SelectedChoicesForAnswer/3}
 
**You can change your responses by using the BACK arrow.**
 
If there is something specifically important to you that isn't on the list (such as attending a special event or participating in a hobby), please write it below. If not, just leave it blank.

________________________________________________________________

End of Block: Treatment Goals:Outcomes

Start of Block: Process_Goals

Q4.1   **Part 3. Reaching a good decision**  
 Now let's think about **what you need to know or feel to help you choose your** **treatment**.  Please select the **3 most important items** from the list below.
 
At times it may seem like we are asking you the same question a different way. This is intentional. Thank you for your patience.
 
*(The list below came from real patients with aortic stenosis who experienced treatment first-hand.)*

- To have **good information** about my options
- To have **trust** in the doctor, medical team, and hospital
- To know **why I have aortic stenosis** and how that factors into the treatment decision
- To know what to **expect** (**cost**, **others' experiences**)
- To **feel confident** about the decision (**time** to reflect, manage **anxiety**, **ethics**)
- Knowing if and when to have a valve procedure (**risks**, **urgency**, and **COVID**)

Display This Question:

If If &nbsp; Part 3. Reaching a good decision &nbsp; Now let&#39;s think about what you need to know or feel to help you choose your treatment.&nbsp; Please select the 3 most important items from ... q://QID180/SelectedChoicesCount Is Greater Than 0

| 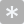 | 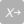 |
| --- | --- |

Q4.2
 
**Reaching a good decision**

 Based upon the answers you gave to the last question, here are more choices to consider. As a reminder, the question is:

 **What do you need to know or feel to help you choose your treatment?**
Please select the **3 most important items** from the list **below**.
 
You may need to scroll **down** to see all of the options.

Display This Choice:

If   Part 3. Reaching a good decision   Now let's think about what you need to know or feel to help... = To have <strong>trust </strong>in the doctor, medical team, and hospital

- To have a good medical team

Display This Choice:

If   Part 3. Reaching a good decision   Now let's think about what you need to know or feel to help... = To have <strong>trust </strong>in the doctor, medical team, and hospital

- To have trust in the doctor doing the procedure

Display This Choice:

If   Part 3. Reaching a good decision   Now let's think about what you need to know or feel to help... = To have <strong>trust </strong>in the doctor, medical team, and hospital

- Having good communication with my doctor and heart team

Display This Choice:

If   Part 3. Reaching a good decision   Now let's think about what you need to know or feel to help... = To have <strong>trust </strong>in the doctor, medical team, and hospital

- To know what my doctor recommends and why

Display This Choice:

If   Part 3. Reaching a good decision   Now let's think about what you need to know or feel to help... = To have <strong>trust </strong>in the doctor, medical team, and hospital

- The reputation and experience of the hospital doing the procedure on people like me

Display This Choice:

If   Part 3. Reaching a good decision   Now let's think about what you need to know or feel to help... = To have <strong>good information</strong> about my options

- To receive accurate information about treatments, recovery, and possible complications

Display This Choice:

If   Part 3. Reaching a good decision   Now let's think about what you need to know or feel to help... = To have <strong>good information</strong> about my options

- To be aware of available options

Display This Choice:

If   Part 3. Reaching a good decision   Now let's think about what you need to know or feel to help... = To<strong> feel confident </strong>about the decision (<strong>time </strong>to reflect, manage <strong>anxiety</strong>, <strong>ethics</strong>)

- To feel confident that I made the right decision

Display This Choice:

If   Part 3. Reaching a good decision   Now let's think about what you need to know or feel to help... = To know what to <strong>expect </strong>(<strong>cost</strong>,<strong> others' experiences</strong>)

- To be aware of the cost

Display This Choice:

If   Part 3. Reaching a good decision   Now let's think about what you need to know or feel to help... = To<strong> feel confident </strong>about the decision (<strong>time </strong>to reflect, manage <strong>anxiety</strong>, <strong>ethics</strong>)

- To choose an ethical treatment (avoid animal tissue valve)

Display This Choice:

If   Part 3. Reaching a good decision   Now let's think about what you need to know or feel to help... = To know <strong>why I have aortic stenosis</strong> and how that factors into the treatment decision

- To know how my age and health factors into the decisions

Display This Choice:

If   Part 3. Reaching a good decision   Now let's think about what you need to know or feel to help... = To know <strong>why I have aortic stenosis</strong> and how that factors into the treatment decision

- To know what caused my aortic valve stenosis.

Display This Choice:

If   Part 3. Reaching a good decision   Now let's think about what you need to know or feel to help... = To know <strong>why I have aortic stenosis</strong> and how that factors into the treatment decision

- Knowing if my aortic stenosis is hereditary.

Display This Choice:

If   Part 3. Reaching a good decision   Now let's think about what you need to know or feel to help... = Knowing if and when to have a valve procedure (<strong>risks</strong>, <strong>urgency</strong>, and <strong>COVID</strong>)

- Knowing the risks of not doing a valve replacement.

Display This Choice:

If   Part 3. Reaching a good decision   Now let's think about what you need to know or feel to help... = Knowing if and when to have a valve procedure (<strong>risks</strong>, <strong>urgency</strong>, and <strong>COVID</strong>)

- How soon should the procedure be done and why?

Display This Choice:

If   Part 3. Reaching a good decision   Now let's think about what you need to know or feel to help... = To<strong> feel confident </strong>about the decision (<strong>time </strong>to reflect, manage <strong>anxiety</strong>, <strong>ethics</strong>)

- To have enough time to reflect on my treatment decision and discuss it with my family.

Display This Choice:

If   Part 3. Reaching a good decision   Now let's think about what you need to know or feel to help... = Knowing if and when to have a valve procedure (<strong>risks</strong>, <strong>urgency</strong>, and <strong>COVID</strong>)

- Knowing the risks of postponing valve replacement during the COVID pandemic.

Display This Choice:

If   Part 3. Reaching a good decision   Now let's think about what you need to know or feel to help... = Knowing if and when to have a valve procedure (<strong>risks</strong>, <strong>urgency</strong>, and <strong>COVID</strong>)

- Knowing that I'm not placing myself at risk of getting COVID-19 or other infections.

Display This Choice:

If   Part 3. Reaching a good decision   Now let's think about what you need to know or feel to help... = To<strong> feel confident </strong>about the decision (<strong>time </strong>to reflect, manage <strong>anxiety</strong>, <strong>ethics</strong>)

- How do I ease my fear or anxiety about aortic stenosis and choosing treatment?

Q4.3
 **Reaching a good decision**

 Here is what you need to **know or feel to choose your treatment**, based on your answers to the last question.
  Please rank these items so that #1 is most important**,** #2 is next most important, and so on.

If you are using a mobile device, tapping on the arrows moves the questions up or down.
 

| Display This Choice:  If   Reaching a good decisionBased upon the answers you gave to the last question, here are more cho... = To feel confident that I made the right decision  ⊗${Q4.2/ChoiceDescription/73} |  |  |  |  |  |
| --- | --- | --- | --- | --- | --- |
| Display This Choice:  If   Reaching a good decisionBased upon the answers you gave to the last question, here are more cho... =  ⊗${Q4.2/ChoiceDescription/85} |  |  |  |  |  |

Display This Question:

If   Reaching a good decisionBased upon the answers you gave to the last question, here are more cho... = How do I ease my fear or anxiety about aortic stenosis and choosing treatment?

And   Reaching a good decisionBased upon the answers you gave to the last question, here are more cho... != How do I ease my fear or anxiety about aortic stenosis and choosing treatment?

Q4.4 **Many patients with aortic stenosis feel anxious. Do you need help managing anxiety?**

- Yes
- Maybe
- No

Display This Question:

If Many patients with aortic stenosis feel anxious. Do you need help managing anxiety? = Yes

Or Many patients with aortic stenosis feel anxious. Do you need help managing anxiety? = Maybe

Q4.5 A member of your medical team can help manage anxiety. There are many ways to manage anxiety that have been shown to work. These include short-term medications, therapy, behavioral modification, exercise, and diet, to name a few.

Q4.6
  **Reaching a good decision**

 Here are the items that you selected as being most important in making a decision about treatment:
  **Items**
 ${Q4.3/AnswerDescription/1} ${Q4.3/ChoiceGroup/SelectedChoicesForAnswer/1}
 ${Q4.3/AnswerDescription/2} ${Q4.3/ChoiceGroup/SelectedChoicesForAnswer/2}
  
You can change your responses by using the Back arrow.

 **Are there other items that would help you make a decision about treatment?**  If so, please write them below. If not, just leave it blank.

________________________________________________________________

Display This Question:

If Reaching a good decisionHere is what you need to know or feel to choose your treatment, based on... [ #2] (Count) = 1

And Reaching a good decisionHere is what you need to know or feel to choose your treatment, based on... [ #3] (Count) = 1

And Reaching a good decisionHere is what you need to know or feel to choose your treatment, based on... [ #4] (Count) = 0

Q4.7
  **Reaching a good decision**
Here are the items that you selected as being most important in reaching a good decision about treatment:
  **Items**
 ${Q4.3/AnswerDescription/1} ${Q4.3/ChoiceGroup/SelectedChoicesForAnswer/1}
 ${Q4.3/AnswerDescription/2} ${Q4.3/ChoiceGroup/SelectedChoicesForAnswer/2}
 ${Q4.3/AnswerDescription/3} ${Q4.3/ChoiceGroup/SelectedChoicesForAnswer/3}
 
You can change your responses by using the Back arrow.

 **Are there other items that would help you make a decision about treatment?**  If so, please write them below. If not, just leave it blank.

________________________________________________________________

Display This Question:

If Reaching a good decisionHere is what you need to know or feel to choose your treatment, based on... [ #4] (Count) = 1

And Reaching a good decisionHere is what you need to know or feel to choose your treatment, based on... [ #5] (Count) = 0

Q4.8
  **Reaching a good decision**

 Here are the items that you selected as being most important in making a decision about treatment:

 ${Q4.3/AnswerDescription/1} ${Q4.3/ChoiceGroup/SelectedChoicesForAnswer/1}
 ${Q4.3/AnswerDescription/2} ${Q4.3/ChoiceGroup/SelectedChoicesForAnswer/2}
 ${Q4.3/AnswerDescription/3} ${Q4.3/ChoiceGroup/SelectedChoicesForAnswer/3}
 ${Q4.3/AnswerDescription/4} ${Q4.3/ChoiceGroup/SelectedChoicesForAnswer/4}
 
You can change your responses by using the Back arrow.

 **Are there other items that would help you make a decision about treatment?**  If so, please write them below. If not, just leave it blank.

________________________________________________________________

Skip To: Q4.9 If Condition: Reaching a good decision He... Is Equal to. Skip To: Reaching a good decision Here are the....

Display This Question:

If Reaching a good decisionHere is what you need to know or feel to choose your treatment, based on... [ #4] (Count) = 1

And Reaching a good decisionHere is what you need to know or feel to choose your treatment, based on... [ #5] (Count) = 1

Q4.9
  **Reaching a good decision**

 Here are the items that you selected as being most important in making a decision about treatment:
  **Specific Items**
 ${Q4.3/AnswerDescription/1} ${Q4.3/ChoiceGroup/SelectedChoicesForAnswer/1}
 ${Q4.3/AnswerDescription/2} ${Q4.3/ChoiceGroup/SelectedChoicesForAnswer/2}
 ${Q4.3/AnswerDescription/3} ${Q4.3/ChoiceGroup/SelectedChoicesForAnswer/3}
 ${Q4.3/AnswerDescription/4} ${Q4.3/ChoiceGroup/SelectedChoicesForAnswer/4}
${Q4.3/AnswerDescription/5} ${Q4.3/ChoiceGroup/SelectedChoicesForAnswer/5}
You can change your responses by using the Back arrow.

 **Are there other items that would help you make a decision about treatment?** If so, please write them below. If not, just leave it blank.

________________________________________________________________

End of Block: Process_Goals

Start of Block: Treatment Features

Q5.1
 
**Part 4. Treatment pros and cons**
  


 Now let's think about the pros and cons of your treatment options.
 
**What features or qualities of treatment are most important to you?**

 Please choose the **3 most important items** from the list **below**.
 
Remember, at times it may seem like we are asking you the same question a different way. This is intentional. Thank you for your patience.   *(The list below came from real patients with aortic stenosis who experienced treatment first-hand.)*

- Long-term outlook (lifespan, heart failure, repeat procedure)
- Impact on my daily living and quality of life
- Impact on my overall health and medications
- Details about the procedures, processes, and complications
- Recovery and post-surgery care (hospital stay, rehab, home care )
- Details about the new valve (materials, durability, benefit to people with chest radiation)
- Other considerations (Covid risk, cost, when can I travel)
- Overall success

| Page Break |  |
| --- | --- |

Q5.2 **Treatment pros and cons**

 Based upon the answers you gave to the last question, here are more choices for you to consider.

 As a reminder, the question is:
 **What features or qualities of treatment are most important to you?**

 Please choose the **3 most important items** from the list **below**.

Display This Choice:

If   Part 4. Treatment pros and cons   Now let's think about the pros and cons of your treatment opt... = Overall success

- Is one procedure more successful in the long term?

Display This Choice:

If   Part 4. Treatment pros and cons   Now let's think about the pros and cons of your treatment opt... = Overall success

- Why would someone choose one procedure over another (SAVR or TAVR)?)

Display This Choice:

If   Part 4. Treatment pros and cons   Now let's think about the pros and cons of your treatment opt... = Long-term outlook (lifespan, heart failure, repeat procedure)

- Will I have a better chance of living longer after replacing my valve?

Display This Choice:

If   Part 4. Treatment pros and cons   Now let's think about the pros and cons of your treatment opt... = Long-term outlook (lifespan, heart failure, repeat procedure)

- If a second procedure is needed, what will it be and when will it happen?

Display This Choice:

If   Part 4. Treatment pros and cons   Now let's think about the pros and cons of your treatment opt... = Long-term outlook (lifespan, heart failure, repeat procedure)

- Knowing if my risk of heart failure increase as I age

Display This Choice:

If   Part 4. Treatment pros and cons   Now let's think about the pros and cons of your treatment opt... = Overall success

- Knowing all the risks involved during and after the procedure (including death)

Display This Choice:

If   Part 4. Treatment pros and cons   Now let's think about the pros and cons of your treatment opt... = Impact on my daily living and quality of life

- How the procedure will affect my quality of life

Display This Choice:

If   Part 4. Treatment pros and cons   Now let's think about the pros and cons of your treatment opt... = Impact on my daily living and quality of life

- Knowing if and when I will be able to return to my normal activities and routines

Display This Choice:

If   Part 4. Treatment pros and cons   Now let's think about the pros and cons of your treatment opt... = Impact on my daily living and quality of life

- The need to change my daily living habits, such as diet and exercise?

Display This Choice:

If   Part 4. Treatment pros and cons   Now let's think about the pros and cons of your treatment opt... = Impact on my daily living and quality of life

- How I'll feel after the procedure, physically and emotionally

Display This Choice:

If   Part 4. Treatment pros and cons   Now let's think about the pros and cons of your treatment opt... = Impact on my overall health and medications

- Impact on my overall state of health, such as clotting, immunity, and COVID-19 risk

Display This Choice:

If   Part 4. Treatment pros and cons   Now let's think about the pros and cons of your treatment opt... = Impact on my overall health and medications

- How being on a blood thinner affects my life

Display This Choice:

If   Part 4. Treatment pros and cons   Now let's think about the pros and cons of your treatment opt... = Impact on my overall health and medications

- If the procedure helps me breathe better

Display This Choice:

If   Part 4. Treatment pros and cons   Now let's think about the pros and cons of your treatment opt... = Impact on my overall health and medications

- The need to change my medications after the procedure

Display This Choice:

If   Part 4. Treatment pros and cons   Now let's think about the pros and cons of your treatment opt... = Details about the procedures, processes, and complications

- Other heart problems that might occur, such as atrial fibrillation (AFib), stroke, or need for a pacemaker

Display This Choice:

If   Part 4. Treatment pros and cons   Now let's think about the pros and cons of your treatment opt... = Details about the procedures, processes, and complications

- The precautions taken to reduce the risk of stroke during the procedure

Display This Choice:

If   Part 4. Treatment pros and cons   Now let's think about the pros and cons of your treatment opt... = Details about the procedures, processes, and complications

- The back-up plan if there are complications during the procedure

Display This Choice:

If   Part 4. Treatment pros and cons   Now let's think about the pros and cons of your treatment opt... = Details about the procedures, processes, and complications

- The invasiveness of the procedure

Display This Choice:

If   Part 4. Treatment pros and cons   Now let's think about the pros and cons of your treatment opt... = Details about the procedures, processes, and complications

- The expected process from admission to discharge

Display This Choice:

If   Part 4. Treatment pros and cons   Now let's think about the pros and cons of your treatment opt... = Details about the procedures, processes, and complications

- Pain or discomfort during or after the procedure

Display This Choice:

If   Part 4. Treatment pros and cons   Now let's think about the pros and cons of your treatment opt... = Details about the procedures, processes, and complications

- The anesthesia used and if I'll be awake

Display This Choice:

If   Part 4. Treatment pros and cons   Now let's think about the pros and cons of your treatment opt... = Details about the procedures, processes, and complications

- Details of the procedure: function, placement, and how it looks

Display This Choice:

If   Part 4. Treatment pros and cons   Now let's think about the pros and cons of your treatment opt... = Recovery and post-surgery care (hospital stay, rehab, home care )

- What recovery will be like

Display This Choice:

If   Part 4. Treatment pros and cons   Now let's think about the pros and cons of your treatment opt... = Recovery and post-surgery care (hospital stay, rehab, home care )

- The need for cardiac rehab after the procedure

Display This Choice:

If   Part 4. Treatment pros and cons   Now let's think about the pros and cons of your treatment opt... = Recovery and post-surgery care (hospital stay, rehab, home care )

- The type of care I will need at home

Display This Choice:

If   Part 4. Treatment pros and cons   Now let's think about the pros and cons of your treatment opt... = Recovery and post-surgery care (hospital stay, rehab, home care )

- The length of stay in the hospital

Display This Choice:

If   Part 4. Treatment pros and cons   Now let's think about the pros and cons of your treatment opt... = Details about the new valve (materials, durability, benefit to people with chest radiation)

- How long the new valve will last

Display This Choice:

If   Part 4. Treatment pros and cons   Now let's think about the pros and cons of your treatment opt... = Details about the new valve (materials, durability, benefit to people with chest radiation)

- What the valves are made of (animal tissue or mechanical)?

Display This Choice:

If   Part 4. Treatment pros and cons   Now let's think about the pros and cons of your treatment opt... = Details about the new valve (materials, durability, benefit to people with chest radiation)

- How many people have had TAVRs to date?

Display This Choice:

If   Part 4. Treatment pros and cons   Now let's think about the pros and cons of your treatment opt... = Details about the new valve (materials, durability, benefit to people with chest radiation)

- How do the benefits and risks of the procedure compare for someone who had chest radiation treatments?

Display This Choice:

If   Part 4. Treatment pros and cons   Now let's think about the pros and cons of your treatment opt... = Other considerations (Covid risk, cost, when can I travel)

- How long before I can resume air travel?

| Page Break |  |
| --- | --- |

Q5.3 **Treatment pros and cons**

 Below are the **features or qualities of treatment** that you just selected**.**
  Please rank these items so that #1 is your most important goal**,** #2 is your next most important, and so on.

|  | Display This Answer:  If If Treatment pros and consBased upon the answers you gave, here Is Greater Than or Equal to 1  #1 | Display This Answer:  If If Treatment pros and consBased q://QID32/SelectedChoicesCount Is Greater Than or Equal to 2  #2 | Display This Answer:  If If Treatment pros and are more choices for Is Greater Than or Equal to 3  #3 |
| --- | --- | --- | --- |
| Display This Choice: |  |  |  |
| Display This Choice: |  |  |  |
| Display This Choice: |  |  |  |

Display This Question:

Q5.4 **Treatment pros and cons**
  
Here are the **features or qualities** that you selected as being most important to you when choosing a treatment:
  **Specific Features** 
${Q5.3/AnswerDescription/1} ${Q5.3/ChoiceGroup/SelectedChoicesForAnswer/1}
 ${Q5.3/AnswerDescription/2} ${Q5.3/ChoiceGroup/SelectedChoicesForAnswer/2}
 ${Q5.3/AnswerDescription/3} ${Q5.3/ChoiceGroup/SelectedChoicesForAnswer/3}
  
If you choose to change your responses, use the Back arrow.
  
If you have any specific concerns that were not mentioned**,** please write them **below**.

________________________________________________________________

Display This Question:

If Treatment pros and cons Below are the features or qualities of treatment that you just selected.... [ #4] (Count) = 1

And Treatment pros and cons Below are the features or qualities of treatment that you just selected.... [ #5] (Count) = 1

Q5.5 **Treatment pros and cons**

 Here are the **features or qualities** that you selected as being most important to you when choosing a treatment:
  **Specific Features**
 ${Q5.3/AnswerDescription/1} ${Q5.3/ChoiceGroup/SelectedChoicesForAnswer/1}
 ${Q5.3/AnswerDescription/2} ${Q5.3/ChoiceGroup/SelectedChoicesForAnswer/2}
 ${Q5.3/AnswerDescription/3} ${Q5.3/ChoiceGroup/SelectedChoicesForAnswer/3}
 ${Q5.3/AnswerDescription/4} ${Q5.3/ChoiceGroup/SelectedChoicesForAnswer/4}
 ${Q5.3/AnswerDescription/5} ${Q5.3/ChoiceGroup/SelectedChoicesForAnswer/5}


 You can change your responses by using the Back arrow.
  
If you have any specific concerns that were not mentioned**,** please write them **below**.

Q5.6
 **Part 5. Concerns or questions**
  


Is there anything else that you would like to discuss with your healthcare provider? Please write it **below**.

________________________________________________________________

________________________________________________________________

________________________________________________________________

________________________________________________________________

________________________________________________________________

End of Block: Treatment Features

Start of Block: Age tailoring

Q6.1
**Part 6. How age affects your treatment choices**
 
 Medical guidelines suggest different treatments based on age and overall health. These suggestions change over time; your preferences matter regardless of your age or overall health.
 
Most people qualify for at least one form of valve treatment. If you are unable to receive SAVR you are likely to qualify for TAVR. The average age of patients who have TAVR is approximately 80 years old. Most patients who have SAVR are under 65 years old.
   You can learn more by answering the question **below**. (To skip this question, click the **NEXT** button.)
 
**How old are you?**

- Less than 65 years old
- 65-80 years old
- Over 80 years old

Display This Question:

If Part 6. How age affects your treatment choices   Medical guidelines suggest different treatments... = Less than 65 years old

Q6.2
 
**How age affects your treatment choices**
  
For people who are **younger than 65 and** have a long life ahead of them (20-plus years), medical guidelines suggest SAVR (surgery) with either a mechanical or tissue valve.*(Refer to the dark blue-colored items below).*

   Each person's situation is different. Age is not the only factor in deciding which choices are available to you. It is important that you discuss options with a heart team, which includes both a cardiac surgeon and an interventional cardiologist (who performs TAVR). Mechanical valves may last longer than tissue valves but require blood thinners for the rest of your life. Blood thinners are needed to prevent blood clots from forming in or around the mechanical valve. Tissue valves do not require **lifelong** blood thinners.

Display This Question:

If Part 6. How age affects your treatment choices   Medical guidelines suggest different treatments... = Over 80 years old

Q6.3

 **How age affects your treatment choices**

 For people who are **over 80** years old or those with a **life expectancy** **of** **less than 10 years**, medical guidelines recommend TAVR (less invasive) over SAVR (surgical). (*Refer to the dark blue-colored items below).*

   Each person's situation is different. Age is not the only factor in deciding which choices are available to you. If SAVR is chosen, a tissue valve is recommended over a mechanical valve because they do not require **lifelong** blood thinners.

Display This Question:

If Part 6. How age affects your treatment choices   Medical guidelines suggest different treatments... = 65-80 years old

Q6.4

 **How age affects your treatment choices**

 For people who are **65-80 years old**, medical guidelines suggest either TAVR (less invasive) or SAVR (surgical) are recommended, depending on what matters to you and your medical condition. 
  If SAVR is chosen, either a mechanical or tissue valve is recommended, based on what matters to you.    Mechanical valves may last longer than tissue valves but require blood thinners for the rest of your life. Blood thinners prevent blood clots from forming in or around the mechanical valve. Tissue valves may not last as long as mechanical valves. They do not require lifelong blood thinners.

End of Block: Age tailoring

Start of Block: decision making questions

Q7.1
 
**Part 7. What treatment are you leaning towards?**

  

  


 Imagine that you were making a treatment choice today.
We have now added a few details to our diagram.

   
 

 The decision about whether TAVR or SAVR is the best fit for you depends on your heart, medical conditions, and other technical factors, in addition to your preferences. Your heart team will provide additional information that may influence your decision.
 
**Which treatment do you presently favor?**

- TAVR (less invasive procedure)
- SAVR (open-heart surgery)
- No valve replacement (medicines/comfort care)
- Not sure at this time

Display This Question:

If   Part 7. What treatment are you leaning towards?     Imagine that you were making a treatment ch... = Not sure at this time

Q7.2
 **What treatment are you leaning towards?**

 Here is some information to help you compare your options. If you would like this information emailed to you, please select that option below.

 **MEDICINES/COMFORT CARE:**
 Because of newer technology, nearly everyone's valve can be fixed.  No one is too old to get their valve replaced. Medicines do not fix your valve. Valve replacement does. People who fix their valve do better than those who don't. On average they: Live longer Have a better quality of life Spend fewer days in the hospital. An advantage of medicines/ comfort care: The start of treatment does not require being in the hospital. Medical therapy may be a good choice for people who do not want to deal with the challenges and risks of a procedure and who understand the risks of not replacing their valve. **SAVR Procedure:**
 SAVR is an open-chest surgery. You can choose a tissue valve or a mechanical valve.

 **TAVR Procedure:**
 The less invasive TAVR procedure always involves a tissue valve. With TAVR you are slightly more likely to need a second procedure to refix the valve than with SAVR. Because TAVR is a newer procedure, there is less long-term data than there is with SAVR. **About Valves** Tissue valves ("bioprosthetic valves") are made of highly manufactured tissue components produced using tissue produced using animals (pig or cow) or human. Mechanical valves may last longer than tissue valves, but you must take blood thinners (anticoagulants) for the rest of your life. People who are on blood thinners have a higher risk of bleeding. **About Blood thinners** Blood thinners are pills that you take on a daily basis to prevent clots from forming in mechanical valves. You will need to have blood work every month. Sometimes injections are needed for short periods of time. Blood thinners interfere with some medications and foods. Blood thinners increase your risk of bleeding. Blood thinners increase the risk of serious injury from falls. If you tend to fall, let your healthcare provider know.

- **Email this information to me**

Display This Question:

If   Part 7. What treatment are you leaning towards?     Imagine that you were making a treatment ch... = No valve replacement (medicines/comfort care)

Q7.3
 
**What treatment are you leaning towards?**

 Because of newer technology, nearly everyone's valve can be fixed.  No one is too old to get their valve replaced Medical treatment does not fix your valve, valve replacement does.
 People who fix their valve do better than those who don't.  On average, they: Live longer Have a better quality of life Spend fewer days in the hospital
  An advantage of medical treatment: The start of treatment does not require being in the hospital. Medical therapy may be a good choice for people who do not want to deal with the challenges and risks of a procedure.

 **Are you sure that you prefer medical therapy over valve replacement?**

- Yes, I'm sure
- I'm not sure

Display This Question:

If   Part 7. What treatment are you leaning towards?     Imagine that you were making a treatment ch... = TAVR (less invasive procedure)

Q7.4
 
**What treatment are you leaning towards?**

 When you have TAVR (less invasive), you can only receive a tissue valve.
 
Tissue valves or "bioprosthetic valves" are made from animal (cow or pig) or human tissue. With TAVR you are slightly more likely to need a second procedure to refix the valve than with SAVR. Because TAVR is a newer procedure, there is less long-term data.

Display This Question:

If   Part 7. What treatment are you leaning towards?     Imagine that you were making a treatment ch... = SAVR (open-heart surgery)

| 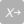 |
| --- |

Q7.5

 **What treatment are you leaning towards?**

 SAVR (Surgical Aortic Valve Replacement) is an open-chest surgery. SAVR can be done with a tissue valve or a mechanical valve. Tissue valves or "bioprosthetic valves" are made from animal (pig or cow) or human tissue. Mechanical valves may last longer than tissue valves, but you must take blood thinners (anticoagulants) for the rest of your life. People who are on blood thinners have a higher risk of bleeding.
 
**What kind of valve are you most interested in receiving?**

- Mechanical valve
- Tissue valve
- Not sure

Display This Question:

If   Part 7. What treatment are you leaning towards?     Imagine that you were making a treatment ch... = TAVR (less invasive procedure)

Or   Part 7. What treatment are you leaning towards?     Imagine that you were making a treatment ch... = SAVR (open-heart surgery)

Or   Part 7. What treatment are you leaning towards?     Imagine that you were making a treatment ch... = No valve replacement (medicines/comfort care)

Q7.6 **What treatment are you leaning towards?**

 **Briefly describe why you favor** ${Q7.1/ChoiceGroup/SelectedChoices}.

________________________________________________________________

Q7.7
  Are there any extenuating (special) circumstances that are influencing your decision? If not, please click **NEXT**.

________________________________________________________________

End of Block: decision making questions

Start of Block: Anticoagulation question

Q8.1
**Blood thinners**
   Blood thinners are pills that you take on a daily basis to prevent clots from forming in mechanical valves. You will need to have blood work every month. Sometimes injections are needed for short periods of time. Blood thinners interfere with some medications and foods. Blood thinners increase your risk of bleeding. Blood thinners increase the risk of serious injury from falls. If you tend to fall, let your healthcare provider know.  
Are you willing to take blood thinners (Coumadin or Warfarin pills) for the rest of your life?

- Yes
- Not sure
- No

End of Block: Anticoagulation question

Q9.1
 **Summary** **of your Goals and Preferences**

 To find the treatment that's best for you, share this summary of your goals and preferences with your heart team.                                      
 **SPEAK UP:** tell your provider that you want to **${e://Field/RoleP}**
  
**TELL your provider what you need to know or feel to choose treatment:**
${e://Field/1ProcessP} ${Q4.3/ChoiceGroup/SelectedChoicesForAnswer/1}
 ${e://Field/2ProcessP} ${Q4.3/ChoiceGroup/SelectedChoicesForAnswer/2}
 ${e://Field/3ProcessP} ${Q4.3/ChoiceGroup/SelectedChoicesForAnswer/3}
 ${e://Field/OtherProcessP} ${Q4.6/ChoiceTextEntryValue}${Q4.7/ChoiceTextEntryValue}${Q4.8/ChoiceTextEntryValue}
 
**SHARE your treatment goals with your provider:**
 ${e://Field/1GoalP} ${Q3.5/ChoiceGroup/SelectedChoicesForAnswer/1}
${e://Field/2GoalP} ${Q3.5/ChoiceGroup/SelectedChoicesForAnswer/2}
${e://Field/3GoalP} ${Q3.5/ChoiceGroup/SelectedChoicesForAnswer/3}
${e://Field/OtherGoalP} ${q://QID202/ChoiceTextEntryValue}${Q3.8/ChoiceTextEntryValue}
 
**DISCUSS these topics about treatment:**
${e://Field/1FeatureP} ${Q5.3/ChoiceGroup/SelectedChoicesForAnswer/1}
 ${e://Field/2FeatureP} ${Q5.3/ChoiceGroup/SelectedChoicesForAnswer/2}
 ${e://Field/3FeatureP} ${Q5.3/ChoiceGroup/SelectedChoicesForAnswer/3}
${e://Field/OtherFeatureP} ${Q5.4/ChoiceTextEntryValue}${Q5.5/ChoiceTextEntryValue}

 **${e://Field/question}** ${Q5.6/ChoiceTextEntryValue} ${e://Field/HCP}
  
**Your favored treatment at this time**: ${Q7.1/ChoiceGroup/SelectedChoices}${e://Field/PuncSAVR} ${e://Field/NoAVR}${Q7.5/ChoiceGroup/SelectedChoices}${e://Field/Coag} ${e://Field/because} ${Q7.6/ChoiceTextEntryValue}
 **${e://Field/Special}** ${e://Field/Special1}

  ${date://CurrentDate/SL}      
 
Please click Next to continue.

Q9.2
  **How to make the most of your summary**
 
Discussing your summary with your healthcare provider at your next appointment will help you get your voice heard when choosing treatment.
 
Your summary will be emailed to you and your healthcare provider. You can also print it yourself.


 If would like us to print it for you and mail it to you, please click the box below. If you select this, you will be asked for your mailing address.

- Print it and mail it to me.

Display This Question:

If   How to make the most of your summary   Discussing your summary with your healthcare provider at... = Print it and mail it to me.

Q9.3 Please provide your contact information below so we can mail you a paper copy of your summary.

- **Name** ________________________________________________
- **Address** ________________________________________________
- **Address (2)** ________________________________________________
- **City** ________________________________________________
- **State** ________________________________________________
- **Zip code** ________________________________________________

|  |  |
| --- | --- |

9.4
Which cardiology doctor or healthcare provider will you be seeing at this appointment? If your clinician is not listed (or if no clinicians are listed), please select "Other".

Display This Choice:

If QState_Q183 = AL

Or QState_Q183 = TN

Or QState_Q183 = GA

Or QState_Q183 = NC

- Megan Coylewright, MD, MPH

Display This Choice:

If QState_Q183 = TN

Or QState_Q183 = AL

Or QState_Q183 = GA

Or QState_Q183 = NC

- Larry Shears, MD

Display This Choice:

If QState_Q183 = TN

Or QState_Q183 = AL

Or QState_Q183 = GA

Or QState_Q183 = NC

- Jeffrey Poyntner, MD

Display This Choice:

If QState_Q183 = TN

Or QState_Q183 = AL

Or QState_Q183 = GA

Or QState_Q183 = NC

- John Golding, MD

Display This Choice:

If QState_Q183 = TN

Or QState_Q183 = AL

Or QState_Q183 = GA

Or QState_Q183 = NC

- Courtney Ayres, PA

Display This Choice:

If QState_Q183 = TX

Or QState_Q183 = UT

- Aaron Horne, MD

Display This Choice:

If QState_Q183 = TN

Or QState_Q183 = KY

Or QState_Q183 = AL

Or QState_Q183 = GA

- Brian Lindman, MD, MPH

Display This Choice:

If QState_Q183 = TN

Or QState_Q183 = KY

Or QState_Q183 = AL

Or QState_Q183 = GA

- Melissa Levack, MD

Display This Choice:

If QState_Q183 = TN

Or QState_Q183 = KY

Or QState_Q183 = AL

- William G. McMaster, MD

Display This Choice:

If QState_Q183 = TN

Or QState_Q183 = KY

Or QState_Q183 = AL

- Ashish Shah, MD

Display This Choice:

If QState_Q183 = TN

Or QState_Q183 = KY

Or QState_Q183 = AL

- Kashish Goel, MD

Display This Choice:

If QState_Q183 = TN

Or QState_Q183 = KY

Or QState_Q183 = AL

- Jared M. O'Leary, MD

Display This Choice:

If QState_Q183 = VT

Or QState_Q183 = NH

Or QState_Q183 = NY

Or QState_Q183 = ME

- Melissa Beaudry, NP

Display This Choice:

If QState_Q183 = VT

Or QState_Q183 = NH

Or QState_Q183 = NY

- Stefan Lischke, MD

Display This Choice:

If QState_Q183 = VT

Or QState_Q183 = NH

Or QState_Q183 = NY

- Joachim Mueller, MD

Display This Choice:

If QState_Q183 = VT

Or QState_Q183 = NH

Or QState_Q183 = NY

- Andrea Carey, NP

Display This Choice:

If QState_Q183 = CA

Or QState_Q183 = NV

Or QState_Q183 = WA

- Tom Nguyen, MD

Display This Choice:

If QState_Q183 = CA

Or QState_Q183 = NV

Or QState_Q183 = WA

- Harsh Agrawal, MD

Display This Choice:

If QState_Q183 = CA

Or QState_Q183 = AZ

Or QState_Q183 = NV

- Bina Ahmed, MD
- Other

Display This Question:

If Which cardiology doctor or healthcare provider will you be seeing at this appointment? If your cl... = Other

Q9.5 Which healthcare provider will you be seeing at this appointment? You may not have all the information, but do the best you can.

- Provider **Last Name** ________________________________________________
- Provider First Name ________________________________________________
- City ________________________________________________
- State ________________________________________________
- Phone # ________________________________________________

| Page Break |
| --- |

## Appendix L: Pilot Study Evaluation Surveys (Phase 5)

### Appendix L1: Pilot Study T0 Intake Survey

T0 - AS Pilot Intake

Start of Block: Introduction and Eligibility screener

| 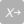 |
| --- |

T0_AS

 **Welcome to the AVITA Study**
 
Thank you for your interest in our study.
 
This study hopes to help patients with aortic stenosis make treatment decisions and help healthcare providers understand what's important to their patients. *Aortic Stenosis* is a narrowed heart valve that doesn't open properly, resulting in impaired heart function.

 Please answer a few questions to see if you qualify for the study and to help us understand our participants.
  **Have you been told by a health care provider that you have Aortic Stenosis?**

- Yes
- No

| Page Break |  |
| --- | --- |

Display This Question:

If Welcome to the AVITA Study    Thank you for your interest in our study.   This study hopes to hel... = No

| 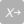 |
| --- |

T0_CareG Are you a caregiver for someone who has Aortic Stenosis?

- Yes
- No

Display This Question:

If Are you a caregiver for someone who has Aortic Stenosis? = Yes

Q1.3 For the questions that follow, please complete them on behalf of the person who has aortic stenosis.

| 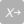 |
| --- |

T0_ASOpt Are you considering aortic valve replacement or another treatment for your aortic stenosis?

- Yes
- No

Skip To: Q1.10 If Are you considering aortic valve replacement or another treatment for your aortic stenosis? = No

| Page Break |  |
| --- | --- |

Display This Question:

If Are you a caregiver for someone who has Aortic Stenosis? = No

Q1.5 Thank you for your interest in this study. You are not eligible to participate in this study because you are not a patient or caregiver for someone with aortic stenosis.


If you have any questions, please contact us at info@valvesurvey.com.

Skip To: End of Survey If Thank you for your interest in this study. You are not eligible to participate in this study beca... Is Displayed

| 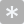 |
| --- |

T0_Age
How old are you?

________________________________________________________________

| 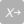 |
| --- |

T0_ApptYN Do you have an upcoming appointment with a cardiology clinician (a cardiologist, cardiac surgeon, nurse practitioner or physician assistant) to discuss treatment for your aortic stenosis within the next month (before ${date://OtherDate/FL/+35%20day})?

- Yes, my appointment is scheduled before ${date://OtherDate/FL/+35%20day}
- No, my appointment is scheduled for ${date://OtherDate/FL/+36%20day} or later
- No, I do not have an appointment scheduled.

Display This Question:

If Do you have an upcoming appointment with a cardiology clinician (a cardiologist, cardiac surgeon,... = No, my appointment is scheduled for ${date://OtherDate/FL/+36%20day} or later

Or Do you have an upcoming appointment with a cardiology clinician (a cardiologist, cardiac surgeon,... = No, I do not have an appointment scheduled.

| 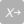 |
| --- |

Q1.7 You are not eligible because you do not have an appointment scheduled with your healthcare provider within the next month.


We are continually recruiting for this study. You may be eligible at a later date. If you have any questions, please contact us at info@valvesurvey.com.


If there are future openings in the study, may we contact you at a later date to be in the study?

- Yes
- No

Skip To: End of Survey If You are not eligible because you do not have an appointment scheduled with your healthcare provid... , Yes Is Displayed

T0_APPT
Please enter the date for your upcoming cardiology appointment. Please include a leading zero for a single digit month or day.


 For example, January 3, 2021 would be written as:
Month (MM) = 01
Day (DD) = 03
Year (YYYY) = 2022

- **Month** (MM) ________________________________________________
- **Day** (DD) ________________________________________________
- **Year** (YYYY) ________________________________________________

Display This Question:

If If How old are you? Text Response Is Less Than 18

Q1.10 I'm sorry but you are not eligible to participate in this study.  Thank you for your interest.


If you have any questions, please contact us at info@valvesurvey.com.

Skip To: End of Survey If I'm sorry but you are not eligible to participate in this study.  Thank you for your interest. If... Is Displayed

End of Block: Introduction and Eligibility screener

Start of Block: Intake Baseline: AS patients

Display This Question:

If Contact List FirstName Is Empty

Or Contact List LastName Is Empty

Or Contact List Email1 Is Empty

T0_contact
What is your contact information? 


We keep all of your information and personal identifiers encrypted and strictly confidential.

- First name ________________________________________________
- Last name ________________________________________________
- Email ________________________________________________

| Page Break |  |
| --- | --- |

| 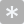 |
| --- |

Q168 What is the Zip Code for your primary residence (where you receive care for your aortic stenosis)?

________________________________________________________________

End of Block: Intake Baseline: AS patients

Start of Block: Appt Info

T0_HCPName
Which clinician will you be seeing at your upcoming cardiology appointment? If your clinician is not listed (or if no clinicians are listed), please select "Other".
Display This Choice:

If QState_Q168 = AL

Or QState_Q168 = TN

Or QState_Q168 = GA

Or QState_Q168 = NC

- Megan Coylewright, MD, MPH

Display This Choice:

If QState_Q168 = TN

Or QState_Q168 = AL

Or QState_Q168 = GA

Or QState_Q168 = NC

- Larry Shears, MD

Display This Choice:

If QState_Q168 = TN

Or QState_Q168 = AL

Or QState_Q168 = GA

Or QState_Q168 = NC

- Jeffrey Poyntner, MD

Display This Choice:

If QState_Q168 = TN

Or QState_Q168 = AL

Or QState_Q168 = GA

Or QState_Q168 = NC

- John Golding, MD

Display This Choice:

If QState_Q168 = TN

Or QState_Q168 = AL

Or QState_Q168 = GA

Or QState_Q168 = NC

- Courtney Ayres, PA

Display This Choice:

If QState_Q168 = TX

Or QState_Q168 = UT

- Aaron Horne, MD

Display This Choice:

If QState_Q168 = TN

Or QState_Q168 = KY

Or QState_Q168 = AL

- Brian Lindman, MD, MPH

Display This Choice:

If QState_Q168 = TN

Or QState_Q168 = KY

Or QState_Q168 = AL

- Melissa Levack, MD

Display This Choice:

If QState_Q168 = TN

Or QState_Q168 = KY

Or QState_Q168 = AL

- William G. McMaster, MD

Display This Choice:

If QState_Q168 = TN

Or QState_Q168 = KY

Or QState_Q168 = AL

- Ashish Shah, MD

Display This Choice:

If QState_Q168 = TN

Or QState_Q168 = KY

Or QState_Q168 = AL

- Kashish Goel, MD

Display This Choice:

If QState_Q168 = TN

Or QState_Q168 = KY

Or QState_Q168 = AL

- Jared M. O'Leary, MD

Display This Choice:

If QState_Q168 = VT

Or QState_Q168 = NH

Or QState_Q168 = NY

- Melissa Beaudry, NP

Display This Choice:

If QState_Q168 = VT

Or QState_Q168 = NH

Or QState_Q168 = NY

- Stefan Lischke, MD

Display This Choice:

If QState_Q168 = VT

Or QState_Q168 = NH

Or QState_Q168 = NY

- Joachim Mueller, MD

Display This Choice:

If QState_Q168 = VT

Or QState_Q168 = NH

Or QState_Q168 = NY

- Andrea Carey, NP

Display This Choice:

If QState_Q168 = CA

Or QState_Q168 = NV

Or QState_Q168 = WA

- Tom Nguyen, MD

Display This Choice:

If QState_Q168 = CA

Or QState_Q168 = NV

Or QState_Q168 = WA

- Harsh Agrawal, MD

Display This Choice:

If QState_Q168 = CA

Or QState_Q168 = AZ

Or QState_Q168 = NV

- Bina Ahmed, MD
- **Other**

Display This Question:

If Which clinician will you be seeing at your upcoming cardiology appointment? If your clinician is... = <strong>Other</strong>

T0_HCPOtherName Which clinician will you be seeing at your upcoming cardiology appointment? You may not have all the information, but do the best you can.

- Provider **Last Name** ________________________________________________
- Provider First Name ________________________________________________
- City ________________________________________________
- State ________________________________________________
- Phone # ________________________________________________

End of Block: Appt Info

Start of Block: SocioDemographics

| 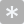 |
| --- |

Q2.1  What **year** did you learn that you had aortic stenosis? If you are not sure, please give your best guess.

________________________________________________________________

| Page Break |  |
| --- | --- |

Display This Question:

If Welcome to the AVITA Study    Thank you for your interest in our study.   This study hopes to hel... = Yes

| 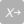 |
| --- |

Q2.2   Have you ever had a **valve replacement** for your aortic stenosis?

- Yes, **TAVR** (**Transcatheter** aortic valve replacement)
- Yes, **SAVR** (**Surgical** aortic valve replacement), or surgical aortic valve implantation (TAVI)
- No

| 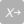 |
| --- |

T0_Sex What is your gender?

- Male
- Female
- Choose not to respond

| Page Break |  |
| --- | --- |

| 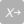 |
| --- |

T0_race Which racial group or groups best describes you? Please select all that apply.

- Hispanic or Latino
- Black or African American
- White
- Asian
- Native American or Alaska Native
- Native Hawaiian or other Pacific Islander
- Other (please specify) ________________________________________________
- ⊗Choose not to respond

| Page Break |  |
| --- | --- |

| 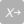 | 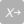 |
| --- | --- |

T0_Ins What type of health insurance do you presently have? Please select all that apply.

- Medicaid or other type of state medical assistance
- Medicare
- Private health insurance offered through an employer or a union
- Private health insurance paid by the individual, such as The Patient Protection and Affordable Care Act (ACA, or "Obamacare")
- Military or Veterans (VA)
- Uninsured
- Other ________________________________________________

| 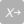 |
| --- |

T0_Ed What is the highest grade in school you completed?

- Less than high school
- High school graduate or GED
- Some college
- Two year college or technical school
- College graduate
- Graduate school or professional degree

| Page Break |  |
| --- | --- |

| 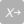 |
| --- |

T0_Eng. Is English your first language?

- Yes
- No

| 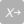 |
| --- |

T0_Lit How often do you have someone help you when you read hospital materials?

- Never
- Rarely
- Sometimes
- Often
- Always

| 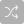 | 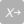 | 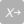 |
| --- | --- | --- |

PHQ2 Over the past 2 weeks, how often have you been bothered by any of the following problems?

|  | Not at all | Several Days | More Than Half the Days | Nearly Every Day |
| --- | --- | --- | --- | --- |
| Little interest or pleasure in doing things |  |  |  |  |
| Feeling down, depressed, or hopeless |  |  |  |  |

End of Block: SocioDemographics

Start of Block: ICF and options

| 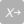 |
| --- |

Thank you! You are eligible to participate. Please review the document below and click “yes” to agree to participate.

**Consent Form for Participation in a Research Study**

**Study Title**: The AVITA Study

**DETAILED RESEARCH CONSENT**

You are being asked for your consent to take part in a research study. The following section provides a summary of this study; it describes the key information that we believe most people need to decide whether to take part in this project.

**What should I know about this research?**

- Participants are limited to about 25 adults with aortic stenosis who are considering valve replacement and have an upcoming appointment to discuss treatment options for aortic stenosis.
- Participants will be asked to view an online learning tool about aortic stenosis (called AVITA) and to complete 2 online surveys.
- Taking part in this research is voluntary. There are no consequences if you choose not to participate or decide to participate now and later drop out.
- The website, Valvesurvey.com, describes the study in more detail.
- You are invited to ask all your questions before you decide whether you want to participate. Contact information is listed below.

**Why is this research being done?**

- Choosing treatment for aortic stenosis can be difficult. We are testing an online learning tool called AVITA (**A**ortic **V**alve **i**n **T**reatment **A**pproaches) that was designed to help people explore their treatment options for aortic stenosis.

**How long will I be in this research?**

You will be enrolled in this study until you complete the final survey, which will be emailed to you shortly after your upcoming heart team appointment.

**What happens to me if I agree to take part in this research?**

- You will be asked to complete 2 online surveys and to view an online learning tool.
- The first survey follows this consent form and should take about 5-10 minutes.
- Then you will be connected (automatically) to the AVITA learning tool, which takes about 15-20 minutes to review.
- AVITA asks about your treatment goals and preferences. It gives you personalized feedback as well as a printable summary of your goals and preferences that you can share with your heart team.
- AVITA will email your summary to you and securely email or fax it to your designated heart team clinician(s). You can also print it and bring it with you to the appointment. The study team can also print and mail it to you.
- Shortly after your heart team appointment, you will be emailed another survey asking about your treatment decision. That survey should take 10-15 minutes to complete.
- Information regarding your final treatment choice will be shared with the study team.
- All study surveys are online. You can contact the research team for help with any surveys.

**What are my responsibilities if I take part in this research?**

If you take part in this research, you will be asked to review the AVITA online learning tool, to consider discussing the summary report with your designated heart team clinician, and to complete 2 online surveys.

**Could being in this research hurt me?**

You may feel emotionally uneasy when asked to think about treatment options for aortic stenosis. To the best of our ability, all the information you give us will remain completely confidential but there is a risk of loss of confidentiality through data entered online. We take many steps to minimize the risk of losing confidentiality by encrypting data, using secure software, separating personal identifiers from stored data, and restricting access to the data only to the study team.

**Will it cost me money to take part in this research?**

We do not expect that you will incur any expenses while completing these online surveys. We do, however, offer a small compensation for your effort (see below).

**Will I be paid for taking part in this research?**

You will be paid for your time and effort with online Amazon gift cards or equivalent (total of $75 for all study activities). The first payment of $40 will be sent after completing this survey and the AVITA tool. The next (final) payment of $35 will be sent after completing the survey after your heart team appointment.

**Will being in this research benefit me?**

There are no direct benefits to participants other than possibly learning more about your treatment options. However, your participation may help us improve decision-making tools about treating aortic stenosis.

**What other choices do I have besides taking part in this research?**

Your alternative is to not take part in the research.

**What happens to the information collected for this research?**

Records of your participation in this study will be held confidential so far as permitted by law. However, the study principal investigator and, under certain circumstances, WCG Independent Review Board (the name of the oversight organization) will be able to inspect and have access to confidential data that identifies you by name. Any publication or presentation of the data will not identify you.

**What if I am injured because of taking part in this research?**

We do not expect that you will be hurt by taking part in this research study. No funds have been set aside for payments or other forms of compensation (such as for lost wages, lost time, or discomfort) other than compensation for your participation (see above). However, you do not give up your legal rights by signing this consent form.

**Can I be removed from this research without my approval?**

We will tell you about any new information that may affect your health, welfare, or choice to stay in this research. We may end your participation in this study for any of the following reasons:

- If the study is cancelled by the sponsor or the WCG Independent Review Board (IRB); or
- For administrative reasons.

**Who can answer my questions about this research?**

Please direct any questions to the research team at info@valvesurvey.com or to Dr. Nananda Col at (207) 272-9829.    
If you have any concerns about your rights as a study participant or the way that you have been treated, talk to the research team at the phone number listed above. This research is being overseen by WCG IRB. An IRB is a group of people who perform independent review of research studies. You may talk to them at 855-818-2289 or researchquestions@wcgirb.com if:

- You have questions, concerns, or complaints that are not being answered by the research team.
- You cannot reach the research team.
- You want to talk to someone else about the research.
- You have questions about your rights as a research participant.

**What happens if I agree to be in this research, but change my mind later?**

If you decide to leave this research study, please contact the research team (info@valvesurvey.com) so that we can remove you from the study contact list and provide follow up information if needed.

**Statement of Consent:**

I have read the above consent form and agree to participate in this study, and I am over 18 years of age.

- Yes
- No

| Page Break |  |
| --- | --- |

Display This Question:

If Thank you! You are eligible to participate. Please review the document below. RESEARCH PARTICIPAN... = No

Q5.3 To participate in this study you must give informed consent. You can go back to change your response. If not, thank you for your interest.


If you have any questions, please contact us at info@valvesurvey.com.

Skip To: End of Survey If To  participate in this study you must give informed consent. You can go back to change your resp... Is Displayed

End of Block: ICF and options

Start of Block: Transition

Q6.1 Thank you for agreeing to help us with our study!
After you complete a few more questions, you can view the AVITA tool.

End of Block: Transition

Start of Block: Tx Pref, DCS, Self-Efficacy

| 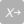 |
| --- |

T0_DRole When making a decision about treating your aortic stenosis, which ONE of the following best describes how you would like to make this decision?

- Make the final selection myself about which treatment I will receive.
- Make the final selection myself after seriously considering my cardiology clinician's opinion.
- Share responsibility with my cardiology clinician when deciding what treatment is best for me.
- Have my cardiology clinician make the final decision after considering my opinion.
- Leave all decisions regarding treatment to my cardiology clinician.

| Page Break |  |
| --- | --- |

| 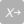 | 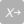 |
| --- | --- |

T0_DStage In choosing treatment for your aortic stenosis, would you say that you...

- Have not begun to think about the choices.
- Have not begun to think about the choices, but are interested in doing so.
- Are considering the options now.
- Are close to selecting an option.
- Have already made a decision, but are still willing to reconsider.
- Have already made a decision and are unlikely to change your mind.

| 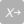 |
| --- |

T0_TxPref At this time, which treatment do you prefer for your aortic stenosis? If you don't have a preference now, select 'unsure'.

- TAVR (Transcatheter aortic valve replacement)
- SAVR (Surgical aortic valve replacement)
- No valve replacement (medications and/or comfort care)
- Unsure

| Page Break |  |
| --- | --- |

| 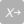 | 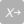 |
| --- | --- |

T0_DQual Thinking about your treatment plan for aortic stenosis, please indicate how strongly you agree or disagree with the statements below.  If you do not have a plan, please select *not applicable*.

|  | Not applicable/no plan | Strongly Disagree | Somewhat Disagree | Neither agree nor disagree | Somewhat Agree | Strongly Agree |
| --- | --- | --- | --- | --- | --- | --- |
| My treatment plan will help me achieve my treatment goals. |  |  |  |  |  |  |
| My treatment plan reflects what’s important to me when I think about the pros and cons of treatment. |  |  |  |  |  |  |

| Page Break |  |
| --- | --- |

| 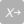 |
| --- |

T0_SURE In thinking about your treatment options for aortic stenosis, please answer the following questions:

|  | Yes | No |
| --- | --- | --- |
| Do you know the benefits and risks of each option? |  |  |
| Are you clear about which benefits and risks matter most to you? |  |  |
| Do you have enough support and advice to make a choice? |  |  |
| Do you feel SURE about the best choice for you? |  |  |

T0_DSelfEff Thinking about **your aortic stenosis**, how **confident** do you feel that you can:

|  | Very Confident | Somewhat Confident | Not Confident |
| --- | --- | --- | --- |
| Get the facts about the benefits of each choice. |  |  |  |
| Get the facts about the risks and side effects of each choice. |  |  |  |
| Get the facts about the medication choices available to me |  |  |  |
| Understand the information enough to be able to make a choice |  |  |  |
| Ask questions without feeling embarrassed |  |  |  |
| Express your concerns about each choice |  |  |  |
| Ask for advice |  |  |  |
| Figure out the treatment choice that best suits me. |  |  |  |
| Handle unwanted pressure from others in making my choice |  |  |  |
| Let the clinic team know what's best for me |  |  |  |
| Delay my decision if I feel I need more time |  |  |  |

| Page Break |  |
| --- | --- |

End of Block: Tx Pref, DCS, Self-Efficacy

Start of Block: Patient-Provider Communication

| 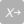 |
| --- |

T0_GoalTalk
As of today, have you discussed your goals for treating your aortic stenosis with your cardiologist or cardiac surgeon? Goals refers to the things you hope to accomplish or avoid by treating your aortic stenosis.

- Yes
- No

End of Block: Patient-Provider Communication

Start of Block: Knowledge (Subjective, Objective)

| 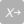 |
| --- |

T0_KnowSR At this time, how would you rate your knowledge of the following?

|  | Extremely knowledgeable | | Very knowledgeable | | Moderately knowledgeable | | Slightly knowledgeable | | Not knowledgeable at all | |
| --- | --- | --- | --- | --- | --- | --- | --- | --- | --- | --- |
| Your aortic stenosis |  |  | |  | |  | |  | |  |
| Your options for treating aortic stenosis |  |  | |  | |  | |  | |  |

| Page Break |  |
| --- | --- |

| 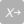 | 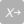 |
| --- | --- |

T0_Know
The statements below are about treating aortic stenosis. Some are true and some are false. We don't expect you to know all the answers. Please give your best guess when answering them.

|  | True | False |
| --- | --- | --- |
| One choice is to take medicines and not have a valve replacement. |  |  |
| I am more likely to feel better with a valve replacement compared to medicines alone. |  |  |
| Medicines alone (without valve replacement) will help me live longer. |  |  |
| People who get a mechanical valve need to take blood thinners for the rest of their life. |  |  |
| Mechanical valves are just as likely to need replacement as tissue valves. |  |  |

End of Block: Knowledge (Subjective, Objective)

Start of Block: Typology

| 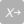 | 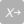 |
| --- | --- |

Typ9. How much do you agree or disagree with the following general statements about your health (including your aortic stenosis):

|  | 1 (Strongly Disagree) | 2 | 3 | 4 (Neutral) | 5 | 6 | 7 (Strongly Agree) |
| --- | --- | --- | --- | --- | --- | --- | --- |
| My health conditions can be easily managed by making lifestyle changes (e.g., diet and exercise). |  |  |  |  |  |  |  |
| I prefer alternative approaches to standard medical practices. |  |  |  |  |  |  |  |
| I pay special attention to advertisements related to my health conditions. |  |  |  |  |  |  |  |
| I am extremely comfortable using websites that consistently offer useful information about my health conditions. |  |  |  |  |  |  |  |
| I don't want to burden my family and friends by discussing my health issues with them. |  |  |  |  |  |  |  |
| My symptoms make it difficult to complete day-to-day tasks. |  |  |  |  |  |  |  |
| I fear that these symptoms will negatively affect my body and its functioning in the long run. |  |  |  |  |  |  |  |
| I do my own research on any treatment my cardiologist recommends. |  |  |  |  |  |  |  |
| I would not trust another doctor to provide me with information related to new treatment options. |  |  |  |  |  |  |  |

| 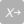 |
| --- |

Typo1 I wait and hope the symptoms go away when I experience any new symptoms/health issues.

- No
- Yes

End of Block: Typology

Start of Block: Follow up and Feedback

T0_Comments
If you have any thoughts about the survey, please write them below.
 
**Please click the next (>>)** to exit this survey and be automatically sent to the AVITA questionnaire. When you exit this survey, you will also be emailed the link to AVITA if you prefer to complete AVITA at a later time.

________________________________________________________________

________________________________________________________________

________________________________________________________________

________________________________________________________________

________________________________________________________________

End of Block: Follow up and Feedback

### Appendix L2: Pilot Study T1 Post-AVITA Evaluation Survey

Start of Block: AS PREF Evaluation QUESTIONS

Q10.2
We would like to know about your experience using the **AVITA** tool.  The summary report and gift certificate will be emailed after completing the questions below.


  **To what extent were your personal goals and values included in the questions asked in the survey?**

- All were included
- Nearly all were included
- Most were included
- Some were included
- Very few were included

| Page Break |  |
| --- | --- |

Q10.3
  **How strongly do you agree or disagree with each of the following statements about AVITA?**

|  | **Strongly agree** | Somewhat agree | Neither agree nor disagree | Somewhat disagree | **Strongly disagree** |
| --- | --- | --- | --- | --- | --- |
| It was **easy to use**. |  |  |  |  |  |
| I would **recommend it** to others with aortic stenosis. |  |  |  |  |  |
| I **trusted** the information provided. |  |  |  |  |  |
| It contained the **right amount of information**. |  |  |  |  |  |
| The personal summary **reflects what matters to me**. |  |  |  |  |  |
| AVITA helped me **identify my own goals and priorities** regarding valve replacement. |  |  |  |  |  |
| AVITA will help me **talk to my cardiologist** about what matters most to me. |  |  |  |  |  |
| AVITA made me **want to be more involved in decisions** about treating my valve. |  |  |  |  |  |
| AVITA helped me understand **there are choices** for treating my aortic stenosis |  |  |  |  |  |
| AVITA helped me understand the **reasons I might want to have TAVR**. |  |  |  |  |  |
| AVITA helped me understand the reasons I might **not want to have TAVR**. |  |  |  |  |  |

Q272  **Thinking about how you now manage your aortic stenosis, did the AVITA Questionnaire change how confident you feel that you can:**

|  | Yes, A Lot | Yes, A Little | No |
| --- | --- | --- | --- |
| Get the facts about the medication choices available to you. |  |  |  |
| Get the facts about the benefits of each choice. |  |  |  |
| Get the facts about the risks and side effects of each choice. |  |  |  |
| Understand the information enough to be able to make a choice |  |  |  |
| Ask questions without feeling embarrassed) |  |  |  |
| Express your concerns about each choice (T2_DSE6) |  |  |  |
| Ask for advice |  |  |  |
| Figure out the treatment choice that best suits you. |  |  |  |
| Handle unwanted pressure from others in making your choice |  |  |  |
| Let the clinic team know what's best for you |  |  |  |
| Delay your decision if you feel you need more time |  |  |  |

End of Block: Self-Efficacy

Start of Block: Knowledge Subjective, Objective

| 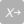 |
| --- |

Q11.1 After reviewing the AVITA tool, how would you rate your knowledge of the following?

|  | Extremely knowledgeable | Very knowledgeable | Moderately knowledgeable | Slightly knowledgeable | Not knowledgeable at all |
| --- | --- | --- | --- | --- | --- |
| Your aortic stenosis |  |  |  |  |  |
| Your options for treating aortic stenosis |  |  |  |  |  |

| Page Break |  |
| --- | --- |

| 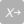 |
| --- |

Q11.2
The statements below are about treating aortic stenosis. Some are true and some are false. We don't expect you to know all the answers. Please give your best guess when answering them.

|  | True | False |
| --- | --- | --- |
| One choice is to take medicines and not have a valve replacement. |  |  |
| I am more likely to feel better with a valve replacement compared to medicines alone. |  |  |
| Medicines alone (without valve replacement) will help me live longer. |  |  |
| People who get a mechanical valve need to take blood thinners for the rest of their life. |  |  |
| Mechanical valves are just as likely to need replacement as tissue valves. |  |  |
| There is an age limit for getting TAVR |  |  |

Q11.3
  If you have any suggestions about the AVITA tool, please write them in the box **below**.

________________________________________________________________

________________________________________________________________

________________________________________________________________

________________________________________________________________

________________________________________________________________

End of Block: Knowledge Subjective, Objective

### Appendix L3: Pilot Study T2 Patient Survey

T2_Email

 **Welcome back to the AVITA study.**

Please enter your email and Zip code below so we can ensure that it is you.
 
**Email:**

________________________________________________________________

Display This Question:

If Zip_AS Is Empty

| 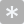 |
| --- |

Q1.5 **Zip code:**

________________________________________________________________

| 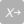 |
| --- |

T2_ApptYN
You told us earlier that you had an appointment scheduled on ${e://Field/Appt_MMDDYYYY}.
 Did you have an appointment with a valve specialist on that day?

- Yes
- No, my appointment was rescheduled (click Next to tell us when).
- No, my appointment was cancelled. I don't have a new appointment yet.

Display This Question:

If T2_ApptYN = No, my appointment was rescheduled (click Next to tell us when).

T2_ApptNew When is your new appointment scheduled?


 For example, 10/03/2021 would be written as:
 Month (MM) =10
 Day (DD) = 03
 Year (YYYY) = 2021

- Month (MM) ________________________________________________
- Day (DD) ________________________________________________
- Year (YYYY) ________________________________________________

Q3.2 Which cardiology clinician will you be seeing at your new appointment?

- ${e://Field/HCP}
- A different clinician
- I'm not sure who I will be seeing that day

Display This Question:

If Q3.2 = A different clinician

Q108
**Which clinician will you be seeing on your new appointment?**

If you don't have all the information, please do the best you can.

- Provider's Name ________________________________________________
- City ________________________________________________
- State ________________________________________________
- Phone # ________________________________________________

End of Block: Rescheduled Visit info

Start of Block: Appt Information (Clinician info)

Display This Question:

If T2_ApptYN = Yes

| 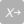 |
| --- |

Q5.1 Did you see ${e://Field/HCP} on your recent cardiology appointment?

- Yes
- No, I saw a different clinician

Display This Question:

If Q5.1 = No, I saw a different clinician

Q5.2
**Which healthcare provider did you see on ${e://Field/Appt_MMDDYYYY}?** If you saw more than one provider, please tell us about the person with whom you spent the most time discussing aortic stenosis treatments. 
 
If you don't have all the information, please do the best you can.

- Provider's Name ________________________________________________
- City ________________________________________________
- State ________________________________________________
- Phone # ________________________________________________

Display This Question:

If T2_ApptYN = Yes

Q5.3 What type of appointment did you have with your clinician?

- In-person
- Telehealth or online (such as Zoom)
- Other (please explain) ________________________________________________

End of Block: Appt Information (Clinician info)

Start of Block: Rescheduled and Cancelled Appointment Messages

Display This Question:

If T2_ApptYN = No, my appointment was rescheduled (click Next to tell us when).

Q4.1 Thanks for letting us know about the change in your appointment. We will contact you on ${e://Field/Appt_MMDDYYYY}.

Display This Question:

If T2_ApptYN = No, my appointment was cancelled. I don't have a new appointment yet.

Q175 Thanks for letting us know about the change in your appointment. Please contact us when you reschedule your appointment. If we don't hear from you, we may reach out to you later.

End of Block: Rescheduled and Cancelled Appointment Messages

Start of Block: Summary Report

| 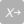 | 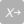 |
| --- | --- |

Q6.2 The next questions ask about your recent cardiology visit. Your responses will be kept strictly confidential; your clinicians will NOT see your responses.  Please answer as honestly as you can.

|  | Yes | No |
| --- | --- | --- |
| Did you discuss your AVITA summary with your provider? |  |  |
| Did the AVITA summary help you talk to your provider about your goals and preferences? |  |  |

Display This Question:

If Q6.2 = [ Yes ]

|  |
| --- |

T2_Share

How did you try to share your AVITA summary with your health care provider? Please click all that apply.

- I **printed it** and brought it to my appointment.
- The summary was mailed to me and I **brought it with me** to my clinician.
- I sent it through my **patient portal**.
- I shared the email using my **tablet**, mobile device, or smart phone.
- I did not try to share my AVITA summary.
- Other (please explain) ________________________________________________

Display This Question:

If 6.2 =[no]

Q6.8
Why did you not discuss the AVITA summary with your clinician? Choose as many as apply.

- I forgot to.
- There wasn't enough time.
- I was not comfortable talking about it
- I don't remember receiving it.
- I didn't see the point.
- Other ________________________________________________

End of Block: Summary Report

T2_SDMProc Thinking about that cardiology visit, how much did your doctor or other clinician...

|  | Not at all | A little bit | Somewhat | Very well | Extremely well |
| --- | --- | --- | --- | --- | --- |
| ...help you understand your aortic stenosis and health issues? |  |  |  |  |  |
| ...listen to the things that matter most to you about your aortic stenosis? |  |  |  |  |  |
| ... include what matters to you in choosing what to do next? |  |  |  |  |  |

Q8.1 The following questions ask you to evaluate communication with your clinician at your recent visit. None of your clinicians will see any of your responses to any of the questions in this survey. Your responses will be kept strictly confidential.  Please answer as honestly as you can.

T2_Comm On a scale from 0 to 10, where 0 is the worst and 10 is the best, how good was the clinician at:

|  | (The very worst I could imagine) 0 | 1 | 2 | 3 | 4 | 5 | 6 | 7 | 8 | 9 | (The very best I could imagine) 10 |
| --- | --- | --- | --- | --- | --- | --- | --- | --- | --- | --- | --- |
| Using words you understand. |  |  |  |  |  |  |  |  |  |  |  |
| Looking you in the eye. |  |  |  |  |  |  |  |  |  |  |  |
| Answering all questions about your illness. |  |  |  |  |  |  |  |  |  |  |  |
| Listening to what you have to say. |  |  |  |  |  |  |  |  |  |  |  |
| Caring about you as a person. |  |  |  |  |  |  |  |  |  |  |  |
| Giving full attention. |  |  |  |  |  |  |  |  |  |  |  |

| 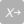 | 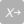 |
| --- | --- |

_MDComm10 **Overall, how would you rate this doctor's communication with you?** On the scale below, 0 is "the very worst" and 10 is "the very best".  Your doctor will **not** see your response to this question (or any other questions in this survey).

- 0 (The very worst)
- 1
- 2
- 3
- 4
- 5
- 6
- 7
- 8
- 9
- 10 (The very best)

| 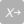 |
| --- |

T2_SDMProcess1 Did the health care provider **explain there were choices** in what you could do to treat your aortic stenosis?

- Yes
- No

|  |
| --- |

T2_SDMProcess2 How much did you and the health care provider talk about the reasons you might want to have TAVR?

- A lot
- Some
- A little
- Not at all

| 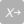 |
| --- |

T2_SDMProcess3 How much did you and the health care provider talk about the reasons you might want to have SAVR?

- A lot
- Some
- A little
- Not at all

| 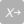 |
| --- |

T2_SDMProcess4 How much did you and the health care provider talk about the reasons you might not want to have TAVR?

- A lot
- Some
- A little
- Not at all

| 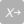 |
| --- |

T2_SDMProcess5 How much did you and the health care provider talk about the reasons you might not want to have SAVR?

- A lot
- Some
- A little
- Not at all

T2_SDMProcess6 Did the health care provider ask you whether or not you wanted to have SAVR or TAVR?

- Yes
- No

End of Block: Patient_HCP Communication

Start of Block: Decision Conflict_Tx decision

T2_SURE At this time, when thinking about choosing treatment for your aortic stenosis...

|  | Yes | No |
| --- | --- | --- |
| Do you know the benefits and risks of each option? |  |  |
| Are you clear about which benefits and risks matter most to you? |  |  |
| Do you have enough support and advice to make a choice? |  |  |
| Do you feel SURE about the best choice for you? |  |  |

| 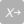 |
| --- |

Q9.2 Did the AVITA questionnaire help you choose treatment for aortic stenosis?

- Yes, a lot
- Yes, a little
- No

T2_DStage In choosing a treatment for your aortic stenosis, would you say that you...

- Have not begun to think about the choices.
- Have not begun to think about the choices but are interested in doing so.
- Are considering the options now.
- Are close to selecting an option.
- Have already made a decision but are still willing to reconsider.
- Have already made a decision and are unlikely to change your mind.

Display This Question:

If T2_DStage = Are close to selecting an option.

Or T2_DStage = Have already made a decision, but are still willing to reconsider.

Or T2_DStage = Have already made a decision and are unlikely to change your mind.

Q9.4 Which treatment have you chosen?

- TAVR (Transcatheter aortic valve replacement)
- SAVR (Surgical aortic valve replacement)
- No valve replacement (medications and/or comfort care)
- Unsure

| 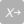 |
| --- |

Q9.5 How confident are you that this treatment plan ${Q9.4/ChoiceGroup/SelectedChoices} is right for you?

- Not at all confident
- A little bit confident
- Somewhat confident
- Very confident
- Completely confident

| 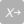 | 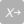 |
| --- | --- |

T2_DQual Please indicate how strongly you agree or disagree with the following statements about your preferred treatment plan:

|  | Strongly disagree | Somewhat disagree | Neither agree nor disagree | Somewhat agree | Strongly agree |
| --- | --- | --- | --- | --- | --- |
| My treatment plan will help me achieve my treatment goals. |  |  |  |  |  |
| My treatment plan reflects what’s important to me when I think about the pros and cons of treatment. |  |  |  |  |  |

Display This Question:

If Q9.4 = TAVR (Transcatheter aortic valve replacement)

Or Q9.4 = SAVR (Surgical aortic valve replacement)

| 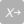 |
| --- |

Q9.6 Is your ${Q9.4/ChoiceGroup/SelectedChoices} scheduled?

- Yes
- No

Display This Question:

If Q9.6 = No

Q9.7 Why is your [SAVR/TAVR] ${Q9.4/ChoiceGroup/SelectedChoices} not scheduled? You can select more than one response.

- I am waiting for a call-back to schedule it
- I need more time to decide
- I need to finish my evaluation with another member of my heart team
- I need to have more medical tests done
- I need to talk it over with family members/caregiver/etc
- I need to check with my insurance
- I want to get a second opinion
- I'm scared or nervous
- I don't want to deal with it right now.
- I'm worried about getting COVID
- Other (please explain) ________________________________________________

Display This Question:

If Q9.6 = No

|  |
| --- |

Q9.8 Do you have another upcoming appointment with your heart team to discuss treatment for your aortic stenosis?

- Yes
- No
- Unsure

|  |
| --- |

Q9.9 Did the clinician your recently saw make a treatment recommendation for your aortic stenosis?

- Yes
- No

Display This Question:

If Q9.9 = Yes

|  |  |
| --- | --- |

Q9.10 Which treatment did your clinician recommend?

- TAVR (Transcatheter aortic valve replacement)
- SAVR (Surgical aortic valve replacement)
- No valve replacement (medications and/or comfort care)

Display This Question:

If Q9.9 = Yes

|  |
| --- |

Q9.11 Do you agree with this clinician's recommendation?

- Yes
- No
- Unsure (please explain) ________________________________________________

|  |
| --- |

Q9.12
Do you trust this clinician's judgment about your medical care?

- Yes
- No
- Unsure

End of Block: Decision Conflict_Tx decision

Start of Block: Actual Role

Q10.1
How involved do you feel you were in making decisions about your treatment for aortic stenosis?

- Not at all involved, others made decisions for me
- A fair bit involved
- Very involved, I made all the decisions myself

| Page Break |  |
| --- | --- |

End of Block: Actual Role

Start of Block: Quality of Care (CAHPS)

| 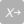 |
| --- |

Q11.1 Using any number from 0 to 10, where 0 is the worst clinician possible and 10 is the best clinician possible, what number would you use to **rate the cardiology** clinician that you recently saw?

- 0
- 1
- 2
- 3
- 4
- 5
- 6
- 7
- 8
- 9
- 10

| Page Break |  |
| --- | --- |

| 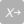 | 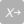 |
| --- | --- |

T2_CAPS During your recent cardiology visit, did your clinician:

|  | Yes, definitely | Yes, somewhat | No |
| --- | --- | --- | --- |
| Listen carefully to you? |  |  |  |
| Spend enough time with you? |  |  |  |
| Treat you with courtesy and respect? |  |  |  |
| Explain things in a way that was easy to understand? |  |  |  |
| Encourage you to ask questions? |  |  |  |
| Tell you there was more than one way to treat your condition? |  |  |  |
| Ask which way to treat your condition you thought was best for you? |  |  |  |
| Talk with you about the risks and benefits of your treatment choices? |  |  |  |
| Give you enough information on your treatment? |  |  |  |

End of Block: Quality of Care (CAHPS)

Start of Block: Knowledge Subjective, Objective

| 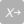 |
| --- |

Q17.1 At this time, how would you rate your knowledge of the following?

|  | Extremely knowledgeable | Very knowledgeable | Moderately knowledgeable | Slightly knowledgeable | Not knowledgeable at all |
| --- | --- | --- | --- | --- | --- |
| Your aortic stenosis |  |  |  |  |  |
| Your options for treating aortic stenosis |  |  |  |  |  |

Know.
The statements below are treating about aortic stenosis. Some are true and some are false. We don't expect you to know all the answers. Please give your best guess when answering them.

|  | True | False |
| --- | --- | --- |
| One choice is to take medicines and not have a valve replacement. |  |  |
| I am more likely to feel better with a valve replacement compared to medicines alone. |  |  |
| Medicines alone (without valve replacement) will help me live longer. |  |  |
| People who get a mechanical valve need to take blood thinners for the rest of their life. |  |  |
| Mechanical valves are just as likely to need replacement as tissue valves. |  |  |
| There is an age limit for getting TAVR |  |  |

End of Block: Knowledge Subjective, Objective

Start of Block: second appointment

| 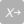 |
| --- |

Q15.1 Have you scheduled another appointment with a different cardiologist to discuss aortic treatment options?

- Yes
- No

Display This Question:

If Q15.1 = Yes

| 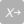 |
| --- |

Q15.2 Would you like to participate in the study for your next cardiology visit?

- Yes, I'm interested
- No, I'm not interested

Display This Question:

If Q15.1 = Yes

Q15.3 What day is your new appointment scheduled?

________________________________________________________________

End of Block: second appointment

Start of Block: Follow up and Feedback

Q16.1 If you have any thoughts or suggestions about AVITA Study, please write them here.

When you are done, please click the next (>>) button to finish the survey.

________________________________________________________________

________________________________________________________________

________________________________________________________________

________________________________________________________________

________________________________________________________________

End of Block: Follow up and Feedback

### Appendix L4: Pilot Study T2 HCP Survey

**HCP - AS Pilot Post-Appointment**

**Start of Block: ICF**

Q1.1  
 

Dear ${e://Field/HCP},
One of your patients with severe aortic stenosis (${e://Field/FirstName_AS} ${e://Field/LastName_AS}) is participating in a research study that tests a new **shared decision-making tool *called AVITA*, A**ortic **V**alve **I**mproved **T**reatment **A**pproaches). We seek your perspective on the AVITA tool. Because this is part of a research study, your consent to participate is requested on the next page.

| Page Break |  |
| --- | --- |

Q1.2 **RESEARCH PARTICIPANT CONSENT FORM**
 **RESEARCH PARTICIPANT CONSENT FORM**
Title:  The AVITA Pilot Study
Protocol No.:     HCP-8243002
Sponsor:           Shared Decision Making Resources under a Scientific Research Agreement with Edwards Lifesciences
Investigator:      Nananda Col, MD, MPH, MPP, FACP
                1119 Five Islands Road
                Georgetown, ME 04548
                United States (USA)
Study-Related
Phone Number:           1 (207) 272-9829 (Nananda Col)
                              
**DETAILED RESEARCH CONSENT**
**What should I know about this research?**

- Taking part in this research is voluntary. There are no consequences if you choose not to participate.
- The shared decision making tool, AVITA, creates a “snapshot” summary of your patient’s goals and preferences and shares it with their heart team to facilitate shared decision making.
- Whether or not you received or reviewed the snapshot summary, we ask you to participate.
- Participation involves completing a brief online survey, which should take about 2-5 minutes.
- You are invited to ask all of your questions before you decide whether you want to participate. Contact information is listed below.
- About 25 patients with severe aortic stenosis and who have an upcoming appointment (within 4 weeks) and their heart team clinicians will be invited to participate in this study.
- The website, Valvesurvey.com, describes the study in more detail.

**Why is this research being done?**

- We are testing an online shared decision making tool called AVITA, designed to help people with severe aortic stenosis explore their treatment options.
- We want to find out if AVITA facilitates shared decision making.

**How long will I be in this research?**

- Your participation in this research will last for the 2-5 minutes it takes to complete this evaluation. If you have other patients participating in the study, you will be asked to consent and complete another brief evaluation.

**What happens to me if I agree to take part in this research?**

- You will be asked a few questions (online) about yourself and the clinic visit with your patient who is in the study. This should take just a few minutes.

**What are my responsibilities if I take part in this research?**

- To complete a brief survey, which follows this page.

**Could being in this research hurt me?**

- We do not anticipate that this research can hurt you.
- To the best of our ability, all of the information you give us will remain completely confidential but there is a risk of loss of confidentiality through data entered online or audio recorded. We take many steps to minimize the risk of losing confidentiality by encrypting data, using secure software, separating personal identifiers from stored data, and restricting access to the data only to the study team.

**Will it cost me money to take part in this research?**

- We do not expect that you will incur any expenses while completing these online surveys. We do, however, offer a small compensation for your effort (see below).

**Will being in this research benefit me?**

- There are no direct benefits to participants other than the possibility that the AVITA tool may improve the conversation you have with your patient about treatment. However, your participation may help us learn more about what matters to patients and improve decision-making tools for aortic stenosis.

**What other choices do I have besides taking part in this research?**

- You may choose to not participate in this study without penalty.

**What happens to the information collected for this research?**

- Records of your participation in this study will be held confidential so far as permitted by law. However, the study principal investigator and, under certain circumstances, WCG Independent Review Board (wcgirb.com, the oversight organization) will be able to inspect and have access to confidential data that identifies you by name. Any publication or presentation of the data will not identify you.

**Who can answer my questions about this research?**

- Please direct any questions to the research team at info@valvesurvey.com or to Dr. Nananda Col at (207) 272-9829 or Dr. Megan Coylewright at Megan.coylewright@erlanger.org.
- If you have any concerns about your rights as a study participant or the way that you have been treated, talk to the research team at the phone number listed above. This research is being overseen by WCG IRB. You may talk to them at 855-818-2289 or researchquestions@wcgirb.com if:

●   You have questions, concerns, or complaints that are not being answered by the research team.
●   You are not getting answers from the research team.
●   You cannot reach the research team.
●   You want to talk to someone else about the research.
●   You have questions about your rights as a research participant.

**What if I am injured because of taking part in this research?**

- We do not expect that you will be hurt by taking part in this research study. No funds have been set aside for payments or other forms of compensation (such as for lost wages, lost time, or discomfort) other than compensation for your participation (see below). However, you do not give up your legal rights by signing this consent form.

**Can I be removed from this research without my approval?**

- We will tell you about any new information that may affect your choice to participate in this research. We may end your participation in this study for any of the following reasons:
- If the study is cancelled by the sponsor or the WCG IRB; or
- For administrative reasons.

**What happens if I agree to be in this research, but I change my mind later?**

- If you decide to leave this research study, please contact the research team (info@valvesurvey.com) so that we can remove you from the study contact list and provide follow-up information if needed.

**Will I be paid for taking part in this research?**

- As compensation for your participation, you will be emailed an Amazon gift card worth $75.

**Statement of Consent:**
I have read the above consent form and agree to participate in this study and I am over 18 years of age.

- Yes, I agree
- No, I do not agree

Display This Question:

If Please review the document below and click “yes” to agree to participate.  RESEARCH PARTICIPANT C... = Yes, I agree

Q260 Please provide your full name and email. We will use this information to send you your Amazon Gift Card.  

- First Name ________________________________________________
- Last Name ________________________________________________
- Email ________________________________________________

Display This Question:

If Please review the document below and click “yes” to agree to participate.  RESEARCH PARTICIPANT C... = No, I do not agree

Q1.3 In order to participate in the study, you must give informed consent. If you would like to reconsider your response, please click << to return to review the consent document. If you have any questions about the study, please feel free to contact Dr. Nananda Col at 207-272-9829.

End of Block: ICF

| 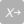 |
| --- |

Q2.1 The following questions pertain to your recent visit with ${e://Field/FirstName_AS} ${e://Field/LastName_AS}.  Did you see this patient recently?

- Yes
- No, I did not see that patient.

Display This Question:

If The following questions pertain to your recent visit with ${e://Field/FirstName_AS} ... = No, I did not see that patient.

Q2.2 Thank you for your time.

Skip To: End of Survey If Thank you for your time. Is Displayed

| 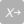 |
| --- |

Q3.1 What recommendation are you leaning towards for this patient at this time?  Please select one.

- Medical management/palliative care
- SAVR
- TAVR

Q3.2 Did the AVITA summary influence your recommendation?

- Yes, a lot
- Yes, a little
- No

|  |
| --- |

Q3.3 What role do you think your patient **wanted** to play in choosing treatment for aortic stenosis?

- My patient wanted to make the decision alone.
- My patient wanted to make the decision after seriously considering my opinion.
- My patient wanted to share responsibility with me.
- My patient wanted me to make the final decision after seriously considering their opinion.
- My patient wanted to leave the decision to me.

| 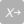 |
| --- |

Q6.3
How much did you talk to your patient about ...

|  | A lot | Some | A little | Not at all |
| --- | --- | --- | --- | --- |
| The **benefits of TAVR**? |  |  |  |  |
| The **risks of TAVR**? |  |  |  |  |
| The **benefits of SAVR**? |  |  |  |  |
| The **risks of SAVR**? |  |  |  |  |

End of Block: HCP recommendation and patient role

Q4.1 Did you review your patient's AVITA summary? The summary looks like this (this is just a sample; it is not your patient's summary):   

- I reviewed the summary and used it during the visit.
- I reviewed the summary but did not use it during the visit.
- I reviewed it after the visit.
- I saw it but did not review it.
- I never received the patient's summary.

Display This Question:

If Did you review your patient's AVITA summary? The summary looks like this (this is just a sample;... = I reviewed the summary and used it during the visit.

Or Did you review your patient's AVITA summary? The summary looks like this (this is just a sample;... = I reviewed the summary but did not use it during the visit.

Or Did you review your patient's AVITA summary? The summary looks like this (this is just a sample;... = I reviewed it after the visit.

Or Did you review your patient's AVITA summary? The summary looks like this (this is just a sample;... = I saw it but did not review it.

|  |
| --- |

Q4.2 How did you receive the patient summary? Check all that apply.

- It was handed to me by the patient.
- It was printed for me and made available at the time of the clinic visit.
- It was emailed directly to me.
- It was sent to me via the patient portal.
- The patient shared it with me on their phone or mobile device.
- Other (please explain) ________________________________________________

| 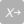 |
| --- |

Q4.3 How would you prefer to receive the patient summary in the future? Check all that apply.

- Have the patient hand it to me.
- Have staff print and make it available at the time of the clinic visit.
- Email it to me and/or my coordinator.
- Send to me via the patient portal.
- Have the patient share on their phone or mobile device.
- Other (please explain) ________________________________________________

Display This Question:

If Did you review your patient's AVITA summary? The summary looks like this (this is just a sample;... = I reviewed the summary and used it during the visit.

Or Did you review your patient's AVITA summary? The summary looks like this (this is just a sample;... = I reviewed the summary but did not use it during the visit.

Or Did you review your patient's AVITA summary? The summary looks like this (this is just a sample;... = I reviewed it after the visit.

| 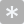 |
| --- |

Q4.4 How many minutes did you spend reviewing the patient summary?

________________________________________________________________

Display This Question:

If Did you review your patient's AVITA summary? The summary looks like this (this is just a sample;... = I saw it but did not review it.

| 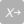 |
| --- |

Q4.5 Why did you **not** review the patient summary? Choose as many as apply.

- I felt I did not have enough **time**.
- I already know this patient and their preferences very well, I didn't think it would add more.
- I wanted to but I forgot.
- My patient preferred that I made the decision based on technical factors. [my patient did not want to be involved in decision making*]
- Patient was not suitable for other treatment options.
- I felt patient preferences weren't relevant in this case.
- Other (please explain) ________________________________________________

Display This Question:

If Did you review your patient's AVITA summary? The summary looks like this (this is just a sample;... = I reviewed the summary and used it during the visit.

Or Did you review your patient's AVITA summary? The summary looks like this (this is just a sample;... = I reviewed the summary but did not use it during the visit.

Q4.6
Did you feel that this visit was more or less efficient because of the AVITA tool?

- More efficient
- No change
- Less efficient

End of Block: Logistics and timing

Start of Block: HCP Impact on misc

| 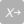 | 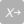 |
| --- | --- |

Q5.1 During the visit with ${e://Field/FirstName_AS} ${e://Field/LastName_AS}, what impact did the patient summary have on the following:

|  | Improved | No impact | Worsened |
| --- | --- | --- | --- |
| Your knowledge of what's important to your patient. |  |  |  |
| Your ability to engage your patient in decision making. |  |  |  |
| Your communication with your patient. |  |  |  |
| Your ability to make a valve recommendation based on what's important to the patient. |  |  |  |

Q5.2 Would you use the AVITA summary in future encounters?

- Yes, if the patient brings it to us.
- Yes, if we make it part of our routine.
- No, it was not helpful
- No, I think routine use would be too challenging to coordinate.

End of Block: HCP Impact on misc

Start of Block: SDM global impact

| 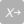 |
| --- |

Q7.1 **Shared decision making** is a conversation between two experts where the clinician provides **evidence-based information** **and clinical experience** and the **patient provides expertise on their values and preferences as they compare the benefits, harms, and risks of each option**. This leads to a meaningful dialogue about what matters most to the patient. 


Did you practice shared decision making with ${e://Field/FirstName_AS} ${e://Field/LastName_AS} today?

- Yes
- No

Display This Question:

If Shared decision making is a conversation between two experts where the clinician provides evidenc... = No

Q7.3
What were the challenges that you encountered practicing shared decision making with ${e://Field/FirstName_AS} ${e://Field/LastName_AS}? Select all that apply.

- It was not needed because only one treatment plan was appropriate for my patient.
- It was stressful or overwhelming for this patient.
- I was not trained in shared decision making.
- I was concerned the patient might choose a treatment that I would be uncomfortable supporting.
- I had other things I needed to accomplish during the visit and ran out of time.
- I don't think it benefited this patient.
- My patient did not want to be involved in decision-making.
- My patient lacked the skills or knowledge to participate.
- It disrupted work flow.
- Other (please explain) ________________________________________________

Display This Question:

If Shared decision making is a conversation between two experts where the clinician provides evidenc... = No

Carry Forward Selected Choices from "What were the challenges that you encountered practicing shared decision making with ${e://Field/FirstName_AS} ${e://Field/LastName_AS}? Select all that apply.  "

Q74 Please rank order the challenges that you encountered practicing shared decision making with ${e://Field/FirstName_AS} ${e://Field/LastName_AS} by dragging the items in order of importance, with 1 being the most important.

______ It was not needed because only one treatment plan was appropriate for my patient.

______ It was stressful or overwhelming for this patient.

______ I was not trained in shared decision making.

______ I was concerned the patient might choose a treatment that I would be uncomfortable supporting.

______ I had other things I needed to accomplish during the visit and ran out of time.

______ I don't think it benefited this patient.

______ My patient did not want to be involved in decision-making.

______ My patient lacked the skills or knowledge to participate.

______ It disrupted work flow.

______ Other (please explain)

Display This Question:

If Shared decision making is a conversation between two experts where the clinician provides evidenc... = Yes

|  |
| --- |

Q7.4
Why did you practice shared decision making with ${e://Field/FirstName_AS} ${e://Field/LastName_AS}? Select as many as apply. 

- To improve the **quality of care** that I provide.
- To help my patient **choose a treatment**.
- My **patient wanted to** be involved in decision-making.
- To help my patient **understand the reasons** behind my recommendation.
- To adhere to **clinical guidelines**.
- To comply with the **TVT registry**.
- It's **my usual style** of practice.
- To **benefit my patient.**
- To better **understand** my patient.
- Other ________________________________________________

Display This Question:

If Shared decision making is a conversation between two experts where the clinician provides evidenc... = Yes

Carry Forward Selected Choices from "Why did you practice shared decision making with ${e://Field/FirstName_AS} ${e://Field/LastName_AS}? Select as many as apply.   "

| 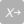 |
| --- |

Q73 Please rank order the reasons why you practiced shared decision making with ${e://Field/FirstName_AS} ${e://Field/LastName_AS} by dragging the items in order of importance, with 1 being the most important.

______ To improve the **quality of care** that I provide.

______ To help my patient **choose a treatment**.

______ My **patient wanted to** be involved in decision-making.

______ To help my patient **understand the reasons** behind my recommendation.

______ To adhere to **clinical guidelines**.

______ To comply with the **TVT registry**.

______ It's **my usual style** of practice.

______ To **benefit my patient.**

______ To better **understand** my patient.

______ Other

Q7.5 Who do you think **should** **be responsible for ensuring that your patients engage in shared decision making**? Check all that apply.

- The physician who will perform the recommended procedure.
- A physician who will **not** be performing the recommended procedure.
- An APP with experience in this area.
- The referring cardiologist.
- Other (please specify) ________________________________________________

Q7.6 Do you have any suggestions for making it easier to implement shared decision making in your clinic?

________________________________________________________________

End of Block: SDM global impact

Start of Block: HCP Information

Q8.1 Have you completed this survey for a previous patient in the ASPIRE Study?

- Yes
- No

Skip To: End of Block If Have you completed this survey for a previous patient in the ASPIRE Study? = Yes

| 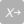 |
| --- |

Q8.2 What is your gender?

- Male
- Female
- Non-binary
- Prefer not to answer

| 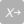 |
| --- |

Q8.3 What is your present position?

- Cardiologist
- Interventional cardiologist
- Cardiothoracic surgeon
- Attending physician, not a cardiologist
- Nurse Practitioner
- Physician Assistant
- Fellow
- Intern or resident
- Other (please specify) ________________________________________________

Q8.4 Are you affiliated with a Structural Heart Team?

- Yes (please specify which center) ________________________________________________
- No

|  |
| --- |

Q8.5 How many years have you been in practice after completing residency or training?

________________________________________________________________

Q9.2

**Thank you** for taking the time to be part of our study. We hope that you had a good experience.  
We will email you an online gift card within the next 2-3 business days as a token of our appreciation. It will arrive directly from Amazon so you may need to check your spam or trash email folders. If you would prefer that we email the gift certificate to someone other than yourself, please let us know by selecting the option below.

- Please send my gift certificate to someone other than myself. (Please enter their email in the box.) ________________________________________________

End of Block: Feedback, ending

### Appendix M. Study Website—ValveSurvey.com (Screen shots of all pages)

Home Page:

Tab “The Study”

Page “In a nutshell”

Page “About this study”

Page “About this study” (continued)

Page “About our study, sponsor, and partners”

Page “About Our Team”

Page “About our team” (continued)

Tab “For Patients”

Page “What will happen-Patients”

Page “Who is eligible”

Page “Informed Consent”

Tab “For Providers”

Page “What will happen- clinician/heart team

Page “What will happen- clinician/heart team (continued)

Page “Informed Consent”

Page “How to refer patients to the AVITA study”

Page “Scientific underpinnings and publications”

Page “Scientific underpinnings and publications” (continued)

Tab “Enroll”

Page “Enroll”

Tab “Contact Us”

# References Cited

1. Leon MB, Smith CR, Mack M, et al. Transcatheter aortic-valve implantation for aortic stenosis in patients who cannot undergo surgery. *N Engl J Med.* 2010;363(17):1597-1607. [↑](#endnote-ref-2)
2. ClinicalTrials.gov. The PARTNER 3 - Trial - The Safety and Effectiveness of the SAPIEN 3 Transcatheter Heart Valve in Low Risk Patients With Aortic Stenosis (P3). 2017. [↑](#endnote-ref-3)
3. ClinicalTrials.gov. Medtronic Transcatheter Aortic Valve Replacement in Low Risk Patients. 2017. [↑](#endnote-ref-4)
4. Waksman R, Rogers T, Torguson R, et al. Transcatheter Aortic Valve Replacement in Low-Risk Patients with Symptomatic Severe Aortic Stenosis. *Journal of the American College of Cardiology.* 2018. [↑](#endnote-ref-5)
5. Otto CM, Kumbhani DJ, Alexander KP et al. 2017 ACC Expert Consensus Decision Pathway for Transcatheter Aortic Valve Replacement in the Management of Adults With Aortic Stenosis: A Report of the American College of Cardiology Task Force on Clinical Expert Consensus Documents. J Am Coll Cardiol. 2017; 69(10):1313-46.. [↑](#endnote-ref-6)
6. Stacey D, Légaré F, Col NF, Bennett CL, Barry MJ, Eden KB, Holmes-Rovner M, Llewellyn-Thomas H, Lyddiatt A, Thomson R, Trevena L, Wu JHC. Decision aids for people facing health treatment or screening decisions. Cochrane Database Syst Rev. 2014 Jan 28;(1):CD001431. [↑](#endnote-ref-7)
7. Légaré F, Politi M, Drolet R, et al. Training health professionals in shared decision making: An international environmental scan. Pt Educ Couns. 2012;88:159-69. [↑](#endnote-ref-8)
8. Street RL Jr, Gordon HS, Ward MM, Krupat E, Kravitz RL. Patient participation in medical consultations: why some patients are more involved than others. *Med Care.* 2005;43:960-969. [↑](#endnote-ref-9)
9. Peters E, Dieckmann N, Dixon A, Hibbard JH, Mertz CK. Less is more in presenting quality information to consumers. Medical Care Research and Review. 2007 Apr 1;64(2):169-90. [↑](#endnote-ref-10)
10. Lee CN, Hultman CS, Sepucha K. Do patients and providers agree about the most important facts and goals for breast reconstruction decisions? Ann Plast Surg. 2010 May;64(5):563-6. doi: 10.1097/SAP.0b013e3181c01279. [↑](#endnote-ref-11)
11. Devereaux PJ, Anderson DR, Gardner MJ, Putnam W, Flowerdew GJ, Brownell BF, et al: Differences between perspectives of physicians and patients on anticoagulation in patients with atrial fibrillation: observational study. BMJ 2001, 323:1218-22. [↑](#endnote-ref-12)
12. Jecker NS. The role of intimate others in medical decision making. Gerontol 1990;30:65–71. [↑](#endnote-ref-13)
13. Hornberger JC, Habraken H, Bloch DA. Minimum data needed on patient preferences for accurate, efficient medical decision making. Med Care 1995;33:297–310. [↑](#endnote-ref-14)
14. Dolan JG. Patient priorities in colorectal cancer screening decisions. Health Expect. 2005;8(4):334-44. [↑](#endnote-ref-15)
15. Coast J, Al-Janabi H, Sutton EJ, et al. Using qualitative methods for attribute development for discrete choice experiments: issues and recommendations. Health Econ 2012;21:730–41. [↑](#endnote-ref-16)
16. Marshall D, Bridges JF, Hauber B. Conjoint analysis application in health—how are studies being designed and reported? An update on current practice in the published literature between 2005 and 2008. Patient 2010;3:249–56. [↑](#endnote-ref-17)
17. Hollin IL, Young C, Hanson C, Bridges JF, Peay H. Developing a Patient-Centered Benefit-Risk Survey: A Community-Engaged Process. Value in Health. 2016 Oct 31;19(6):751-7. [↑](#endnote-ref-18)
18. https://www.cardiosmart.org/SDM/Decision-Aids/Find-Decision-Aids/Aortic-Stenosis [↑](#endnote-ref-19)
19. Available online at valveadvice.org [↑](#endnote-ref-20)
20. Available online at sharedcardiology.org [↑](#endnote-ref-21)
21. Col NF, Solomon AJ, Springmann V, Garbin C, Ionete C, Pbert L, Alvarez E, Tierman B, Hopson A, Kutz C, Berrios Morales I, Griffin C, Phillips G, Ngo L. Whose preferences matter? a patient-centered approach for eliciting treatment goals. Med Decis Making. 2018;38:44-55. [↑](#endnote-ref-22)
22. Col NF, Solomon AJ, Springmann V, Ionete C, Alvarez E, Tierman B, Kutz C, Berrios Morales I, Griffin C, Ngo L, Jones DE, Phillips G, Pbert L. Evaluation of a Novel Preference Assessment Tool for Patients with Multiple Sclerosis. In press, International Journal of MS Care, 2018. [doi:10.7224/1537-2073.2017-021](https://doi.org/10.7224/1537-2073.2017-021). Online first available at http://ijmsc.org/doi/pdf/10.7224/1537-2073.2017-021 [↑](#endnote-ref-23)
23. Col NF, Alvarez E, Springmann V, Ionete C, Berrios Morales I, Solomon A, Kutz C, Griffin C, Tierman B, Livingston T, Patel M, van Leeuwen D, Ngo L, Pbert L. A Novel Tool to Improve Shared Decision Making and Adherence in Multiple Sclerosis: A Pilot Study. [MDM Policy Pract](https://www.ncbi.nlm.nih.gov/pmc/articles/PMC6798166/). 2019 Jul-Dec; 4(2): 2381468319879134. https://journals.sagepub.com/doi/full/10.1177/2381468319879134 [↑](#endnote-ref-24)
24. Delbecq, A., Van de Ven, A.H., Gustafson DH. Group Techniques for Program Planning: A guide to Nominal Group and Delphi Processes. Glenview: Scott Foresman 1975. [↑](#endnote-ref-25)
25. Valentine KD, Vo H, Fowler FJ Jr, Brodney S, Barry MJ, Sepucha KR. Development and Evaluation of the Shared Decision Making Process Scale: A Short Patient-Reported Measure. Med Decis Making. 2021 Feb;41(2):108-119. doi: 10.1177/0272989X20977878. Epub 2020 Dec 15. PMID: 33319648. [↑](#endnote-ref-26)
26. Brodney S, Fowler FJ Jr, Barry MJ, Chang Y, Sepucha K. Comparison of Three Measures of Shared Decision Making: SDM Process_4, CollaboRATE, and SURE Scales. Med Decis Making. 2019 Aug;39(6):673-680. doi: 10.1177/0272989X19855951. Epub 2019 Jun 21. PMID: 31226911; PMCID: PMC6791732. [↑](#endnote-ref-27)
27. Barr PJ, Thompson R, Walsh T, Grande SW, Ozanne EM, Elwyn G. The psychometric properties of CollaboRATE: a fast and frugal patient-reported measure of the shared decision-making process [published correction appears in J Med Internet Res. 2015;17(2):e32] [published correction appears in J Med Internet Res. 2015;17(2):e32]. *J Med Internet Res*. 2014;16(1):e2. Published 2014 Jan 3. doi:10.2196/jmir.3085 [↑](#endnote-ref-28)
28. Lee CN et al. Development of instruments to measure the quality of breast cancer treatment decisions. Health Expect, 2010: 13(3), 258‐ 72. [↑](#endnote-ref-29)
29. Legéré, F., 2010 et al. Are you SURE? Assessing patient decisional conflict with a 4-item screening test, [online] Available at: http://www.cfp.ca/content/56/8/e308.full [↑](#endnote-ref-30)
30. <https://www.ahrq.gov/sites/default/files/wysiwyg/cahps/surveys-guidance/surgical/measures-surgical-409.pdf> [↑](#endnote-ref-31)
31. Col NF, Alvarez E, Springmann V, Ionete C, Berrios Morales I, Solomon A, Kutz C, Griffin C, Tierman B, Livingston T, Patel M, van Leeuwen D, Ngo L, Pbert L. A Novel Tool to Improve Shared Decision Making and Adherence in Multiple Sclerosis: A Pilot Study. Under review, Medical Decision Making Policy & Practice, 2018. [↑](#endnote-ref-32)
32. Col NF. Patient health communication to improve shared decision making. In: Fischhoff B, Brewer NT, Downs JS, eds. [*Communicating Risks and Benefits: An Evidence-Based User's Guide.*](http://www.fda.gov/downloads/AboutFDA/ReportsManualsForms/Reports/UCM268069.pdf)  Bethesda, MD: US Department of Health and Human Services, Food and Drug Administration, Risk Communication Advisory Committee and consultants. 2011 [↑](#endnote-ref-33)
33. <https://www.cms.gov/Research-Statistics-Data-and-Systems/Research/CAHPS/> [↑](#endnote-ref-34)
34. http://www.cms.gov/EHRincentivePrograms/ [↑](#endnote-ref-35)
35. Wolfe F, Michaud K, Pincus T. Development and validation of the health assessment questionnaire II: a revised version of the health assessment questionnaire. Arthritis Rheum. 2004;50(10):3296-305. [↑](#endnote-ref-36)
36. Col NF, Solomon A, Jones D, Pbert L, Ionete C, Alvarez E, Berrios Morales I, Kutz C, Springmann V, Griffin C, Tierman B, Livingston T, Patel M, van Leeuwen D, Ngo L. Supporting Shared Decision Making for Multiple Sclerosis Patients and Providers: Sharing and Understanding Personal Preferences and Objectives Regarding Treatment (MS-SUPPORT).

    Next Generation Innovator Abstract, National Quality Forum (NQF), March 12, 2018, Washington, DC. [↑](#endnote-ref-37)
37. Witteman HO, Scherer LD, Gavaruzzi T, Pieterse AH, Fuhrel-Forbis A, Dansokho SC, Exe N, Kahn VC, Feldman-Stewart D, Col NF, Turgeon AF, Fagerin A. Design Features of Explicit Values Clarification Methods: A Systematic Review. Med Decis Making. 2016; 36(4):453-71. [↑](#endnote-ref-38)
38. Witteman H, Gavaruzzi T, Scherer L, Pieterse A, Fuhrel-Forbis A, Chipenda D, Kahn V, Feldman-Stewart D, Col N, Fagerlin A. Effects of Design Features of Explicit Values Clarification Methods: A Systematic Review. Med Decis Making. 2016; 36(6):760-76. [↑](#endnote-ref-39)
39. Shewchuk RM, O’Connor SJ, Fine DJ. (2005). Building an understanding of the competencies needed for health administration practice. *Journal of Healthcare Management,* 50(1), 32-47.PMID: 15729906. [↑](#endnote-ref-40)
40. Shewchuk RM, Schmidt HJ, Benarous A, Bennett NL, Abdolrasulnia M, Casebeer LL.(2007). A standardized approach to assessing physician expectations and perceptions of continuing medical education. *Journal of Continuing Education in the Health Professions*, 27(3), 173-182. PMID: 17876846. [↑](#endnote-ref-41)
41. Valentine KD, Vo H, Fowler FJ Jr, Brodney S, Barry MJ, Sepucha KR. Development and Evaluation of the Shared Decision Making Process Scale: A Short Patient-Reported Measure. Med Decis Making. 2021 Feb;41(2):108-119. doi: 10.1177/0272989X20977878. Epub 2020 Dec 15. PMID: 33319648. [↑](#endnote-ref-42)
42. Brodney S, Fowler FJ Jr, Barry MJ, Chang Y, Sepucha K. Comparison of Three Measures of Shared Decision Making: SDM Process_4, CollaboRATE, and SURE Scales. Med Decis Making. 2019 Aug;39(6):673-680. doi: 10.1177/0272989X19855951. Epub 2019 Jun 21. PMID: 31226911; PMCID: PMC6791732. [↑](#endnote-ref-43)
43. Barr PJ, Thompson R, Walsh T, Grande SW, Ozanne EM, Elwyn G. The psychometric properties of CollaboRATE: a fast and frugal patient-reported measure of the shared decision-making process [published correction appears in J Med Internet Res. 2015;17(2):e32] [published correction appears in J Med Internet Res. 2015;17(2):e32]. *J Med Internet Res*. 2014;16(1):e2. Published 2014 Jan 3. doi:10.2196/jmir.3085 [↑](#endnote-ref-44)
44. Lee CN et al. Development of instruments to measure the quality of breast cancer treatment decisions. Health Expect, 2010: 13(3), 258‐ 72. [↑](#endnote-ref-45)
45. Legéré, F., 2010 et al. Are you SURE? Assessing patient decisional conflict with a 4-item screening test, [online] Available at: http://www.cfp.ca/content/56/8/e308.full [↑](#endnote-ref-46)
46. <https://www.ahrq.gov/sites/default/files/wysiwyg/cahps/surveys-guidance/surgical/measures-surgical-409.pdf> [↑](#endnote-ref-47)
47. Col NF, Alvarez E, Springmann V, Ionete C, Berrios Morales I, Solomon A, Kutz C, Griffin C, Tierman B, Livingston T, Patel M, van Leeuwen D, Ngo L, Pbert L. A Novel Tool to Improve Shared Decision Making and Adherence in Multiple Sclerosis: A Pilot Study. Under review, Medical Decision Making Policy & Practice, 2018. [↑](#endnote-ref-48)
48. Spertus JA, Jones PE. Development and Validation of a Short Version of the Kansas City Cardiomyopathy Questionnaire. Circulation: Cardiovascular Quality & Outcomes, August 2015. [↑](#endnote-ref-49)
49. Shewchuk RM, O’Connor SJ, Fine DJ. (2005). Building an understanding of the competencies needed for health administration practice. *Journal of Healthcare Management,* 50(1), 32-47.PMID: 15729906. [↑](#endnote-ref-50)
50. Shewchuk RM, Schmidt HJ, Benarous A, Bennett NL, Abdolrasulnia M, Casebeer LL.(2007). A standardized approach to assessing physician expectations and perceptions of continuing medical education. *Journal of Continuing Education in the Health Professions*, 27(3), 173-182. PMID: 17876846. [↑](#endnote-ref-51)
51. Shewchuk RM, O’Connor SJ, Fine DJ. (2005). Building an understanding of the competencies needed for health administration practice. *Journal of Healthcare Management,* 50(1), 32-47. PMID: 15729906. [↑](#endnote-ref-52)
52. Shewchuk RM, Schmidt HJ, Benarous A, Bennett NL, Abdolrasulnia M, Casebeer LL.(2007). A standardized approach to assessing physician expectations and perceptions of continuing medical education. *Journal of Continuing Education in the Health Professions*, 27(3), 173-182. PMID: 17876846. [↑](#endnote-ref-53)
53. 5-9 items are all that can be effectively ranked on one dimension of discrimination. [↑](#footnote-ref-1)
54. 5-9 items are all that can be effectively ranked on one dimension of discrimination. [↑](#footnote-ref-2)
